# Purpurogemutantin and Purpurogemutantidin, New Drimenyl Cyclohexenone Derivatives Produced by a Mutant Obtained by Diethyl Sulfate Mutagenesis of a Marine-Derived *Penicillium purpurogenum* G59

**DOI:** 10.3390/md10061266

**Published:** 2012-06-04

**Authors:** Shi-Ming Fang, Cheng-Bin Cui, Chang-Wei Li, Chang-Jing Wu, Zhi-Jun Zhang, Li Li, Xiao-Jun Huang, Wen-Cai Ye

**Affiliations:** 1 Beijing Institute of Pharmacology and Toxicology, Beijing 100850, China; Email: fang_shiming@163.com (S.-M.F.); sdrlcw@sohu.com (C.-W.L.); wucj2009@163.com (C.-J.W.); zzjcust@sina.com (Z.-J.Z.); 2 Key Laboratory of Structure-Based Drug Design & Discovery of Ministry of Education, School of Traditional Chinese Materia Medica, Shenyang Pharmaceutical University, Shenyang 110016, China; 3 State Key Laboratory of Bioactive Substance and Function of Natural Medicines, Institute of Materia Medica, Chinese Academy of Medical Sciences and Peking Union Medical College, Beijing 100050, China; Email: annaleelin@imm.ac.cn; 4 Institute of Traditional Chinese Medicine & Natural Products, Jinan University, Guangzhou 510632, China; Email: zhyxiaohuang@163.com (X.-J.H.); chywc@yahoo.com.cn (W.-C.Y.)

**Keywords:** purpurogemutantin, purpurogemutantidin, sesquiterpene, meroterpenoid, structure determination, antitumor activity, *Penicillium purpurogenum*, marine-derived fungus, DES mutagenesis

## Abstract

Two new drimenyl cyclohexenone derivatives, named purpurogemutantin (**1**) and purpurogemutantidin (**2**), and the known macrophorin A (**3**) were isolated from a bioactive mutant BD-1-6 obtained by random diethyl sulfate (DES) mutagenesis of a marine-derived *Penicillium purpurogenum* G59. Structures and absolute configurations of **1** and **2** were determined by extensive spectroscopic methods, especially 2D NMR and electronic circular dichroism (ECD) analysis. Possible biosynthetic pathways for **1**–**3** were also proposed and discussed. Compounds **1** and **2** significantly inhibited human cancer K562, HL-60, HeLa, BGC-823 and MCF-7 cells, and compound **3** also inhibited the K562 and HL-60 cells. Both bioassay and chemical analysis (HPLC, LC-ESIMS) demonstrated that the parent strain G59 did not produce **1**–**3**, and that DES-induced mutation(s) in the mutant BD-1-6 activated some silent biosynthetic pathways in the parent strain G59, including one set for **1**–**3** production.

## 1. Introduction

Meroterpenoids, generally occurring in nature, display a huge range of structural diversity with a broad spectrum of important biological activities [1]. Drimane or 4,9-friedodrimane meroterpenoids with a quinone or hydroquinone moiety were frequently isolated from marine sponges [2,3,4,5,6,7,8] and algae [9,10,11]. The same class of compounds having a regular drimane skeleton were also often isolated from both plant-associated [12,13,14,15] and marine-derived [16] fungi. Among them, (−)-taurinin [12,13,17], (−)-F-12509A [14,18], (+)-hyatellaquinone [11,19], (+)-zonarol [10,20,21] and (+)-zonarone [10,21,22] were synthesized [17,18,19,20,21,22] to determine their absolute configurations. Relative stereochemistry of the drimenyl unit in these compounds is the same as that in albicanol [23], a simple drimene alcohol with a hydroxymethyl group in the drimene skeleton. However, the absolute configuration of the drimenyl residue is opposite in these compounds, indicating the occurrence of both drimenyl enantiomers in nature. Cyclohexenone derivatives structurally closely related to these drimene quinones are rare in nature, macrophorins A–C [24,25], macrophorin D [26,27], 4′-oxomacrophorin A [27], 4′-oxomacrophorin D [27], macrophorins E–G [28], 2′,3′-epoxy-13-hydroxy-4′-oxomacrophorin A [29], epoxyphomalins A and B [30], and penicilliumin A [31] being examples isolated from plant-associated [24,25,26,27,28] and marine-derived [29,30,31] fungi. Some of these exhibited antimicrobial [24,25,28,29], self-inhibitory [26], immunosuppressive [27], or *in vitro* antitumor [30,31] activities.

Several *Penicillium purpurogenum* strains are known to produce bioactive metabolites with novel structures [32,33,34,35,36], including antitumor metabolites [32]. However, *Penicillium purpurogenum* G59, a marine-derived wild-type strain isolated by our group, was originally not able to produce antitumor metabolites with activity in the MTT assay using K562 cells [37]. It has been well recognized that the main biosynthetic pathways in most microbial strains are silent and thus unable to produce secondary metabolites under usual laboratory culture conditions [38]. Thus, various approaches were developed to awake the silent biosynthetic pathways to access cryptic secondary metabolites. Among them, the one strain many compounds (OSMAC) strategy [39], ribosome engineering [40,41], and chemical epigenetics method [42,43] could be simply applied by natural product chemists owing to their practical experimental procedures. We have also reported a new and simple approach to activate the dormant secondary metabolite production by introducing gentamicin resistance in *P. purpurogenum* G59 [44]. Using this method, we obtained nine antitumor mutants from strain G59 [44], and several antitumor secondary metabolites newly produced by two bioactive mutants were also explored previously [44,45]. Later, we attempted to activate the silent secondary metabolite production in strain G59 by random diethyl sulfate (DES) mutagenesis and succeeded in obtaining an antitumor mutant BD-1-6.

**Figure 1:**
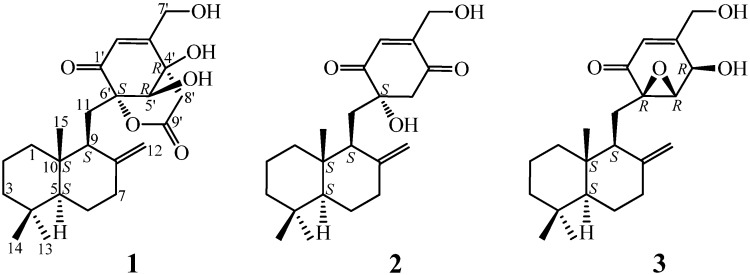
Structures of **1**–**3** from the mutant strain *Penicillium purpurogenum* BD-1-6.

To examine the effect of DES-induced mutation on the secondary metabolite production, we carried out chemical investigation of antitumor secondary metabolites of the mutant BD-1-6. Bioassay-guided fractionation of the BD-1-6 culture extract resulted in the isolation of three antitumor metabolites **1**–**3** (Figure 1), all being newly produced by the mutant BD-1-6 compared to its parent strain G59. Structures of two new compounds, named purpurogemutantin (**1**) and purpurogemutantidin (**2**), were elucidated by various spectroscopic methods and their absolute configurations were determined on the basis of CD and ECD data. The isolation, structure elucidation, cytotoxicity assay, and HPLC and LC-ESIMS analysis for **1**–**3** are reported in detail in this paper.

## 2. Results and Discussion

Fermentation and extraction of the mutant BD-1-6 provided an ethyl acetate extract showing cytotoxicity on K562 cells with an inhibition rate of 58.6% at 100 µg*/*mL. Bioassay-guided column chromatography of the extract delivered active fractions, which were then subjected to a semi-preparative HPLC separation to afford bioactive metabolites **1**–**3**.

Compound **3**, a pale-colored solid, 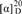 +25.7 (*c* 1.0, MeOH), afforded a molecular weight of 360 Dalton by positive and negative ESI-MSs and was identified as macrophorin A [24] according to the physicochemical and spectroscopic data. Full ^1^H NMR data of **3**are reported for the first time.

### 2.1. Structure Determination of *1* and *2*

Purpurogemutantin (**1**) was obtained as a white crystalline powder from MeOH solution, mp 122–123 °C, 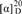 +21.0 (*c* 1.0, MeOH), and its molecular formula C_24_H_34_O_6_ was determined by HRESIMS (*m/z* 419.2431 [M + H]^+^; Δ = +0.3 mmu). Its UV (λ_max_ 234 nm, log ε 3.87) and IR (ν_max_ 1694, 889 cm^−1^) absorptions revealed an α,β-unsaturated ketone chromophore [24] in **1**. The IR spectrum of **1** further indicated the presence of hydroxyl (3405 cm^−1^) and ester carbonyl (1733 cm^−1^) groups. The ^1^H NMR spectrum of **1** in acetone-*d*_6_ showed signals due to three *tert*-methyl groups, three olefinic and three exchangeable protons together with several methine and methylene proton signals (Table 1). The ^13^C NMR spectrum in acetone-*d*_6_, analyzed by DEPT, revealed the presence of a conjugated ketone carbonyl, an ester carbonyl, two sp^2^ and four sp^3^ quaternary carbons, one sp^2^ and three sp^3^ methine, one sp^2^ and eight sp^3^ methylene, and three methyl groups in **1** (Table 1). The ESIMS, HRESIMS, UV, IR, ^1^H and ^13^C NMR, DEPT, ^1^H–^1^H COSY, HMQC, HMBC, and NOESY spectra of **1** are given as supporting information in Supplementary Data S1.

**Table 1:** 400 MHz ^1^H and 100 MHz ^13^C NMR data of **1** in acetone-*d*_6_^a^.

Position	δ_C_^b,c^	δ_H_^b^ (*J* in Hz)	COSY ^d^	NOE ^e^	HMBC ^f^
1	39.3 t	H*a* 1.12 td (13.7, 3.4) H *e* 1.723 br d (13.7)	H*e*-1, H_2_-2 H*a*-1, H*a*-2	H*e*-1, H*e*-2, H*a*-2 ^g^, H*a*-7, H-9 H*a*-1, H_2_-2,Ha-11, H_3_-15	C-3,5
2	19.9 t	H*a* 1.58 qt (13.7, 3.4) H*e* 1.45 dquint (13.7, 3.4)	H_2_-1, H*e*-2, H_2_-3 H*a*-1, H*a*-2, H_2_-3	H*e*-1, H*a*-1 ^g^, H_3_-14, H_3_-15	
3	42.8 t	H*a* 1.14 td (13.7, 3.4) H*e* 1.36 dt (13.7, 3.4)	H_2_-2, H*e*-3 H_2_-2, H*a*-3	H*e*-3, H_3_-13 H_3_-13, H_3_-14	C-14 C-1,5
4	34.2 s	—	—	—	—
5	56.2 d	1.17 dd (12.9, 2.5)	H_2_-6	H*e*-6, H*a*-7, H-9, H_3_-13	C-4,6,9,10,14,15
6	25.2 t	H*a* 1.31 qd (12.9, 3.9) H*e* 1.728 br d (12.9)	H-5, H*e*-6, H_2_-7 H-5, H*a*-6, H*a*-7	H*e*-6, H*e*-7, H_3_-14, H_3_-15 H-5, H*a*-6, H_2_-7, H_3_-13	
7	38.8 t	H*a* 2.11 td (12.9, 4.8) H*e* 2.36 ddd (12.9, 3.9, 2.5)	H_2_-6, H*e*-7, Ha-12 H_2_-6, H*a*-7	H-5, H*e*-6, H*e*-7 H_2_-6, H*a*-7, Hb-12	C-8,12 C-5,9,12
8	150.1 s	—	—	—	—
9	50.2 d	2.03–1.95 AB type	H_2_-11, H_2_-12	H*a*-1, H-5, H-5′	C-8,10,11,6′
10	41.0 s	—	—	—	—
11	22.2 t	Ha 2.20 dd (14.8, 4.6) Hb 2.03–1.95 AB type	H-9, Hb-11, Ha-12 H-9, Ha-11, Hb-12	H*e*-1, Hb-11, H_3_-15, H-5′ Ha-11, Ha-12, H_3_-15, H-5′	C-8,9,10,1′,5′,6′ C-8,9,10
12	108.0 t	Ha 4.90 br s Hb 4.79 br s	H*a*-7, H-9, Ha-11, Hb-12 H-9, Hb-11, Ha-12	Hb-11, Hb-12, H_3_-15, H-5′ H*e*-7, Ha-12	C-7,8,9 C-7,9
13	33.8 q	0.84 3H, s	H_3_-14	H_2_-3, H-5, H*e*-6	C-3,4,5,14
14	22.0 q	0.78 3H, s	H_3_-13	H*a*-2, H*e*-3, H*a*-6, H_3_-15	C-3,4,5,13
15	15.1 q	0.70 3H, s		H*e*-1, H*a*-2, H*a*-6, H_2_-11, Ha-12, H_3_-14	C-1,5,9,10
1′	192.3 s	—	—	—	—
2′	120.4 d	6.12 br s	H_2_-7′	H_2_-7′	C-3′,4′,6′,7′
3′	164.5 s	—	—	—	—
4′	71.8 s	—	—	—	—
5′	74.7 d	3.96 s	HO-5′	H-9, Hb-11, Ha-12, H*a*-8′	C-11,1′,3′,4′,6′,8′,7′
6′	85.2 s	—	—	—	—
7′	60.7 t	4.41 2H, br s	H-2′, HO-7′	H-2′, H*e*-8′	C-2′,3′
8′	43.3 t	H*a* 2.91 d (17.2)H*e* 3.05 d (17.2)	H*e*-8′ H*a*-8′	H-5′H_2_-7′	C-3′,4′,5′,9′ C-3′,4′,5′,9′
9′	167.8 s	—	—	—	—
4′-OH	—	4.90 br s			
5′-OH	—	4.73 br s	H-5′		
7′-OH	—	4.34 br s	H_2_-7′		C-7′

^a^ The ^1^H and ^13^C NMR signals were assigned on the basis of DEPT, ^1^H–^1^H COSY, HMQC, HMBC, NOESY, and 1D difference NOE experiments; ^b^ Chemical shift values (δ_H_ and δ_C_) were recorded using the solvent signals (acetone-*d*_6_: δ_H_ 2.05/δ_C_ 29.83, 206.26) as references, respectively; H*a*: axial proton; H*e*: equatorial proton; ^c^ Multiplicities of the carbon signals were determined by DEPT experiments and are indicated as s (singlet), d (doublet), t (triplet) and q (quartet), respectively; ^d^ The numbers in each line of this column indicate the protons that correlated with the proton in the corresponding line in ^1^H–^1^H COSY; ^e^ The numbers in each line of this column indicate the protons that showed NOE correlations with the proton in the corresponding line in NOESY or 1D difference NOE experiments. The NOEs between two protons in a spin coupling relationship were detected by the 1D difference NOE experiments; ^f^ The numbers in each line of this column indicate the carbons that showed HMBC correlations with the proton in the corresponding line in HMBC experiments optimized for the 8.3 Hz of long-range *J*_CH_ value; ^g^ Minus NOEs were detected on the protons H*a*-2 and H*a*-1 in the difference NOE experiments by irradiations at the protons H*a*-1 and H*a*-2, respectively.

**Figure 2:**
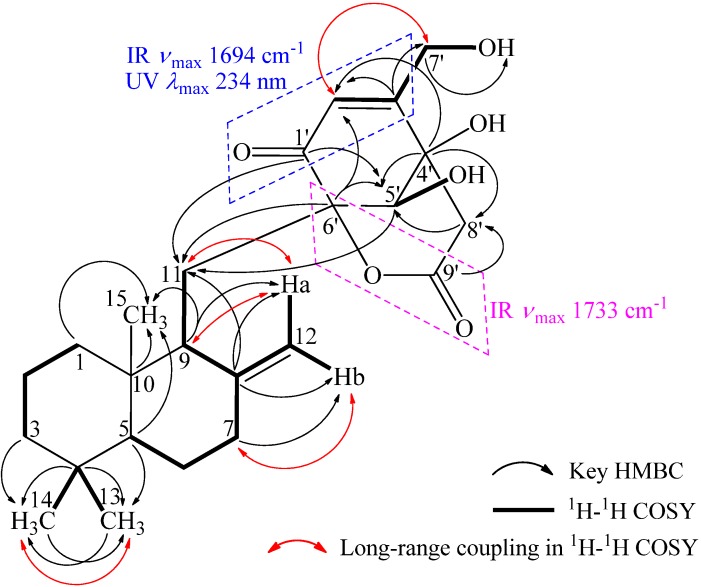
Planar structure of **1** and selected UV, IR, ^1^H–^1^H COSYand HMBC data.

Interpretation of the ^1^H–^1^H COSY data coupled with HMQC established five spin systems, C-1→C-3, C-13–viaC-4–C-14, C-5→C-7–viaC-8(C-8–C-12)–C-9–C-11, C-5′–OH, and C-2′–viaC-3′–C-7′–OH. The last one could be expanded to a conjugated enone moiety, C-1′–C-2′–C-3′–C-7′–OH, by the above mentioned information from UV and IR absorptions (Figure 2). The C-8′ methylene was neighbored to the ester carbonyl C-9′ according to the chemical shifts of both its protons and carbon (δ_H_ 2.91 and 3.05; δ_C_ 43.3). These structural moieties were further supported by the relevant HMBC correlations shown in Figure 2. At this stage, a *tert*-methyl group (CH_3_-15), one sp^3^ (C-10) and two oxygenated sp^2^ quaternary (C-4′ and C-6′) carbons, and a hydroxyl group were remaining. The connectivity of these structural fragments was established on the basis of key HMBC correlations illustrated in Figure 2, which was also supported by the other related HMBC correlations (Table 1). Then, the ester carbonyl C-9′ (δ_C_ 167.8) could be linked to C-6′ to form an ester linkage forming a six-membered ring according to the IR absorption at 1733 cm^−1^. Thus the remaining hydroxyl group was reasonably linked to C-4′, leading to the planar structure of **1** (Figure 2).

**Figure 3:**
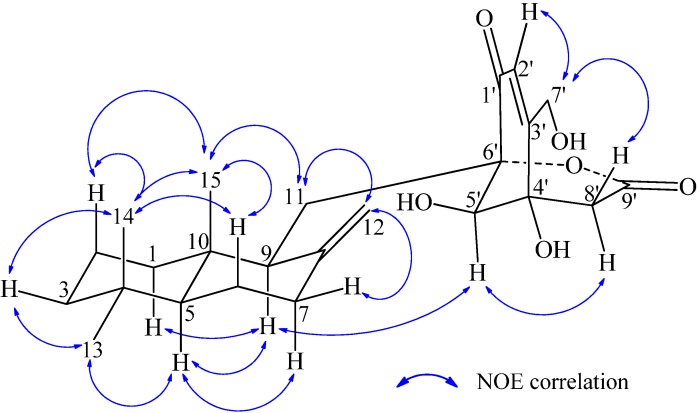
Relative stereochemistry of**1** and selected NOE correlations.

The relative stereochemistry (Figure 3) of **1** could be elucidated by NOEs and *J* values of relevant protons (Table 1). NOEs on H_3_-15/H_3_-14 and H_3_-13/H-5 indicated the *trans*-ring junction of two rings in drimene skeleton and chair-chair conformation of the two rings with an equatorial C-11 at C-9 was established by the key NOEs as shown in Figure 3. Other NOEs related and the splitting patterns and *J* values of relevant protons (Table 1) accorded well with the conformation. NOEs on H-5′/H*a*-8′ and H_2_-7′/H*e*-8′ established the conformation of bridged cyclohexenone and lactone rings as shown in Figure 3, with which the up field chemical shift of C-9′ (δ_C_ 167.8) could be explained by the shielding effect of conjugated enone group.

**Figure 4:**
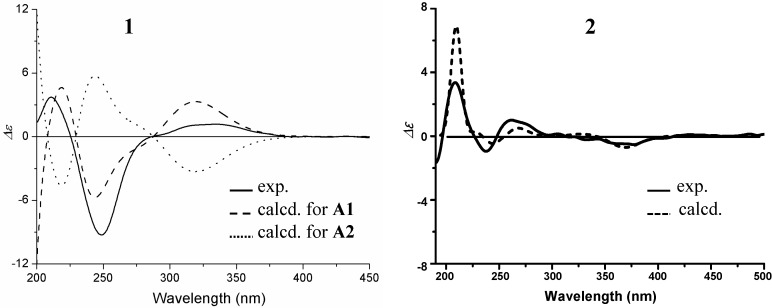
Experimentally measured CD and calculated ECD spectra of **1** and **2**.

The drimene skeleton occurs both in 5*S*9*S*10*S* [12,13,14,17,18] and 5*R*9*R*10*R* [10,11,19,20,21,22] forms in Nature. The combination of drimenyl (C-11) and cyclohexenone moiety (C-6′) affords four possible stereoisomers for **1**, as two pairs of enantiomers with absolute configurations, 5*S*9*S*10*S*4′*R*5′*R*6′*S* (**A1**) and 5*R*9*R*10*R*4′*S*5′*S*6′*R* (**A2**), 5*S*9*S*10*S*4′*S*5′*S*6′*R*(**B1**) and 5*R*9*R*10*R*4′*R*5′*R*6′*S*(**B2**), respectively. Quantum chemical TDDFT [46] calculations were performed on all four stereoisomers to obtain their ECD spectra using the software package Gaussian 09 [47]. In the theoretical calculation, **A2** and **B1** gave ECD curves opposite to the experimental CD spectrum as shown for **A2** in Figure 4 (see Supplementary Data S2 for **B1**). Thus, **A2** and **B1** were left out of consideration. Although the ECD spectrum of **A1** best matched the experimental one (Figure 4), the absolute configuration of **1** was still insufficiently defined because **B2** also reproduced properly the CD spectrum of **1** (Supplementary Data S2). **A1** and **B2** are diastereoisomers with an opposite drimenyl group and quite different specific optical rotations could be readily expected. We therefore calculated the specific optical rotations of **A1** and **B2** at the B3LYP/6-31G(d) level using B3LYP/6-31+G(d) geometries. This has afforded the calculated 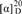 +15.1 for **A1** and 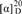 +42.8 for **B2**, respectively. The calculated 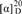 value of **A1** much better matched the measured 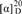 +21.0 (1.0, MeOH) of **1**. Thus, the absolute configuration of **1** was assigned as 5*S*9*S*10*S*4′*R*5′*R*6′*S*.

**Figure 5:**
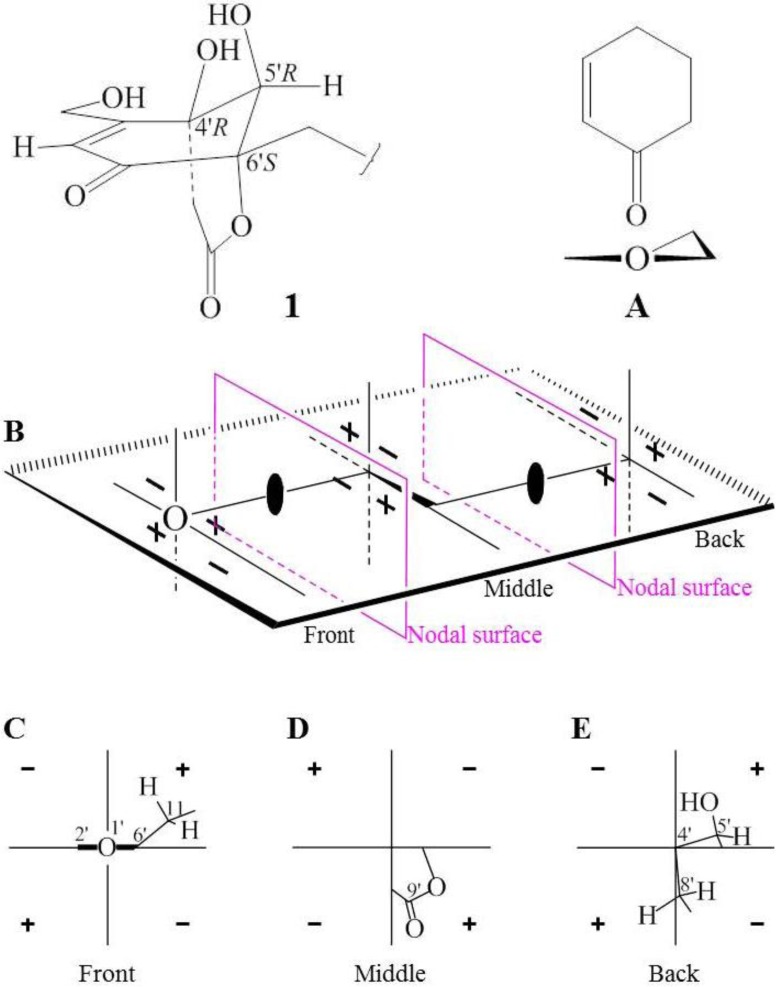
Stereo view of the cyclohexenone moiety and the prediction of CD signs for**1**. (**A**) A chirality of the second sphere for the conjugated cyclohexenone with a planar C=C–C=O chromophore. The depicted absolute configuration**A **gives rise to a positive CD for the n–π* (>300 nm) transition [48]; (**B**) Sectors for the n–π* (>300 nm) transition of planar C=C–C=O chromophore and the CD signs of ligand contributions in each sector [48]. Colored rectangular planes with a black ellipse indicate two nodal surfaces, by which the front, middle and back sector regions are divided; (**C**–**E**) Octant-like projection for**1**on the plane that parallels the nodal surfaces in (**B**), which shows the ligands that located in the front (**C**), middle (**D**) and back (**E**) sectors (showing only perturbing atoms), respectively. Signs of the ligand contributions are given for each sector.

Further evidence also supported the absolute configuration of **1**. First, the co-occurrence of **1** and **3** supported the same absolute stereochemistry of drimenyl in **1** and **3** from biogenetic considerations. Secondly, predicted CD signs of C=C–C=O for **1** by qualitative MO theory [48] and empirical CD rules [49,50] supported the absolute configuration of cyclohexenone moiety in **1**. The CD spectrum of **1** in MeOH showed positive n–π* (335.5 nm, Δε +1.16) and negative π–π* (249 nm, Δε −9.27) Cotton effects of the α,β-unsaturated ketone moiety. This opposite sign pattern was consistent with the general observations in conjugated cyclohexenones [49,50]. The rigid bicyclic system in **1** fixed the cyclohexenone ring in a sofa conformation with planar C=C–C=O (Figure 5). The helicity rule for the inherently dissymmetric C=C–C=O chromophore [49,50] therefore could not be applied to the n–π* (>300 nm) and π–π* (230–260 nm) transitions of **1**. Fortunately, a chiral second sphere of conjugated cyclohexenone with planar C=C–C=O showed that absolute configuration **A** (Figure 5) giving rise to a positive CD for the n–π* transition [48]. The same absolute configuration **A** in **1** (Figure 5) was reflected well by its positive n–π* Cotton effect. The positive CD sign of n–π* transition was also supported by careful examination of CD contributions from each ligand around C=C–C=O in **1** (**C**–**E** in Figure 5) according to the sectors (**B** in Figure 5) of qualitative MO theory [48]. The structural skeleton with a lactone bridge across the cyclohexenone ring in **1** resulted in the localization of bridged lactone ring in the middle sector region. Signs of the CD contributions are just the opposite in middle and back sectors. Thus, the simple octant rule for the planar C=C–C=O compounds could not be applied to **1**, but the sectors in Figure 5 fitted well the case. In addition, the CD spectrum of **1** gave a positive Cotton effect at 210 nm (+3.72). The sign of this Cotton effect, due to the positive “axial chirality” contribution [49,50] of 4′-CH_2_ and 6′-O groups, reflected also the structural feature of cyclohexene moiety in **1** (Figure 5).

A literature survey showed that purpurogemutantin (**1**) is a new drimene meroterpenoid structurally novel in carrying a bridged bicyclic system composed of a conjugated cyclohexenone and a six-membered lactone rings. To our best knowledge, **1** is the first drimenyl cyclohexenone derivative with the mentioned bridged bicyclic system. 

Purpurogemutantidin (**2**) was obtained as a colorless oil from MeOH and showed 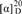 −13.7 (*c* 0.1, CHCl_3_). The elemental composition of **2**, C_22_H_32_O_4_ (7 double bond equivalents), was established by HRESIMS (*m/z*361.2377 [M + H]^+^; Δ = +0.2 mmu). The UV and IR absorptions suggested the presence of hydroxyl (IR ν_max_ 3392 cm^−1^) and ene(di)one (UV λ_max_ 236 nm, log ε 4.00; IR ν_max_ 1682 cm^−1^) [28] groups in **2**. Its ^1^H and ^13^C NMR data in CDCl_3_ (Table 2), analyzed with the aid of DEPT, ^1^H–^1^H COSY, HMQC and HMBC, indicated the characteristic signals of drimene residue seen both in macrophorins [27,28] and purpurogemutantin (**1**). The same chair-chair conformation of the drimenyl residue in **2** as that in **1** (Figure 3) was established by NOESY and difference NOE experiments: Key NOEs were detected between protons, H*a*-1/H-9, H*e*-1/H*a*-2, H*e*-1/H_3_-15, H*a*-2/H_3_-14, H*a*-2/H_3_-15, H*e*-3/H_3_-13, H*e*-3/H_3_-14, H*a*-3/H_3_-13, H-5/H*e*-6, H-5/H*a*-7, H-5/H-9, H-5/H_3_-13, H*a*-6/H*e*-7, H*a*-6/H_3_-14, H*a*-6/H_3_-15, H*e*-7/Ha-12, H_2_-11/Hb-12, H_2_-11/H_3_-15, and H_3_-14/H_3_-15, respectively. The other NOEs and the splitting patterns and *J* values of relevant protons (Table 2) also supported the same conformation. Then, further detailed analysis of the ^1^H–^1^H COSY, HMQC and HMBC data (Table 2) demonstrated the presence of a cyclo-2-hexene-1,4-dione derived moiety attached to C-11 in **2**: HMBC correlations were observed from H_2_-11 to C-8, C-9, C-10, C-1′ and C-6′; from H-2′ to C-3′, C-4′, C-6′ and C-7′; from H_2_-7′ to C-2′ and C-3′; from Ha-5′ to C-11, C-1′, C-3′, C-4′ and C-6′; from Hb-5′ to C-11, C-1′, C-4′ and C-6′; and from the 6′-OH proton to C-1′, C-5′ and C-6′. A remaining hydroxyl group was thus reasonably located at C-7′ of **2**. The ESIMS, HRESIMS, UV, IR, ^1^H and ^13^C NMR, DEPT, ^1^H–^1^H COSY, HMQC, HMBC, and NOESY spectra of **2** are provided as supporting information in Supplementary Data S3.

**Table 2:** 400 MHz ^1^H and 100 MHz ^13^C NMR data of **2** in CDCl_3_^a^.

Position	δ_C_^b,c^	δ_H_^b^ (*J* in Hz)	COSY ^d^	NOE ^e^	HMBC ^f^
1	38.7 t	H*a* 1.07 td (12.6, 4.8) H*e* 1.63 br d (12.6)	H*e*-1, H_2_-2 H*a*-1, H*a*-2	H*e*-1, H-9 H*a*-1, H*a*-2, H_3_-15	C-3,5
2	19.3 t	1.56–1.45 2H, AB type m H*a* at lower field H*e* at higher field	H_2_-1, H_2_-3 H_2_-1, H_2_-3	H_3_-14, H_3_-15 H*a*-1	C-4,10
3	42.0 t	H*a* 1.18 td (12.4, 4.8) H*e* 1.37 br d (12.4)	H_2_-2, H*e*-3 H_2_-2, H*a*-3	H*e*-3, H_3_-13 H*a*-3, H_3_-13, H_3_-14	C-14 C-1,5,14
4	33.7 s	—	—	—	—
5	55.6 d	1.13 dd (12.8, 2.4)	H_2_-6	H*e*-6, H*a*-7, H-9, H_3_-13	C-6,9,10,14,15
6	24.6 t	H*a* 1.25 qd (12.8, 3.8 )H*e* 1.74 dm (12.8)	H-5, H*e*-6, H_2_-7 H-5, H*a*-6, H_2_-7	H*e*-6, H*e*-7, H_3_-14, H_3_-15 H-5, H*a*-6, H_3_-13	C-5,7
7	38.1 t	H*a* 1.89 td (12.8, 5.2) H*e* 2.28 ddd (12.8, 3.8, 2.4)	H_2_-6, H*e*-7 H_2_-6, H*a*-7	H-5, H*e*-6, H*e*-7 H*a*-6, H*a*-7, Ha-12	C-8,12 C-5,6,8,9,12
8	148.9 s	—	—	—	—
9	50.5 d	1.77 t (4.7)	H_2_-11	H*a*-1, H-5	C-8,10,6′
10	39.9 s	—	—	—	—
11	34.8 t	1.88 2H, d (4.7)	H-9	H*e*-1, Hb-12, H_3_-15	C-8,9,10,1′,6′
12	107.1 t	Ha 4.75 br s Hb 4.25 br s	H*a*-7, Hb-12 Ha-12	H*e*-7, Hb-12 H_2_-11, Ha-12, H_3_-15	C-7,9 C-7,9
13	33.5 q	0.85 3H, s	H_3_-14	H*a*-3, H-5, H*e*-6	C-3,4,5,14
14	21.6 q	0.75 3H, s	H_3_-13	H*a*-2, H*e*-3, H*a*-6, H_3_-15	C-3,4,5,13
15	15.0 q	0.57 3H, s		H*e*-1, H*a*-2, H*a*-6, H_2_-11, Hb-12, H_3_-14	C-1,5,9,10
1′	201.2 s	—	—	—	—
2′	134.4 d	6.82 br s	H_2_-7′	—	C-3′,4′,6′,7′
3′	150.9 s	—	—	—	—
4′	196.6 s	—	—	—	—
5′	53.1 t	Ha 3.12 d (16.0) Hb 2.97 d (16.0)	Hb-5′ Ha-5′	Hb-5′, H_2_-11 Ha-5′	C-11,1′,3′,4′,6′ C-11,1′,4′,6′
6′	77.4 s	—	—	—	—
7′	59.6 t	Ha 4.54 br d (17.2) Hb 4.44 br d (17.2)	H-2′, Hb-7′ H-2′, Ha-7′	Hb-7′ Ha-7′	C-2′,3′ C-2′,3′
6′-OH	—	3.87 br s	—		C-1′,5′,6′
7′-OH	—	3.48 br s	—		

^a^ The ^1^H and ^13^C NMR signals were assigned on the basis of DEPT, ^1^H–^1^H COSY, HMQC, HMBC, NOESY, and 1D difference NOE experiments; ^b^ Chemical shift values (δ_H_ and δ_C_) were recorded using the solvent signals (CDCl_3_: δ_H_7.26/δ_C_77.1) as references, respectively; H*a*: axial proton; H*e*: equatorial proton; ^c^ Multiplicities of the carbon signals were determined by DEPT experiments and are shown as s (singlet), d (doublet), t (triplet) and q (quartet), respectively; ^d^ The numbers in each line of this column indicate the protons that correlated with the proton in the corresponding line in ^1^H–^1^H COSY; ^e^ The numbers in each line of this column indicate the protons that showed NOE correlations with the proton in the corresponding line in NOESY or 1D difference NOE experiments. The NOEs between two protons in a spin coupling relationship were detected by the 1D difference NOE experiments; ^f^ The numbers in each line of this column indicate the carbons that showed HMBC correlations with the proton in the corresponding line in the HMBC experiments optimized for the 8.3 Hz of long-range *J*_CH_ value.

There are also four possible stereoisomers belonging to two pairs of enantiomers, with the absolute configurations 5*S*9*S*10*S*6′*S*, 5*S*9*S*10*S*6′*R*, 5*R*9*R*10*R*6′*S* and 5*R*9*R*10*R*6′*R*, by connecting the drimenyl group (C-11) to the cyclohexenedione moiety (C-6′) for **2**. In ECD calculations on all four possible stereoisomers with respect to the drimene skeleton and C-6′, only one result matched the experimental CD spectrum of **2** (Figure 4) well, leading to the absolute configuration of **2** as 5*S*9*S*10*S*6′*S* (Figure 1). Calculated ECD spectra of all four questionable stereoisomers and their comparisons with the experimental CD spectrum of **2** are given as supporting information in Supplementary Data S2.

Recently, Lin *et al*. reported a new drimene meroterpenoid with the same structure as **2** and named it penicilliumin A [31]. The authors assigned the relative stereochemistry of the drimenyl in penicilliumin A, being the same as that in **2**, but did not determine its full absolute configuration. The ^13^C NMR data of **2** are coincident with those of penicilliumin A [31], however, the two compounds had a quite different appearance and specific optical rotation: Compound **2**, a colorless oil (MeOH), 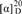 −13.7 (*c* 0.1, CHCl_3_), 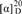 −9.3 (*c* 0.5, MeOH); penicilliumin A, white crystalline solid, 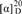 −0.008 (*c* 0.85, CHCl_3_). In view of the presence of four possible stereoisomers together with the incompletely defined structure of penicilliumin A, we designated purpurogemutantidin (**2**) as a new compound.

**Figure 6:**
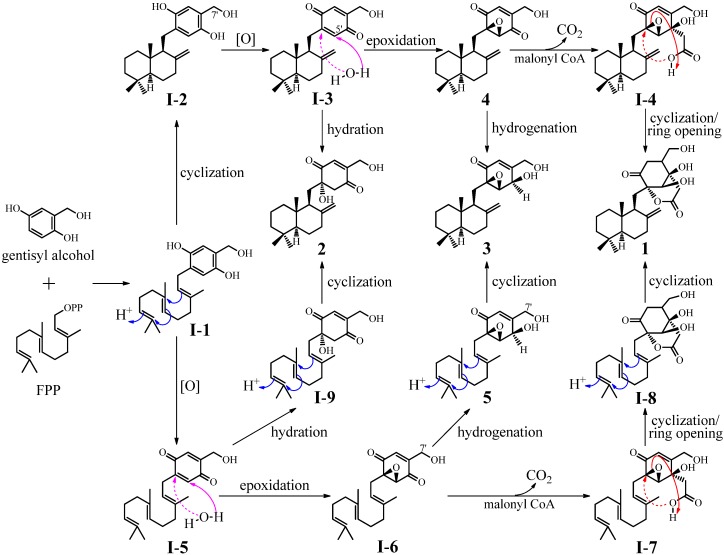
Plausible biosynthetic pathways of **1**–**3**.

Plausible biosynthetic pathways for **1**–**3** are proposed in Figure 6. A co-intermediate of the **1**–**3** biosynthesis is proposed to be **I**-**1**, which might be produced by enzymatic reaction of gentisyl alcohol and farnesyl pyrophosphate (FPP), a biosynthetic precursor of all sesquiterpenoids [51]. Gentisyl alcohol has been isolated from fungal sources and is known to be biosynthesized via the polyketide pathway [52,53]. A straightforward cyclization of the farnesyl group in **I**-**1**, followed by oxidation of gentisyl phenols and then epoxidation on the formed *para*-quinone ring, would give intermediates **I**-**2**, **I**-**3**, and **4**. The C-7′ carbaldehyde form of **I**-**2** [16], 5′-hydroxy **I**-**3** (tauranin) [12,13,17] and macrophorin A (**4**) [24] have been isolated from fungal metabolites. The hydration of **I**-**3** and hydrogenation of **4** would produce compounds **2** and **3**. On the other hand, malonylation of macrophorin A (**4**) followed by decarboxylation would give the intermediate **I**-**4**. Then, cyclization of **I**-**4** accompanied with the epoxy ring opening would give compound **1**. Another possibility is that the intermediate **I**-**1 **underwent chemical modifications on the gentisyl alcohol moiety at first to produce final intermediates **I**-**9**, **5** and **I**-**8** via the intermediates **I**-**5**, **I**-**6** and **I**-**7** (Figure 6). Then, cyclization of the farnesyl groups in **I**-**8**, **I**-**9** and **5**would also afford compounds **1**, **2** and **3**, respectively. This possibility was supported by isolation of **5** (22-deacetylyanuthone A), 7′-acetyl **5** (yanuthone A), and 7′-acetyl **I**-**6** (yanuthone B) from a marine-derived fungus [54].

### 2.2. Inhibitory Effects of *1–3* on Several Human Cancer Cell Lines

Both new compounds **1** and **2** significantly inhibited the human chronic leukemia K562, acute leukemia HL-60, cervical cancer HeLa, gastric adenocarcinoma BGC-823 and breast cancer MCF-7 cells, with the inhibition rates (IR%) ranging from 62.8%–88.0% at 100 µg/mL in the MTT assay (Table 3). Docetaxol and 5-fluorouracil as positive controls showed similar inhibition at the same concentration. The half inhibitory concentrations (IC_50_, µM) of **1** and **2** on the K562, HL-60, HeLa, BGC-823 and MCF-7 cells were determined by the MTT method and are given in Table 4.

**Table 3:** Inhibitory effects of **1** and **2** on human cancer cell lines tested by MTT assay.

Sample	IR% Value at the 100 µg/mL Test Sample				
	K562	HL-60	HeLa	BGC-823	MCF-7
**1**	62.8%	74.5%	88.0%	87.3%	86.5%
**2**	71.3%	70.9%	83.1%	81.3%	77.7%
docetaxol	71.2%	74.9%	84.4%	70.2%	69.7%
5-fluorouracil	57.6%	66.3%	78.4%	69.1%	66.6%

The cells were treated with the sample at 100 µg/mL for 48 h, and the inhibitory effect was assayed by the MTT method. Docetaxol and 5-fluorouracil were used as positive control.

**Table 4:** The IC_50_ (µM) of **1** and **2** on human cancer cell lines by MTT assay.

Compound	K562	HL-60	HeLa	BGC-823	MCF-7
**1**	13.4	18.1	18.9	33.0	29.3
**2**	0.93	2.48	16.6	31.0	26.3

The cells were treated with the compound at series concentrations for 48 h, and the half inhibitory concentration (IC_50_) of the compound was determine by the MTT assay.

The inhibitory effect of the known compound macrophorin A (**3**) was also assayed using the K562 and HL-60 cells by the MTT method. Compound **3** significantly inhibited the growth of K562 and HL-60 cells with IC_50_ values of 1.48 and 0.85 µM, respectively. This is the first record of its effect on human cancer cells, confirming the original finding of its cytotoxicity on murine L-5178Y cells [24].

The ethyl acetate extract of parent strain G59 did not show any inhibitory effect on the K562 cells at 1000 or 100 µg*/*mL [37]. An IR% value of 5.4% at the 100 µg*/*mL was also detected for the G59 extract from its cultures fermented at the same time and same conditions with the mutant BD-1-6 in present study. Thus, the above mentioned structural and biological results on **1**–**3** revealed that **1**–**3** should be newly produced by the mutant BD-1-6 compared to its parent strain G59. This was further supported by the followed HPLC and LC-ESIMS analysis of the EtOAc extracts both from the parent G59 and its mutant BD-1-6.

### 2.3. Experimental Verification of the Absence of *1–3* in the G59 Products by HPLC and LC-ESIMS

The ethyl acetate extracts from G59 and its mutant BD-1-6 cultured at the same time and in the same conditions were subjected to HPLC and LC-ESIMS analysis under the same LC conditions using **1**–**3** as control.

**Figure 7:**
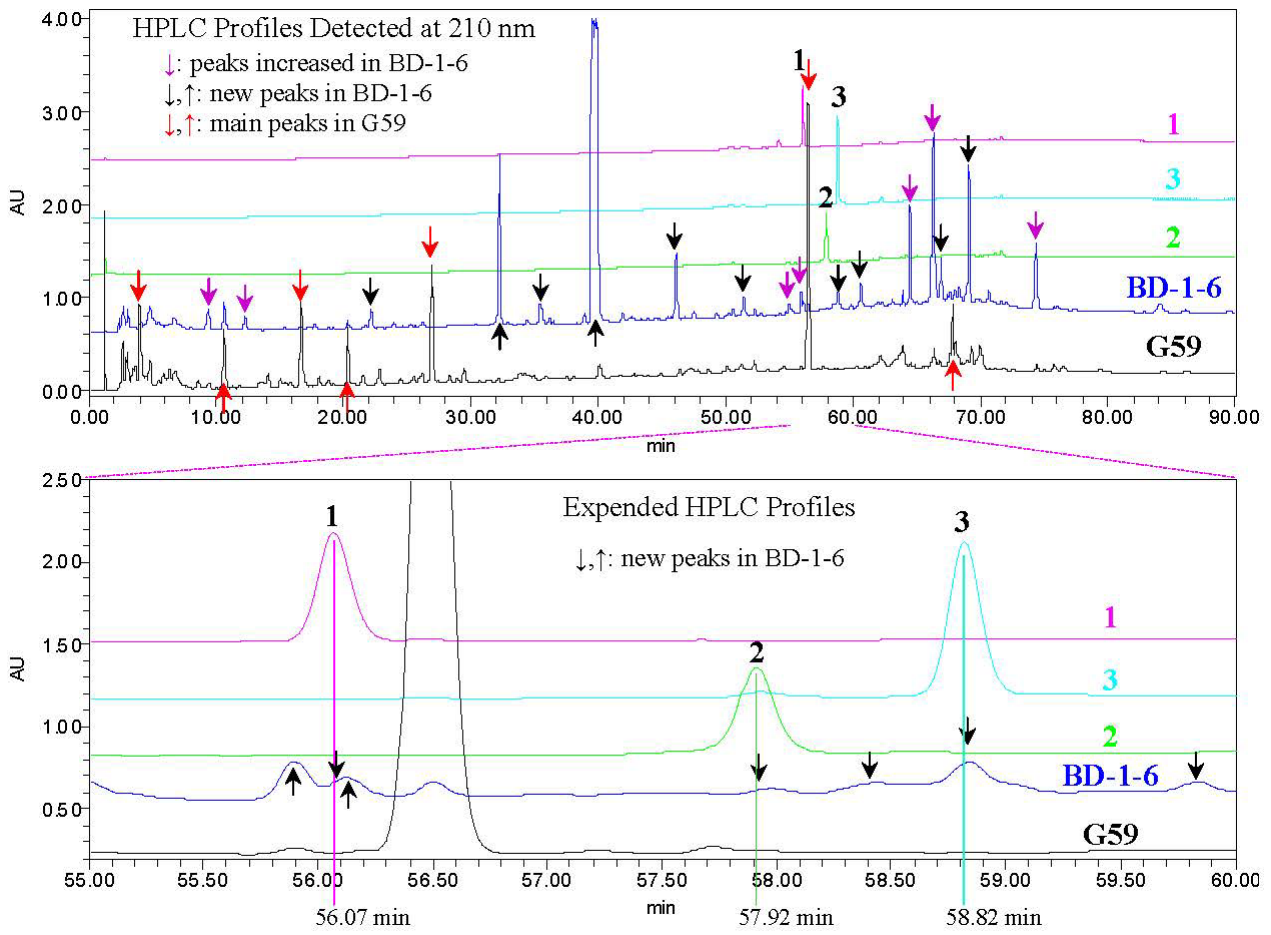
HPLC analysis of the compounds **1**–**3** and the BD-1-6 and G59 extracts. EtOAc extracts of both the mutant BD-1-6 and its parent G59 cultures fermented at the same time and in the same conditions were used for the HPLC analysis, and the same sample amounts were injected into a column and analyzed by the HPLC inthe same conditions.

In the HPLC analysis, **1**–**3** were eluted as peaks with retention times (*t*_R_) of 56.07 min (**1**), 57.92 min (**2**) and 58.82 min (**3**), respectively, and detected all as minor metabolites in the mutant BD-1-6 but not in the parent G59 (Figure 7 and Supplementary Data S4). The detection of **1**–**3** in BD-1-6 and their absence in G59 were also verified by the UV curves of both BD-1-6 and G59 at the same retention times (see details in Supplementary Data S4).

In the LC-ESIMS analysis, ion peaks of **1**–**3** appeared with shortened retention times (*t*_R_: 52.3 min for **1**, 53.8 min for **2**, and 55.1 min for **3**) than those in HPLC (Figure 7) because of the shortened flow length from the outlet of the HPLC column to the inlet of MS in LC-ESIMS. Examination of **1**–**3** in G59 and BD-1-6 were performed using selective ion ([M + Na]^+^ for **1**–**3** and [M − H]^−^ for **1**) monitoring with both chromatograms and related MS spectra. In the LC-ESIMS analysis, **1**–**3** were also detected only in BD-1-6 but entirely not in G59 (Supplementary Data S5), further confirming the above results of HPLC analysis.

All of the above mentioned results and discussions indicated that DES-induced mutation(s) in the mutant BD-1-6 activated some silent biosynthetic pathways in the parent strain G59, including one set of those for the **1**–**3** production (Figure 6), although detailed biological mechanisms remain unknown. Nevertheless, the discovery of **1** and **2** from BD-1-6 revealed that DES mutagenesis of the biosynthetically inactive fungal strains to produce bioactive metabolites is likely worth further investigation to search for new bioactive compounds by altering their secondary metabolisms.

## 3. Experimental Section

### 3.1. General Experimental

The melting point was measured on a Beijing Tiandiyu X-4 exact micro melting point apparatus (uncorrected). Optical rotations were measured on a Rudolph Research Autopol II spectropolarimeter. ESIMS was recorded on an Applied Biosystems API 3000 LC-MS spectrometer. HRESIMS was measured on an Agilent 6520 Q-TOF LC-MS spectrometer. UV data were recorded on a GBC Cintra 20 spectrophotometer, and IR spectra were taken on a Bruker Tensor-27 infrared spectrophotometer. CD data were recorded on a Biologic Science MOS 450 CD or a JASCO J-815 spectropolarimeter. 1D and 2D NMR spectra were obtained on a JEOL JNM-GX 400 (400 MHz ^1^H and 100 MHz ^13^C NMR) NMR spectrometer using the solvent signals (CDCl_3_: δ_H_ 7.26/δ_C_77.1; acetone-*d*_6_: δ_H_ 2.05/δ_C_ 29.83, 206.26; CD_3_OD: δ_H_3.31/δ_C_49.0) as references, respectively.

Precoated silica gel GF_254_plates (Yantai Chemical Industrial Institute, Yantai, China) were used in TLC and spots were detected under UV light (254 and 365 nm) or by using Vaughan’s reagent [44] or 10% sulfuric acid reagent. Silica gel (100–200 mesh, Yantai Chemical Industrial Institute, Yantai, China), YMC_*_GEL^®^ ODS-A-HG (12 nm S-50 µM, YMC Co. Ltd., Kyoto, Japan), and Sephadex™ LH-20 (GE Healthcare, Uppsala, Sweden) were used for column chromatography. Semi-preparative HPLC was performed using a Senshu Pak ODS 3251-D column (8 × 250 mm; Senshu Scientific Co. Ltd., Tokyo, Japan) on a Waters HPLC equipment using Waters 600 controller, Waters 600 pump, Waters 2414 refractive index detector, Waters 2996 photodiode array detector and Waters Empower™ software.

Electronic circular dichroism (ECD) spectra for each of four stereoisomers of **1** and**2** were obtained by quantum chemical time-dependent density functional theory (TDDFT) [46] calculations using the software package Gaussian 09 [47]. Conformational searches were performed by using the MMFF94S molecular mechanics force field and charged with MMFF94. The resulting conformation were optimized by DFT at the B3LYP/6-31G level. The theoretical ECD calculations on the optimized conformers were performed by using the TDDFT method at B3LYP/6-311++G(2d,p) level. The polarizable continuum model (PCM) was adopted to consider solvent effects using the dielectric constant of methanol (ε = 32.6). The specific optical rotations of two diasteroisomers **A1** and **B2** for **1** were calculated at the B3LYP/6-31G(d) level using B3LYP/6-31+G(d) geometries.

### 3.2. Fungal Strain and Human Cancer Cell Line

*P. purpurogenum* G59 is a marine-derived wild-type fungal strain G59, which was isolated from a soil sample collected at the tideland of Bohai Bay around Lüjühe in Tianjin, China, in September 2004 [37] and identified by L. Guo, Institute of Microbiology, Chinese Academy of Sciences, Beijing, China. This strain was deposited at the China General Microbiological Culture Collection Center under the accession number CGMCC No. 3560. The strain G59 did not produce secondary metabolites showing antitumor effect on K562 cells, indicated by the inhibition rate (IR%, lower than 6.9% at 1000 µg/mL in every test) of its EtOAc extract [37,44] as also reconfirmed in the present study.

The producing strain *P. purpurogenum* BD-1-6 is a bioactive mutant obtained by random diethyl sulfate (DES) mutagenesis of the strain G59. The G59 spores in 20% (v/v) DMSO containing 0.5% (v/v) DES were stored at 4 °C for 1 day to treat the spores with DES. Then, single colony isolation was carried out using the treated G59 spores to obtain the mutant BD-1-6. The mutant strain BD-1-6 was deposited in the China General Microbiological Culture Collection Center under the accession number CGMCC No. 5525.

In the MTT assay for screening of bioactive mutants, the EtOAc extract of BD-1-6 culture significantly inhibited K562 cells (IR%, 60.1% at 100 µg/mL), whereas the EtOAc extract of G59 culture fermented at the same time and in the same conditions in present study did not show any inhibitory effect on K562 cells (IR%, 4.8% at 100 µg/mL).

Human chronic myelogenous leukemia K562 cell line was provided by Song Li (Beijing Institute of Pharmacology and Toxicology). Human acute promyelocytic leukemia HL-60, human cervical cancer HeLa, Human gastric adenocarcinoma BGC-823 and human breast cancer MCF-7 cell lines were provide by Wenxia Zhou (Beijing Institute of Pharmacology and Toxicology). The cells were routinely maintained at 37 °C in RPMI-1640 medium supplemented with 10% (v/v) fetal bovine serum in the presence of 100 µg*/*mL penicillin and streptomycin under a humidified atmosphere of 5% CO_2_ and 95% air.

### 3.3. Fermentation and EtOAc Extract Preparation

Fresh spores of the mutant BD-1-6 were inoculated into a 500 mL Erlenmeyer flask containing 200 mL of liquid medium (glucose 2%, maltose 1%, mannitol 2%, glutamic acid 1%, peptone 0.5% and yeast extract 0.3% in distilled water, adjusted to pH 6.0 prior to sterilization) and cultured at 28 °C for 48 h on a rotary shaker at 200 rpm. Each 10 mL of this culture broth was inoculated into 7 of 500 mL Erlenmeyer flasks containing 200 mL of the same liquid medium and further cultured under the same condition for 48 h to obtain a seed culture (1400 mL). Each 10 mL of the seed culture was inoculated into 100 of 500 mL Erlenmeyer flasks with 200 mL of the same liquid medium. Then, the producing fermentation was performed on rotary shaker at 200 rpm at 28 °C for 12 days. The whole broth (20 L) was separated into a filtrate and a mycelial cake. The mycelial cake was extracted three times with 80% (v/v) aqueous acetone (10 L) by ultrasonication for 2 h. The aqueous acetone solution obtained by filtration was evaporated under reduced pressure to remove acetone. The remaining water layer (6 L) was extracted three times with equal volumes of EtOAc to give an extract (17 g). The EtOAc extracts showed an inhibitory effect on K562 cells with an IR% value of 58.6% at 100 µg*/*mL.

The original strain G59 was also fermented in the same manner as that for mutant BD-1-6 at the same time and same conditions using 3 of 500 mL Erlenmeyer flasks with 200 mL of the same liquid medium. Extraction of the whole broth (600 mL) as describe above for mutant BD-1-6 provided an EtOAC extract (610 mg) , which did not show any inhibitory effect on K562 cells (an IR% value of 5.4% at 100 µg*/*mL). This extract was used for assay and HPLC and LC-ESIMS analysis.

### 3.4. Isolation of Compounds *1–3*

The EtOAc extract (17 g) of mutant BD-1-6 was subjected to silica gel column (silica gel 300 g, bed 4.5 × 40 cm) chromatography by stepwise elution with dichloromethane (D)–acetone (A) (100:0→0:100) to obtain 6 fractions: **Fr**-**1** (4.6 g, eluted by dichloromethane), **Fr**-**2** (2.6 g, eluted by dichloromethane), **Fr**-**3** (1.1 g, eluted by D–A 95:5), **Fr**-**4** (3.1 g, eluted by D–A 95:5), **Fr**-**5** (1.7 g, eluted by D–A 95:5→80:20), and **Fr**-**6** (2 g, eluted by acetone). **Fr**-**3** and **Fr**-**5** showed inhibitory effect on K562 cells with the IR% values of 56.9 and 45.2% at the 100 μg/mL, respectively.

**Fr**-**3** (1.1 g) was subjected again to a silica gel column (silica gel 25 g, bed 1.5 × 35 cm) and a stepwise elution with cyclohexane (C)–acetone (A) (100:0→60:40) giving 3 fractions: **Fr**-**3**-**1** (480 mg, eluted by cyclohexane), **Fr**-**3**-**2** (121 mg, eluted by C–A 95:5→85:15), and **Fr**-**3**-**3** (310 mg, eluted by C–A 80:20→75:25→60:40). **Fr**-**3**-**2** inhibited the K562 cells with an IR% value of 62.1% at the 100 μg/mL. The whole **Fr**-**3**-**2** (121 mg) was separated by semi-preparative HPLC (Senshu Pak ODS 3251-D column, room temperature, mobile phase MeOH–H_2_O 80:20, flow rate 2.0 mL/min, detecting wave lengths 210 and 254 nm) to obtain **2** (6.2 mg, retention time *t*_R_ = 20.1 min).

**Fr**-**5** (1.7 g) was subjected to a Sephadex LH-20 column (bed 2 × 130 cm) in CH_2_Cl_2_–MeOH (1:1) and eluted with the same CH_2_Cl_2_–MeOH (1:1) to afford 4 fractions in the order of elution: **Fr**-**5**-**1** (590 mg), **Fr**-**5**-**2** (760 mg), **Fr**-**5**-**3** (113 mg), and **Fr**-**5**-**4** (230 mg). **Fr**-**5**-**2** and **Fr**-**5**-**3** inhibited the K562 cells with the IR% values of 42.4 and 50.9% at the 100 μg/mL, respectively. **Fr**-**5**-**3** (113 mg) was separated by semi-preparative HPLC (Senshu Pak ODS 3251-D column, room temperature, mobile phase MeOH–H_2_O 77:23, flow rate 2.0 mL/min, detecting wave lengths 210 and 254 nm) to obtain **1** (21 mg, *t*_R_ = 21 min).

**Fr**-**5**-**2** (760 mg) was further separated by Sephadex LH-20 column chromatography (bed 2 × 130 cm, eluting with CH_2_Cl_2_–MeOH 1:1) to give 3 fractions in the order of elution: **Fr**-**5**-**2**-**1** (51 mg), **Fr**-**5**-**2**-**2** (430 mg), and **Fr**-**5**-**2**-**3** (270 mg). **Fr**-**5**-**2**-**2** inhibited the K562 cells with an IR% value of 58.8% at the 100 μg/mL. **Fr**-**5**-**2**-**2** (430 mg) was thus subjected to a ODS column (bed 1.2 × 10 cm) and elution with MeOH–H_2_O (20:80→95:5) afforded 7 fractions: **Fr**-**5**-**2**-**2**-**1** (11 mg, eluted by MeOH–H_2_O 20:80→25:75), **Fr**-**5**-**2**-**2**-**2** (17 mg, eluted by MeOH–H_2_O 30:70), **Fr**-**5**-**2**-**2**-**3** (34 mg, eluted MeOH–H_2_O 40:60→60:40), **Fr**-**5**-**2**-**2**-**4** (107 mg, eluted by MeOH–H_2_O 80:20), **Fr**-**5**-**2**-**2**-**5** (25 mg, eluted by MeOH–H_2_O 80:20), **Fr**-**5**-**2**-**2**-**6** (14 mg, eluted by MeOH–H_2_O 80:20), and **Fr**-**5**-**2**-**2**-**7** (215 mg, eluted by MeOH–H_2_O 95:5). **Fr**-**5**-**2**-**2**-**4** inhibited the K562 cells with an IR% value of 59.3% at the 100 μg/mL. Thus, **Fr**-**5**-**2**-**2**-**4** (107 mg) was further subjected to semi-preparative HPLC separation (Senshu Pak ODS 3251-D column, room temperature, mobile phase MeOH–H_2_O 75:25, flow rate 2.0 mL/min, detecting wave lengths 210 and 254 nm) to obtain **3** (19 mg, *t*_R_ = 39.6 min).

### 3.5. MTT Assay

The EtOAc extracts both from BD-1-6 and G59, compounds **1**–**3**, 5-fluorouracil (5-FU) (Aladdin Chemistry Co. Ltd., lot No. 5402), and docetaxol (DOC) (Beijing Chimivo Technology Co. Ltd., lot No. 20110326) were dissolved in DMSO to prepare 10.0 mg/mL stock solutions, and serial dilutions of test samples were made for MTT assay. 5-FU and DOC were used as positive control, and DMSO was used as blank control.

Exponentially growing K562, HL-60, HeLa, BGC-823 and MCF-7 cells were suspended in fresh RPMI-1640 medium at the density of 2 × 10^4^ cells/mL and then seeded into 96-well plates at 200 μL/well. The suspended K562 and HL-60 cells were incubated at 37 °C for 2 h, whereas the adherent cells HeLa, BGC-823 and MCF-7 were incubated at 37 °C for 12 h. Then, 2 μL of DMSO for control and the test sample solutions was added to each well, and the cells were cultured at 37 °C for 48 h. After morphological examination of the cells under a reversed phase microscope, MTT (20 μL; 5 mg/mL in PBS) was added into each well, incubated at 37 °C for 4 h, and centrifuged at 2000 rpm for 20 min. After removal of the supernatant by aspirating, 150 μL DMSO was added into each well, and shaken for 5 min to dissolve formazan crystals. The OD value at 570 nm was read for each well using the VERSAmax-BN03152 plate reader. Each three wells were set for control and test groups, respectively, and the inhibition rate (IR%) was calculated using OD mean values according to the formula, IR% = (OD_control_ − OD_sample_)/OD_control_ × 100%. The IC_50_ value for a sample was obtained from the IR% values of the sample at different concentrations.

### 3.6. Physicochemical and Spectral Data for Compounds *1–3*

Purpurogemutantin (**1**): White crystalline powder (MeOH), mp 122–123 °C, 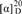 +21.0 (*c* 1.0, MeOH). UV λ_max_ nm (log ε) in MeOH: 234 (3.87). IR ν_max_ cm^−1^: 3405, 2934, 1733, 1694, 1459, 1442, 1388, 1365, 1277, 1246, 1229, 1114, 1067, 1044, 1021, 889. CD (0.48 mM, MeOH) Δε (nm): 0 (388.5), +1.16 (335.5), 0 (286.5), −9.27 (249), 0 (225.5), +3.72 (210). ^1^H and ^13^C NMR data in acetone-*d*_6_: see Table 1. Positive ESIMS *m/z*: 419 [M + H]^+^, 441 [M + Na]^+^, 837 [2M + H]+, 859 [2M + Na]+; negative ESI-MS *m/z*: 531 [M + CF_3_CO_2_]^−^. Positive HRESIMS *m/z*: measured 419.2431 [M + H]^+^, calculated for C_24_H_35_O_6_ [M + H] 419.2434; measured 436.2692 [M + NH_4_]^+^, calculated for C_24_H_3__8_NO_6_ [M + NH_4_] 436.2699; measured 441.2234 [M + Na]^+^, calculated for C_24_H_3__4_O_6_Na [M + Na] 441.2253.

Purpurogemutantidin (**2**): Colorless oil (MeOH), 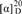 −13.7 (*c* 0.1, CHCl_3_), 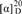 −9.3 (*c* 0.5, MeOH); (penicilliumin A, white crystalline solid, 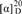 −0.008 (*c* 0.85, CHCl_3_) in literature [31]). UV λ_max_ nm (log ε) in MeOH: 236 (4.00). IR ν_max_ cm^−1^: 3392, 2941, 1682, 1457, 1443, 1388, 1368, 1243, 1208, 1092, 1036, 898, 829. CD (0.27 mM, MeOH) Δε (nm): 0 (415.5), −0.57 (360.5), 0 (320), +1.14 (260.5), 0 (247.6), −1.25 (237), 0 (226), +3.74 (209.5); CD λ_max_ nm (mdeg) in MeOH at 100 μg/mL: 415.5 (0), 360.5 (−0.52177), 320 (0), 260.5 (+1.04783), 247.6 (0), 237 (−1.14715), 226 (0), 209.5 (+3.42439). ^1^H and ^13^C NMR data in CDCl_3_: see Table 2. Positive ESIMS *m/z*: 383 [M + Na]^+^; negative ESIMS *m/z*: 341 [M − H_2_O − H]^−^, 359 [M − H]^−^, 395 [M + Cl]^−^. Positive HRESIMS *m/z*: measured 743.4486 [2M + Na]+, calculated for C_44_H_64_O_8_Na [2M + Na] 743.4499; measured 399.1939 [M + K]^+^, calculated for C_22_H_3__2_O_4_K [M + K] 399.1938; measured 383.2193 [M + Na]^+^, calculated for C_22_H_32_O_4_Na [M + Na] 383.2198; measured 361.2377 [M + H]^+^, calculated for C_22_H_3__3_O_4_ [M + H] 361.2379; measured 343.2269 [M − H_2_O + H]^+^, calculated for C_22_H_3__3_O_4_ [M − H_2_O + H] 343.2273. Negative HRESIMS *m/z*: measured 395.1995 [M + Cl]^−^, calculated for C_22_H_3__2_O_4_Cl [M + Cl] 395.1989; measured 359.2223 [M − H]^−^, calculated for C_22_H_3__1_O_4_ [M − H] 359.2222; measured 341.2118 [M − H_2_O − H]^−^, calculated for C_22_H_29_O_3_ [M − H_2_O − H] 341.2117.

Macrophorin A (**3**): a pale-colored solid (MeOH), 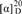 +25.7 (*c* 1.0, MeOH), ([α]_D_ +29 (MeOH) in literature [24]). Positive ESIMS *m/z*: 361 [M + H]^+^, 378 [M + NH_4_]^+^, 383 [M + Na]^+^; negative ESIMS *m/z*: 359 [M − H]^−^, 395 [M + Cl]^−^, 405 [M + HCOO]^−^. IR ν_max_ cm^−1^: 3400, 2938.6, 2867.8, 2844.7, 1677.4, 1458.9, 1439.9, 1387.8, 1365.7, 1278.9, 1204.3, 1103.6, 1030.4, 882.1, 669.1. CD (2.78 mM, MeOH) Δε (nm): 0 (389), +2.76 (334), 0 (280), −4.41 (243), 0 (218), +0.31 (215), 0 (212), −1.92 (204). CD λ_max_ nm (mdeg) in MeOH at 1.0 mg/mL: 389 (0), 334 (+25.33), 280 (0), 243 (−40.40), 218 (0), 215 (+2.80), 212 (0), 204 (−17.56). ^1^H NMR (400 MHz, CD_3_OD) δ: 5.91 (1H, d, *J* = 1.6, H-2′), 4.77 (1H, br s, Ha-12), 4.52 (2H, br s, Hb-12 and H-4′), 4.29 (1H, d, *J* = 17.7 Hz, Ha-7′), 4.21 (1H, d, *J* = 17.7 Hz, Hb-7′) , 3.67 (1H, d, *J* = 2.9 Hz, H-5′), 2.33 (1H, ddd, *J* = 12.6, 3.9, 2.5 Hz, H*e*-7), 2.31 (1H, d, *J* = 14.0 Hz, Ha-11), 1.92 (1H, td, *J* = 12.6, 3.9 Hz, H*a*-7), 1.81 (1H, dd, *J* = 14.0, 11.1 Hz, Hb-11), 1.76 (1H, dt, calcd *J* = 12.6, 3.9 Hz, H*e*-1; overlapped with H-9 and H*e*-6), 1.75 (1H, d, *J* = 11.1 Hz, H-9; overlapped with H*e*-1 and H*e*-6), 1.72 (1H, m, H*e*-6; overlapped with H*e*-1 and H-9), 1.60 (1H, qt, *J* = 13.6, 3.4 Hz, H*a*-2), 1.49 (1H, dquint, *J* = 13.6, 3.4 Hz, H*e*-2), 1.38 (1H, dm, *J* = 13.6 Hz, H*e*-3), 1.30 (1H, qd, *J* = 12.6, 3.9 Hz, H*a*-6), 1.21 (1H, td, *J* = 13.6, 4.6 Hz, H*a*-3), 1.18 (1H, td, *J* = 13.6, 3.4 Hz, H*a*-1), 1.12 (1H, dd, *J* = 12.6, 2.5 Hz, H-5), 0.70 (3H, s, H_3_-13), 0.79 (3H, s, H_3_-14), 0.85 (3H, s, H_3_-15). ^13^C NMR (100 MHz, CD_3_OD)δ: 195.4 (C-1′), 161.2 (C-3′), 150.5 (C-8), 120.2 (C-2′), 107.4 (C-12), 66.2 (C-4′), 62.3 (C-5′), 62.2 (C-7′), 61.2 (C-6′), 56.9 (C-5), 52.9 (C-9), 43.3 (C-3), 40.7 (C-10), 40.0 (C-1), 39.3 (C-7), 34.5 (C-4), 34.1 (C-13), 25.6 (C-6), 22.2 (C-14), 22.0 (C-11), 20.4 (C-2), 15.0 (C-15). The above ^1^H and ^13^C NMR data were assigned on the basis of DEPT, ^1^H–^1^H COSY, HMQC, HMBC and NOESY experiments.

### 3.7. HPLC Analysis for *1–3* and the G59 and BD-1-6 Extracts

The EtOAc extracts of G59 and BD-1-6 cultures and the compounds **1**–**3** were dissolved in MeOH to prepare a 10 mg/mL sample solution for HPLC analysis. HPLC analysis of the samples was carried out on a Venusil MP C18 column (5 μm, 100 Å, 4.6 mm × 250 mm; Agela Technologies) using the same Waters HPLC equipment mentioned. Each 5 μL of sample solutions was injected into the column after filtration by a 0.22 μm pore membrane filter. Then, the elution was performed using MeOH–H_2_O in linear gradient (20% MeOH at initial time 0 min→100% MeOH at 60 min→100% MeOH at 90 min; flow rate, 1 mL/min). The acquired photodiode array (PDA) data were processed by Empower PDA software to obtain targeted HPLC data.

### 3.8. LC-ESIMS Analysis for *1–3* and the G59 and BD-1-6 Extracts

The LC-ESIMS analysis was performed on a LC-MS equipment equipped with Agilent 1100 HPLC system, AB Sciex API 3000 LC-MS/MS system, and AB Sciex Analyst 1.4 software. The above mentioned EtOAc extracts of G59 and BD-1-6, at 10 mg/mL in MeOH, were employed also for the LC-ESIMS analysis and crude **1**–**3** samples were used as control. HPLC was carried out on a Venusil MP C18 column (5 μm, 100 Å, 4.6 mm × 250 mm; Agela Technologies) using MeOH–H_2_O in linear gradient (20% MeOH at initial time 0 min→100% MeOH at 60 min→100% MeOH at 90 min; flow rate, 1 mL/min) as mobile phase. The mass detector was set to scan a range from *m/z* 150 to 1500 in positive (for **1**–**3**) or negative (for **1**) mode. The acquired data were processed by Analyst 1.4 software to obtain targeted LC-ESIMS data.

## 4. Conclusions

Two new drimenyl cyclohexenone derivatives, purpurogemutantin (**1**) and purpurogemutantidin (**2**), and the known macrophorin A (**3**) were isolated from a mutant BD-1-6 obtained by random DES mutagenesis of a marine-derived fungal strain *Penicillium purpurogenum* G59. The structures and absolute configurations of **1** and **2** were determined by extensive spectroscopic methods, especially 2D NMR and ECD analysis. Possible biosynthetic pathways for **1**–**3** were also proposed and discussed. Compounds **1** and **2** significantly inhibited human cancer K562, HL-60, HeLa, BGC-823 and MCF-7 cells, and **3** also inhibited the K562 and HL-60 cells. Both bioassay and chemical analysis (HPLC and LC-ESIMS) demonstrated that the parent strain G59 did not produce **1**–**3**, and that the DES-induced mutation(s) in BD-1-6 activated some silent biosynthetic pathways in the parent strain G59, including one set for the **1**–**3** production.

## Supplementary Files

Supplementary File 1: ZIP-Document (ZIP, 8582 KB)
