# Supplementary material for: Purpurogemutantin and Purpurogemutantidin, New Drimenyl Cyclohexenone Derivatives Produced by a Mutant Obtained by Diethyl Sulfate Mutagenesis of a Marine-Derived Penicillium purpurogenum G59
Source: Mar Drugs. 2012 Jun 4;10(6):1266–87. doi: 10.3390/md10061266 (PMC3397438; doi:10.3390/md10061266)

Supplementary Materials

Supplementary Data S1. MS, UV, IR and NMR spectra of 1

Contents

Content	Page
1. Positive and negative ESIMS spectra of **1**	2
2. Positive HRESIMS spectrum of **1**	3
3. UV spectrum of **1**	4
4. IR spectrum of **1**	5
5. ^{1}H NMR spectrum of **1**	6
6. ^{13}C NMR spectrum of **1**	7
7. DEPT spectra of **1**	8
8. ^{1}H-^{1}H COSY spectrum of **1**	10
9. HMQC spectrum of **1**	18
10. HMBC spectrum of **1**	21
11. NOESY spectrum of **1**	24

ESIMS and HRESIMS Spectra of **1**

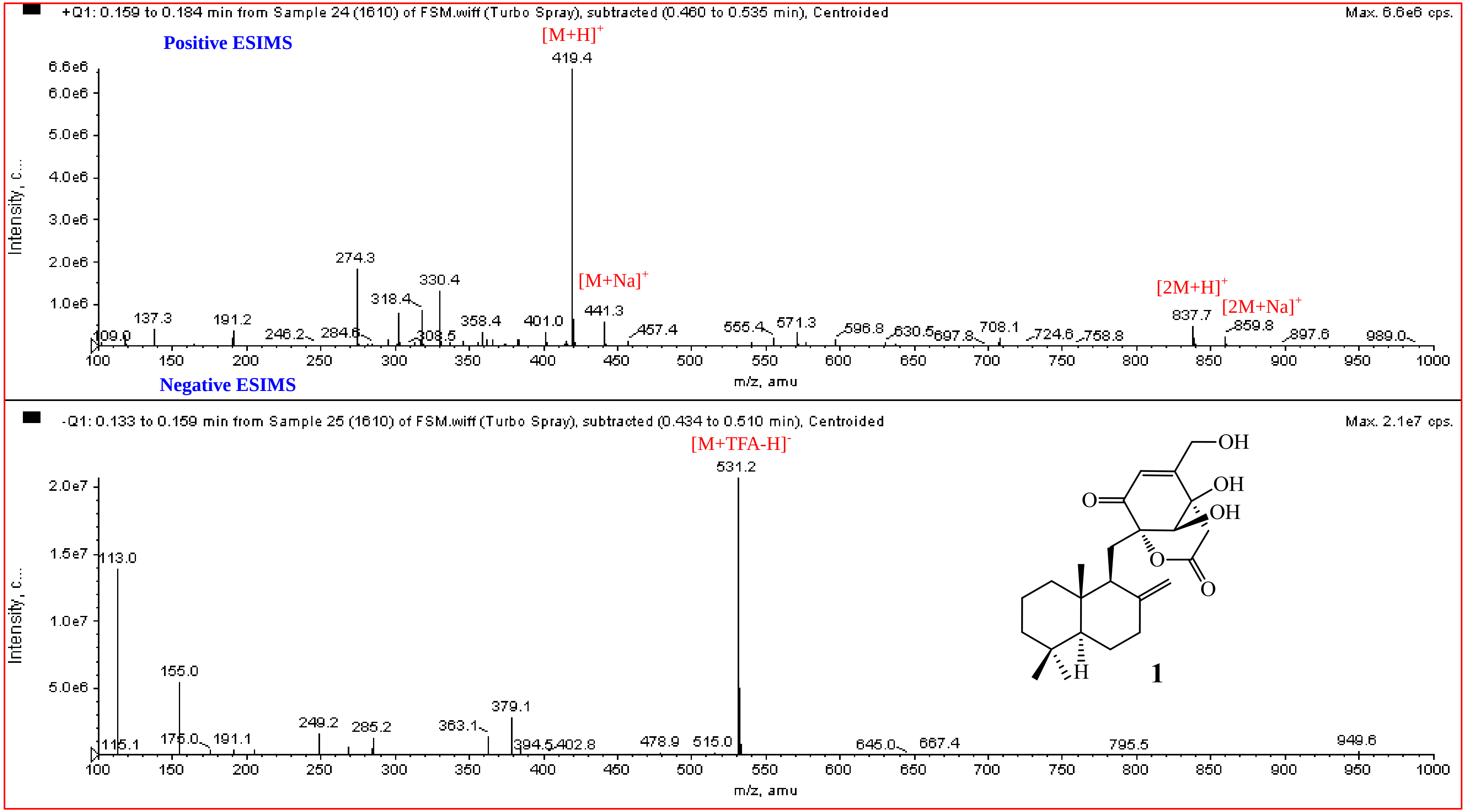

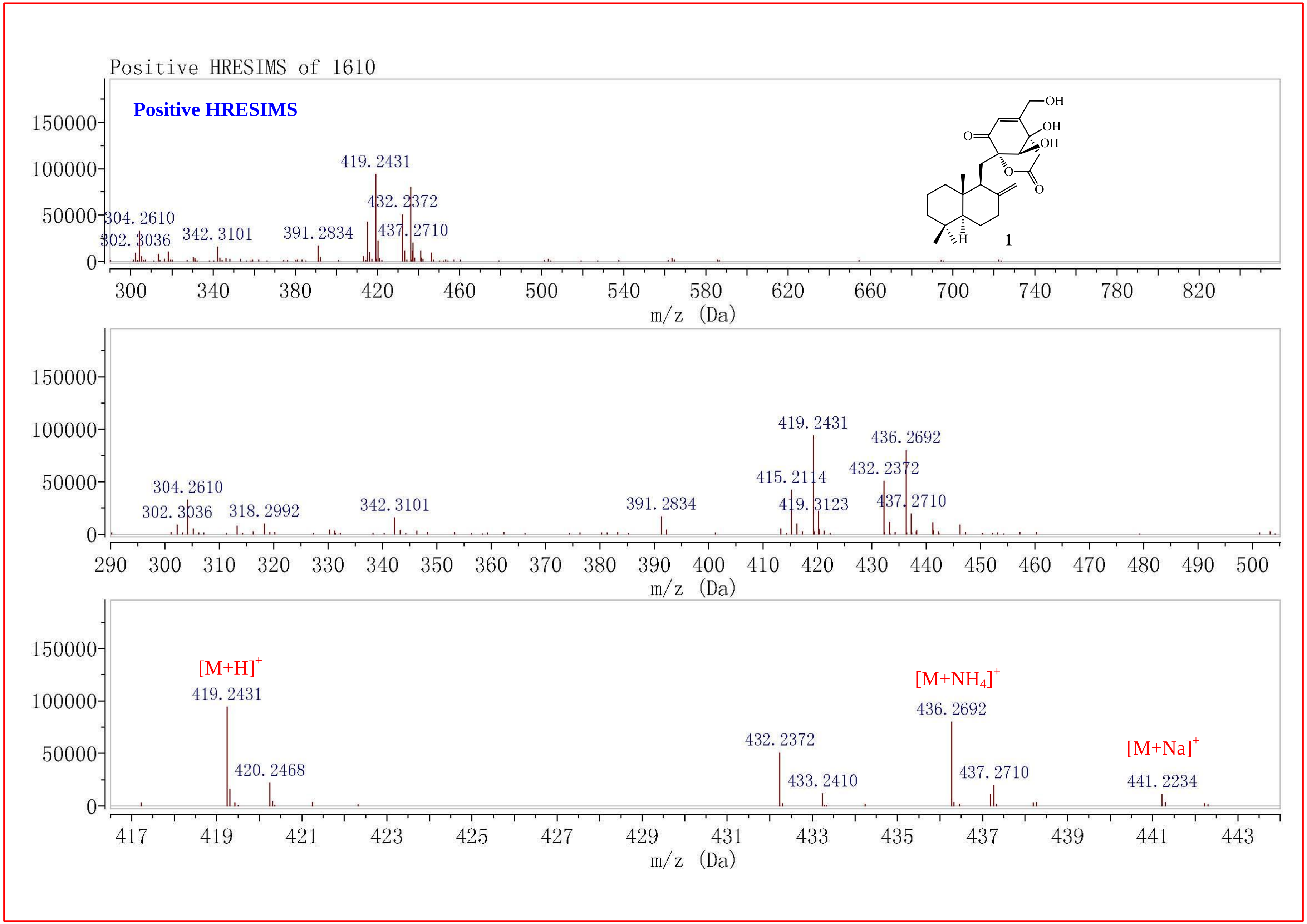
Positive HRESIMS of 1610
Positive HRESIMS
419.2431
432.2372
437.2710
304.2610
302.3036
342.3101
391.2834
OH
OH
OH
O
O
O
H
1
m/z (Da)
419.2431
436.2692
432.2372
415.2114
419.3123
437.2710
304.2610
302.3036
318.2992
342.3101
391.2834
m/z (Da)
[M+H]+
419.2431
420.2468
432.2372
433.2410
[M+NH4]+
436.2692
437.2710
[M+Na]+
441.2234
m/z (Da)

UV Spectrum of **1** in MeOH

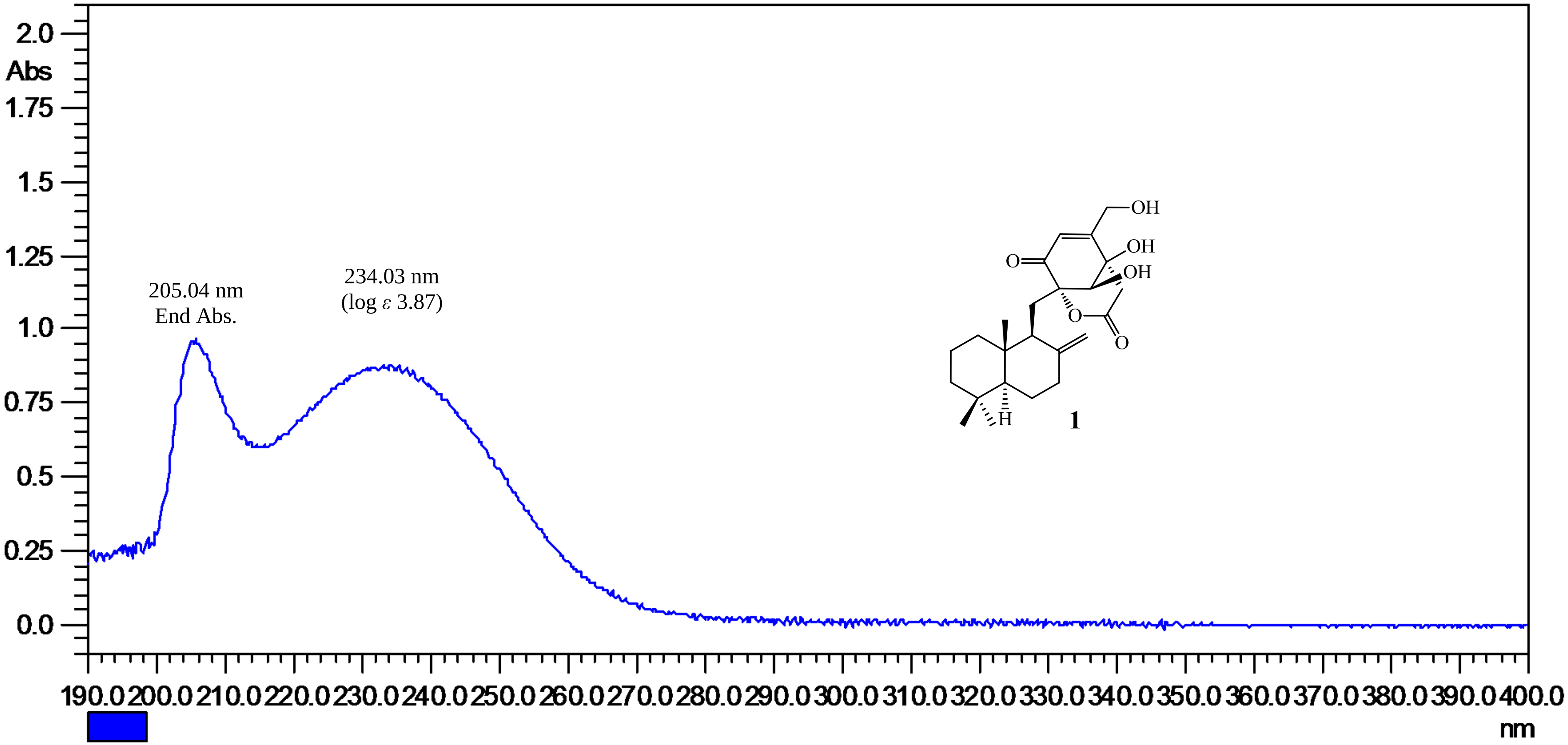

IR Spectrum of **1**

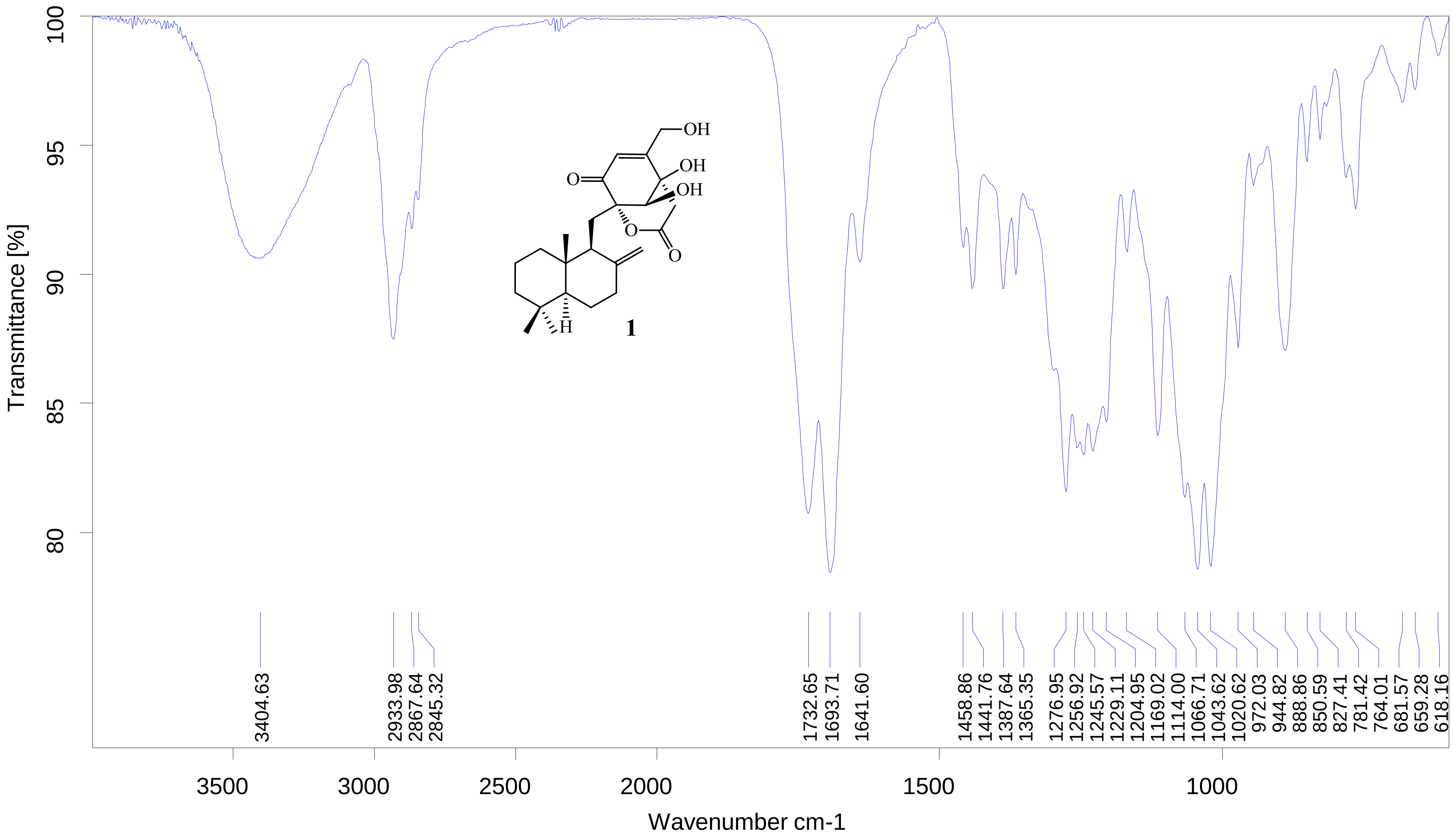

400 MHz ^{1}H NMR Spectrum of **1**

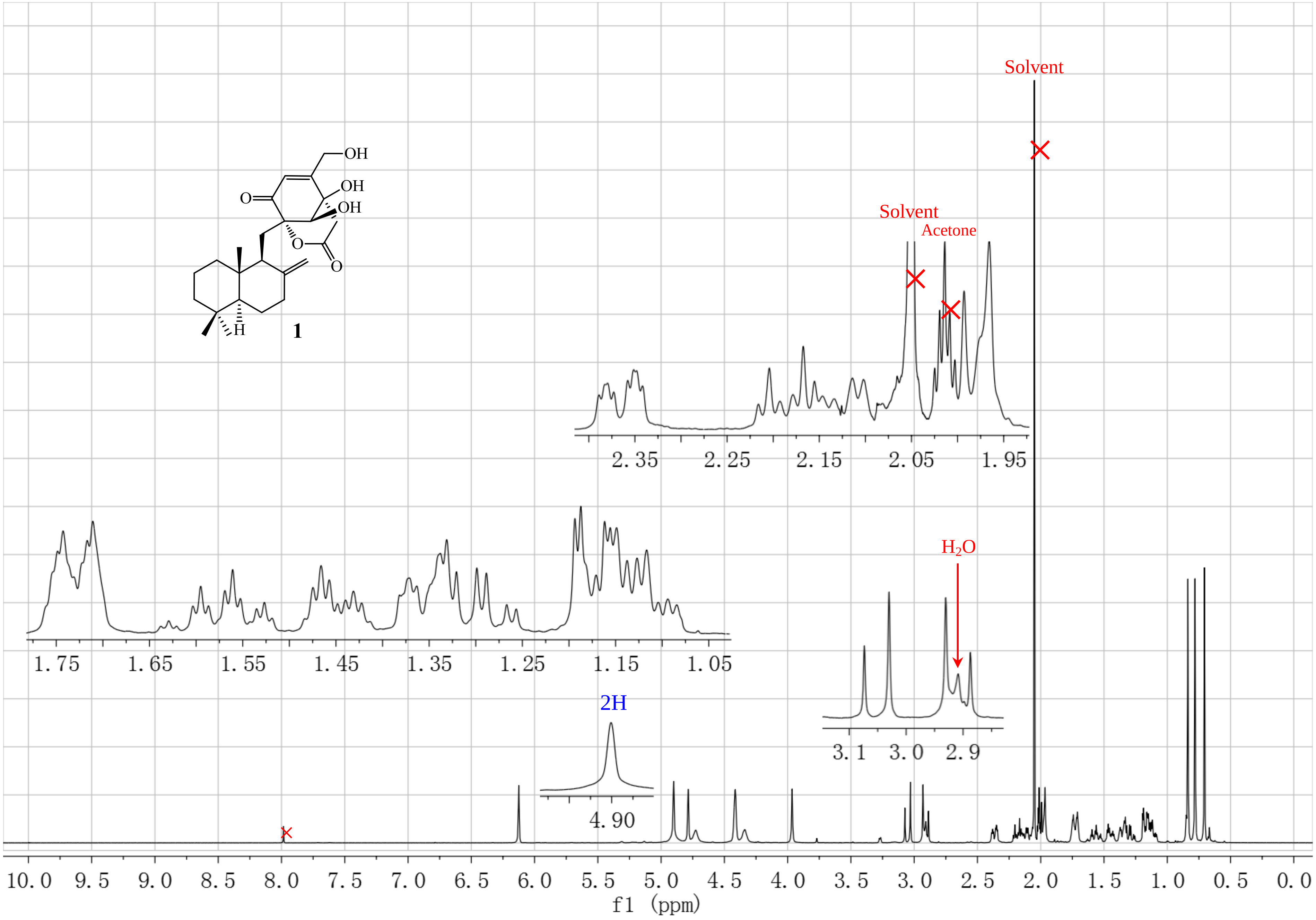

100 MHz ^{13}C NMR Spectrum of **1**

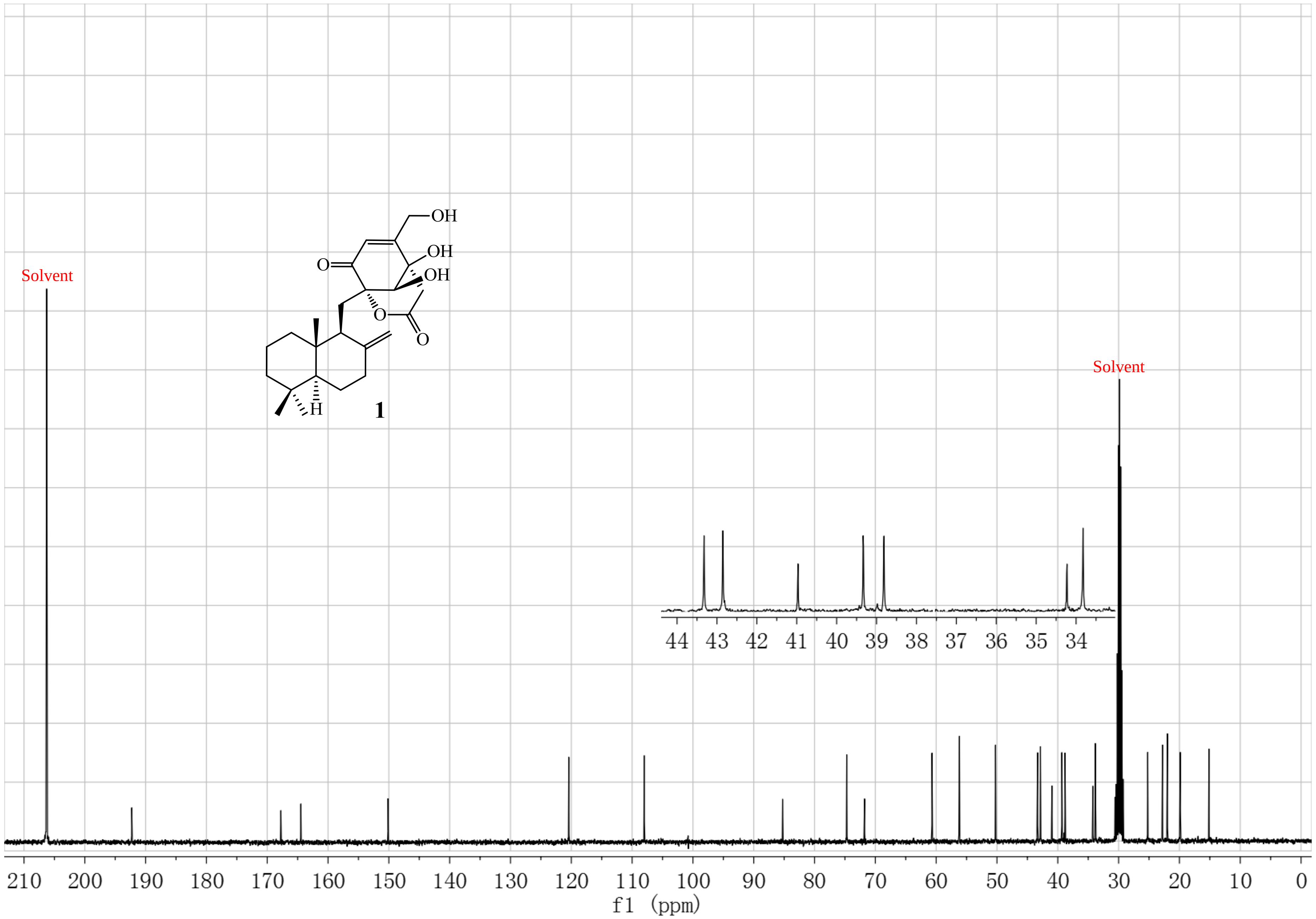

100 MHz ^{13}C NMR and DEPT Spectra of **1** in Acetone-d_6

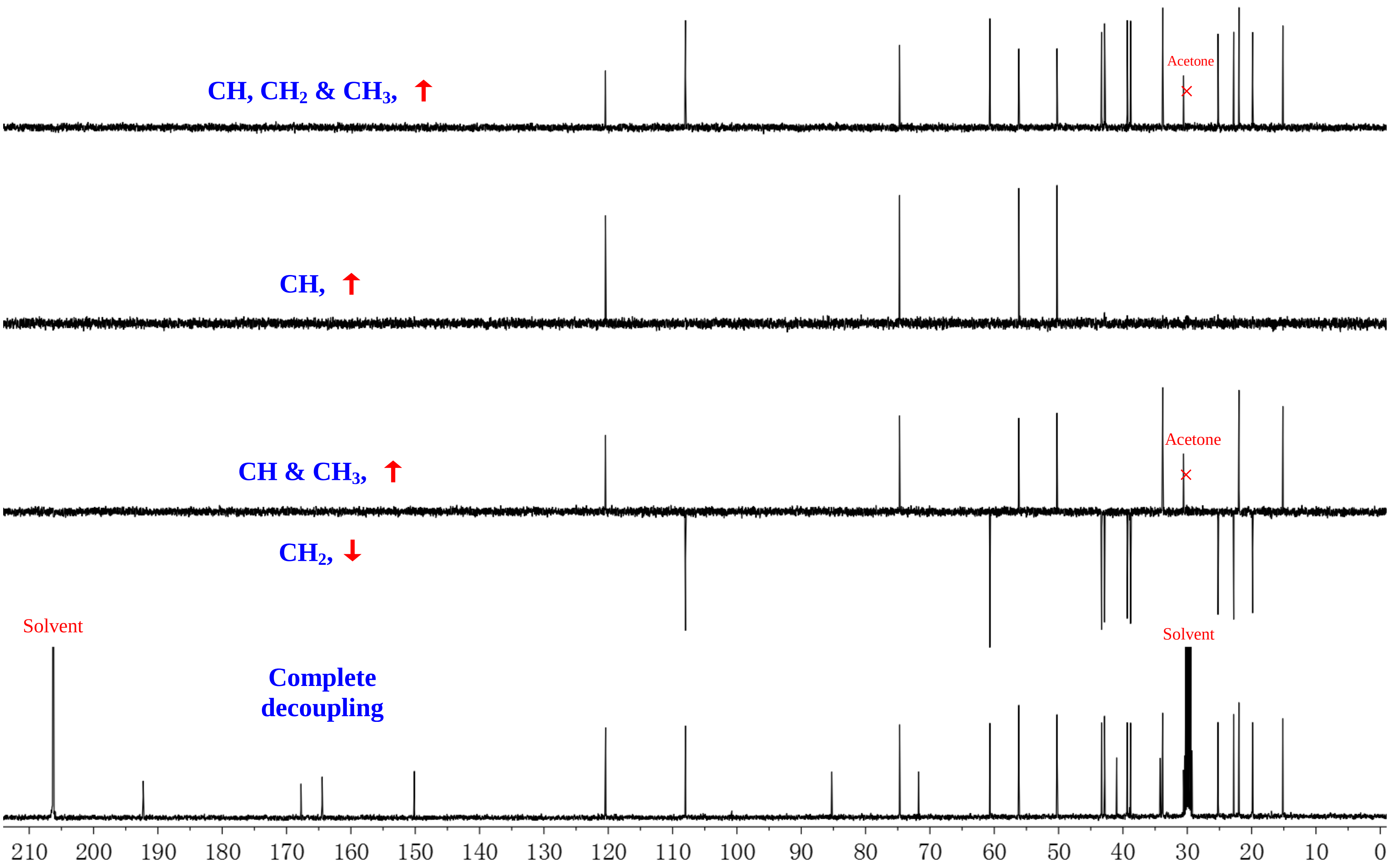

Expanded 100 MHz ^{13}C NMR and DEPT Spectra of **1** in Acetone-d_6

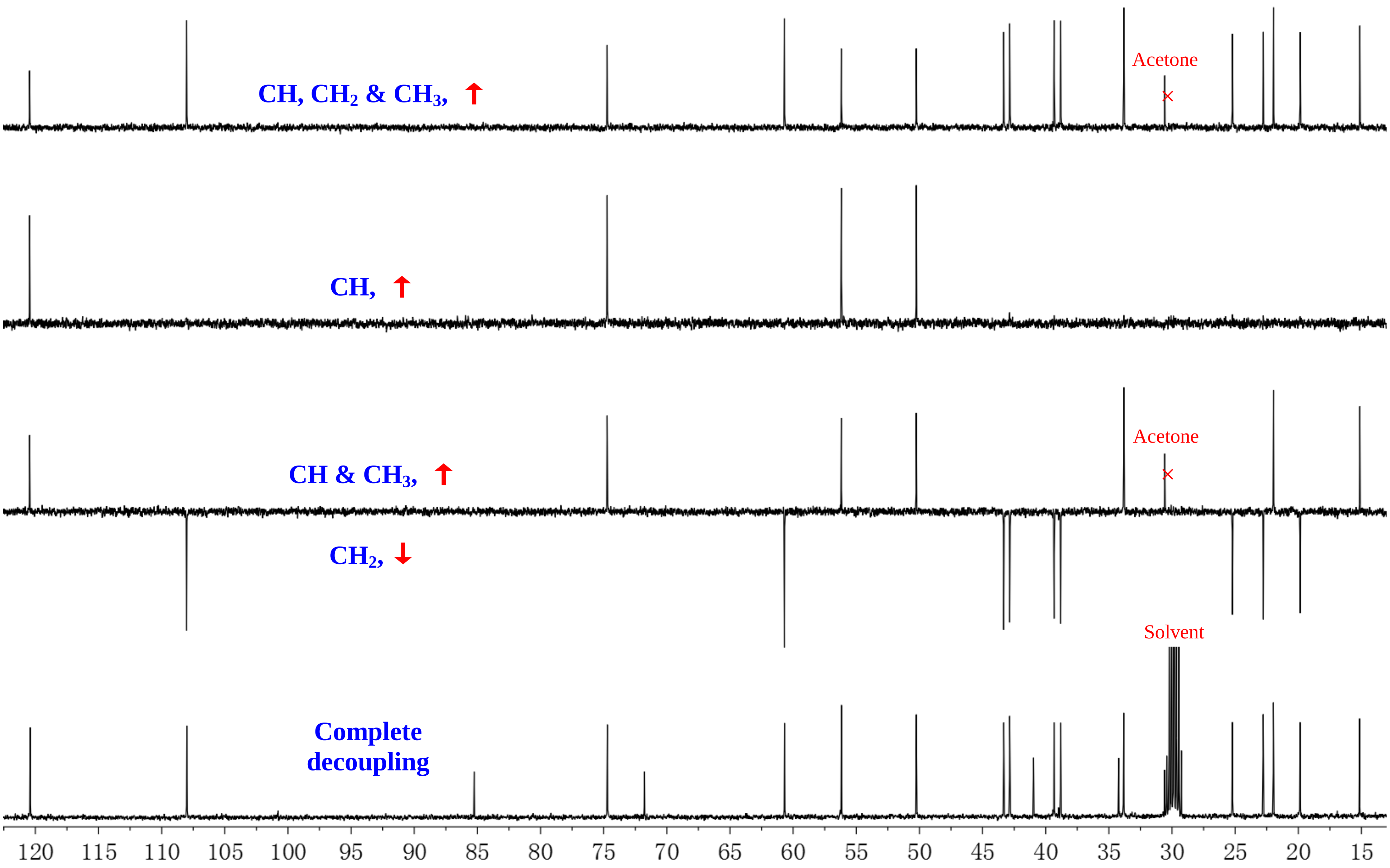

^{1}H-^{1}H COSY Spectrum of **1** in Acetone-d_6

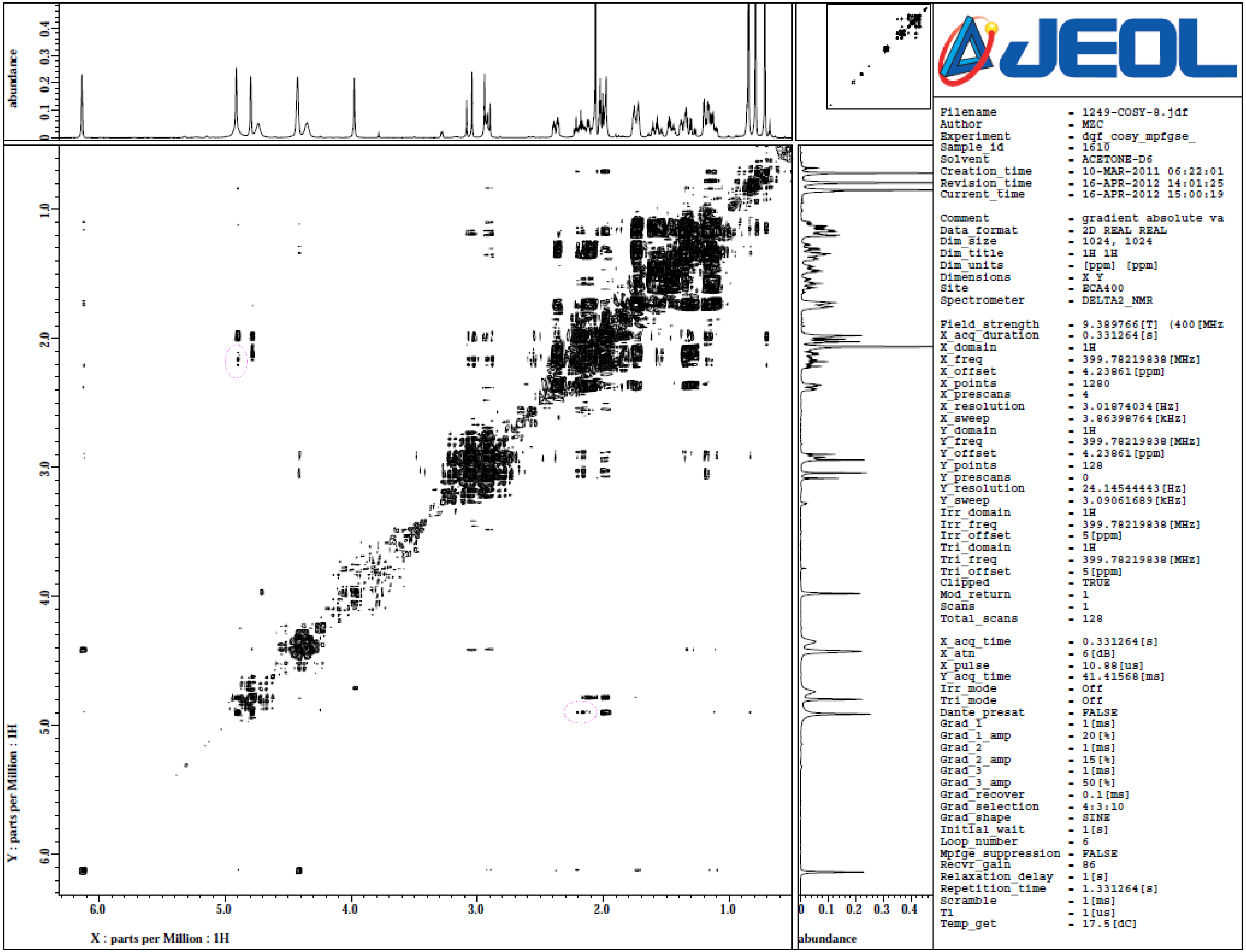

^{1}H-^{1}H COSY Spectrum of **1** in Acetone-d_6

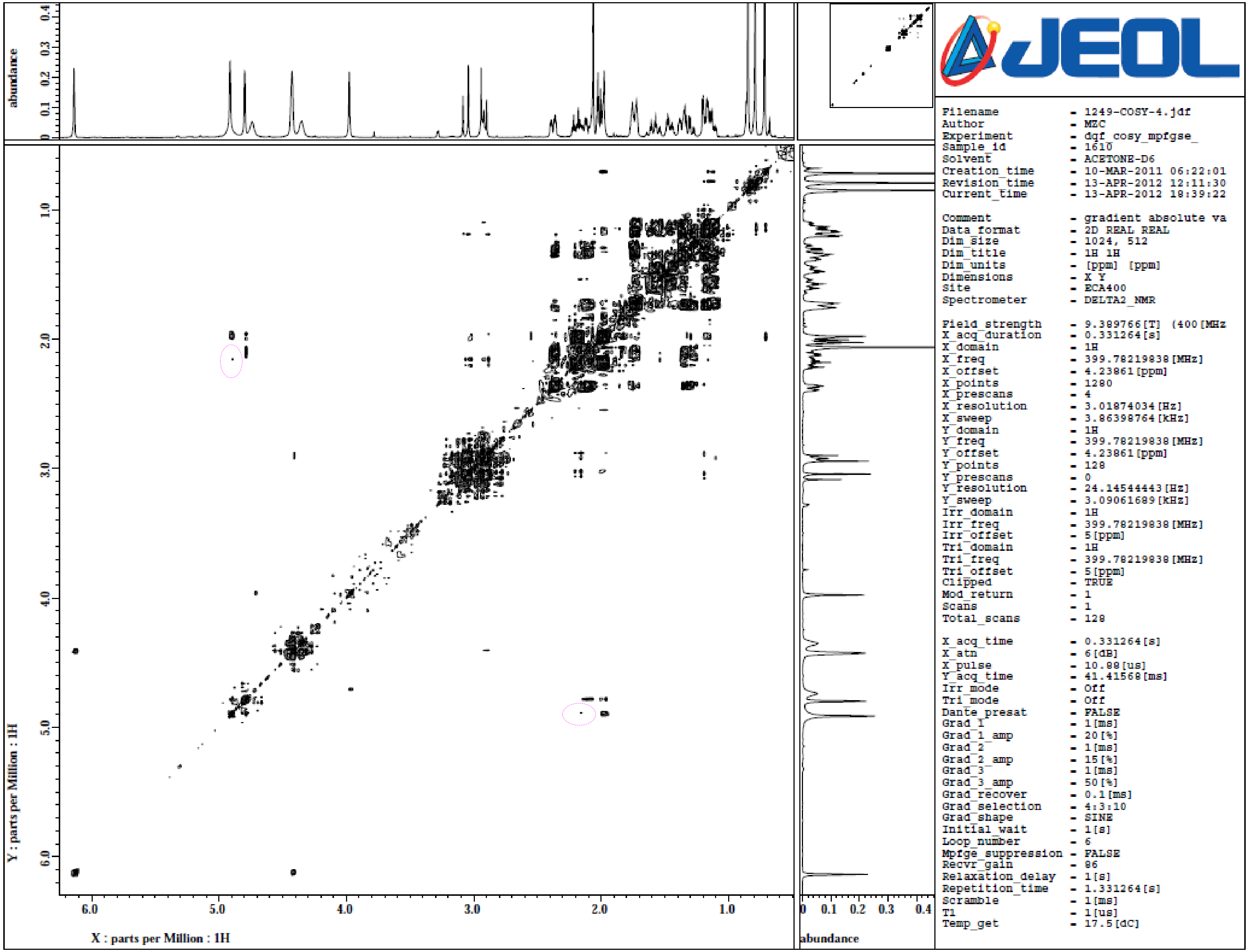

^{1}H-^{1}H COSY Spectrum of **1** in Acetone-d_6

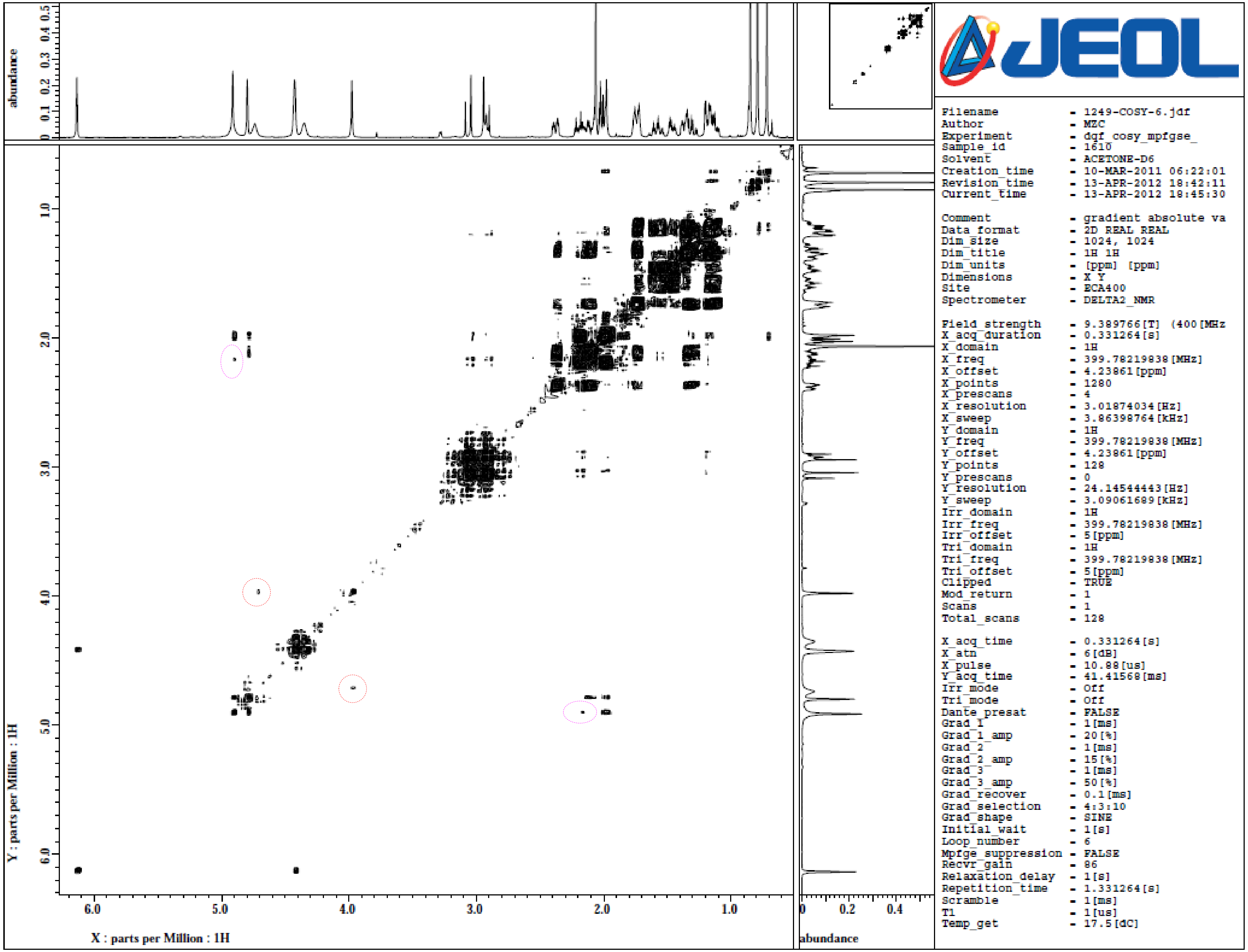

^{1}H-^{1}H COSY Spectrum of **1** in Acetone-d_6

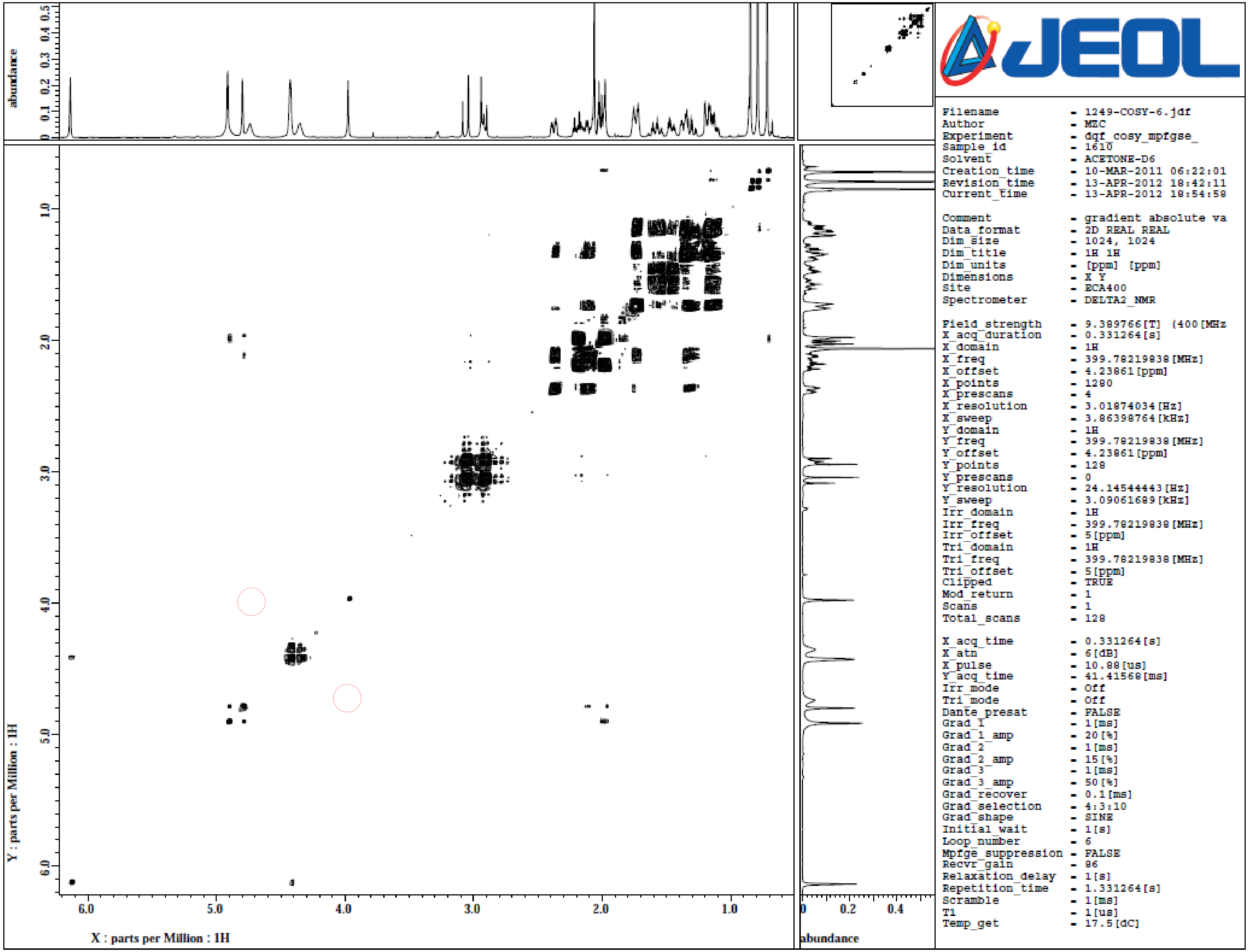

Expanded ^{1}H-^{1}H COSY Spectrum of **1** in Acetone-d_6

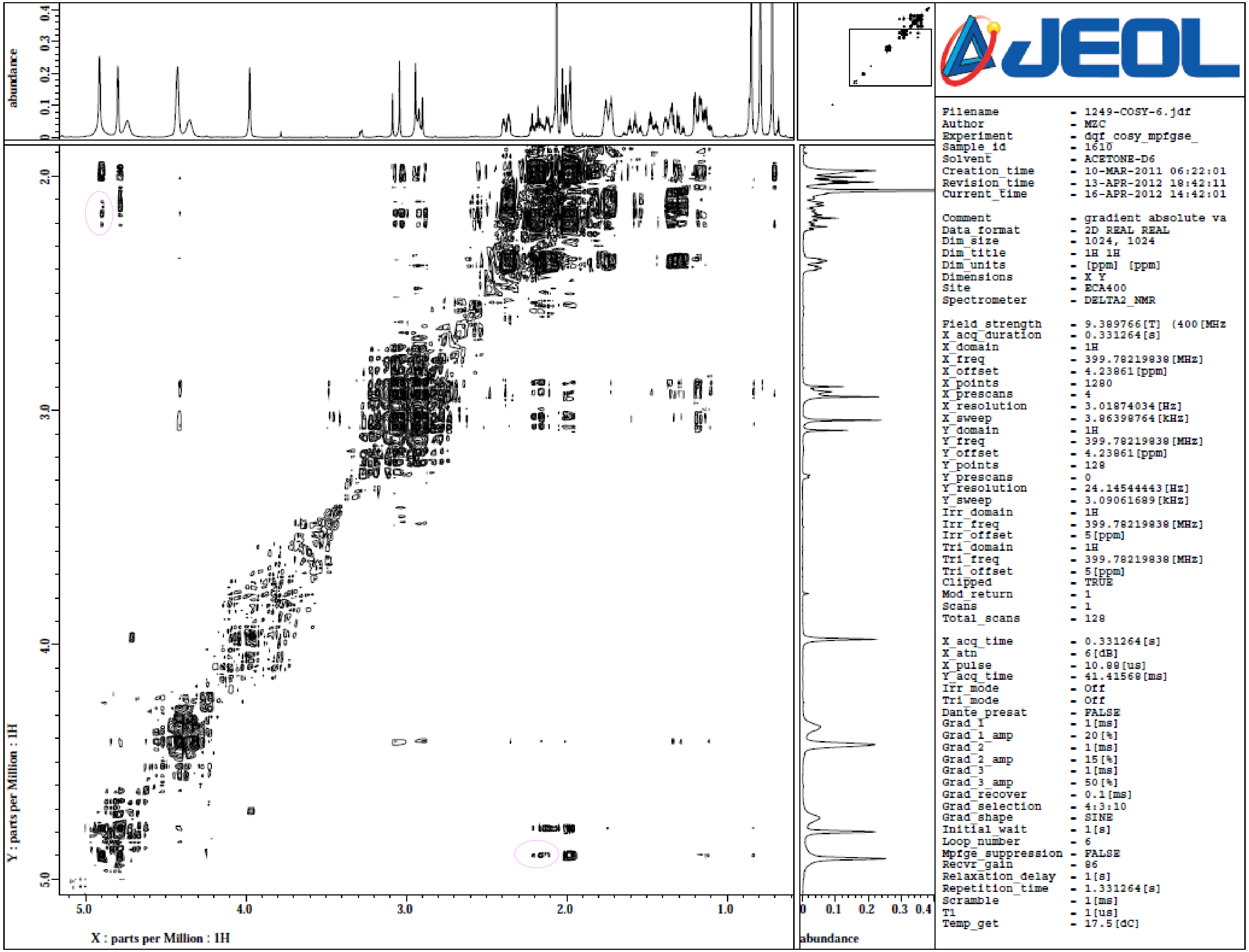

Expanded ^{1}H-^{1}H COSY Spectrum of **1** in Acetone-d_6

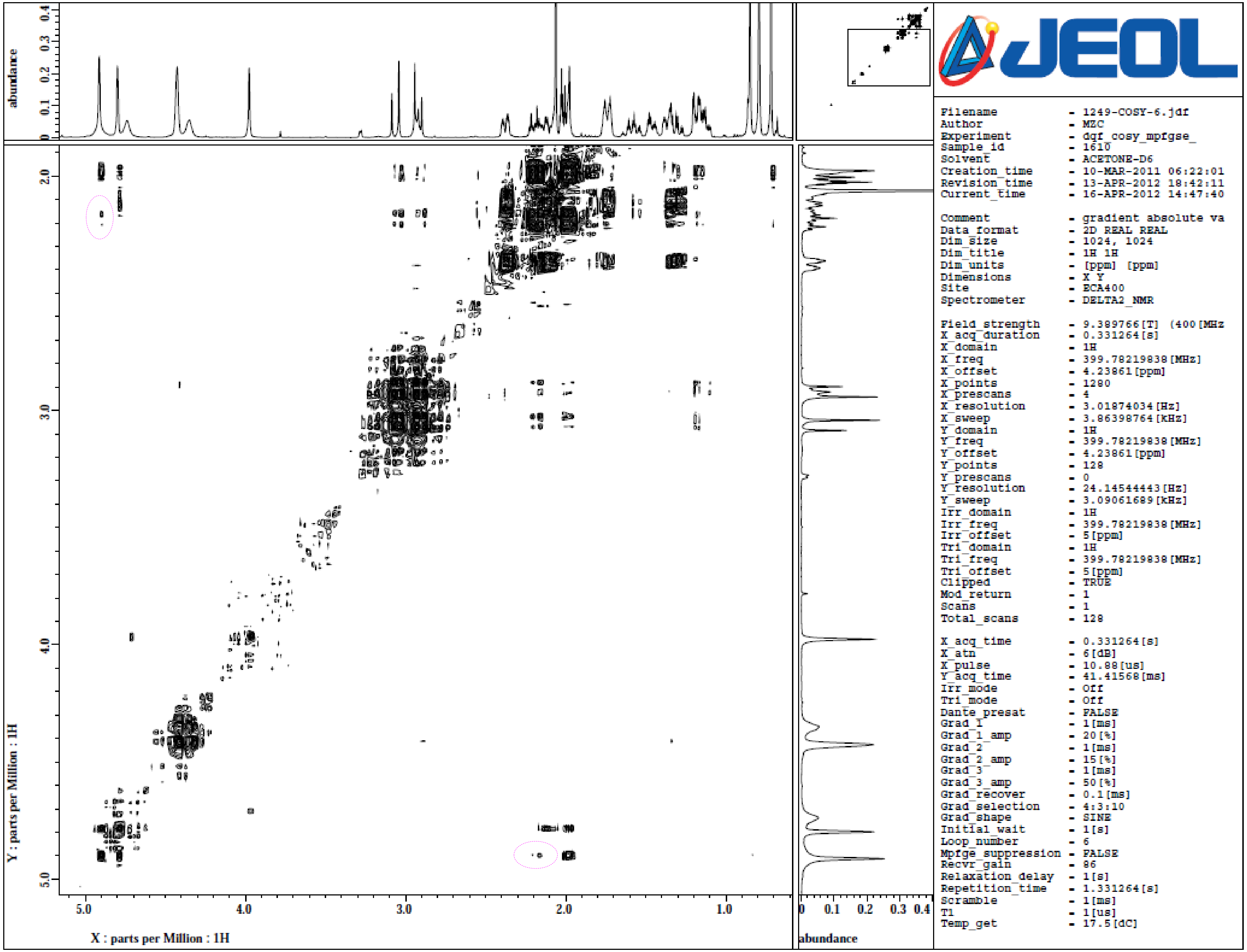

Expanded ^{1}H-^{1}H COSY Spectrum of **1** in Acetone-d_6

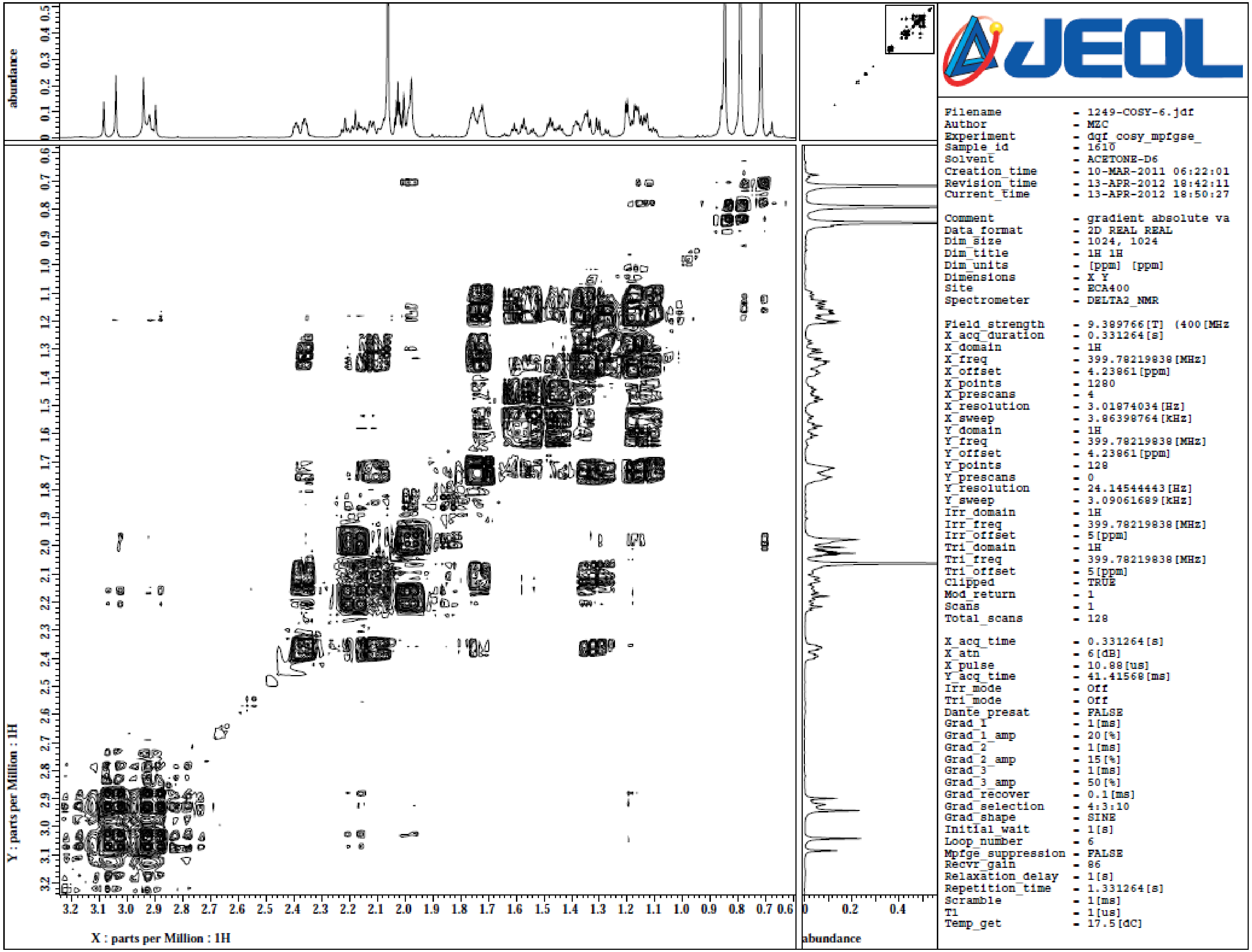

Expanded ^{1}H-^{1}H COSY Spectrum of **1** in Acetone-d_6

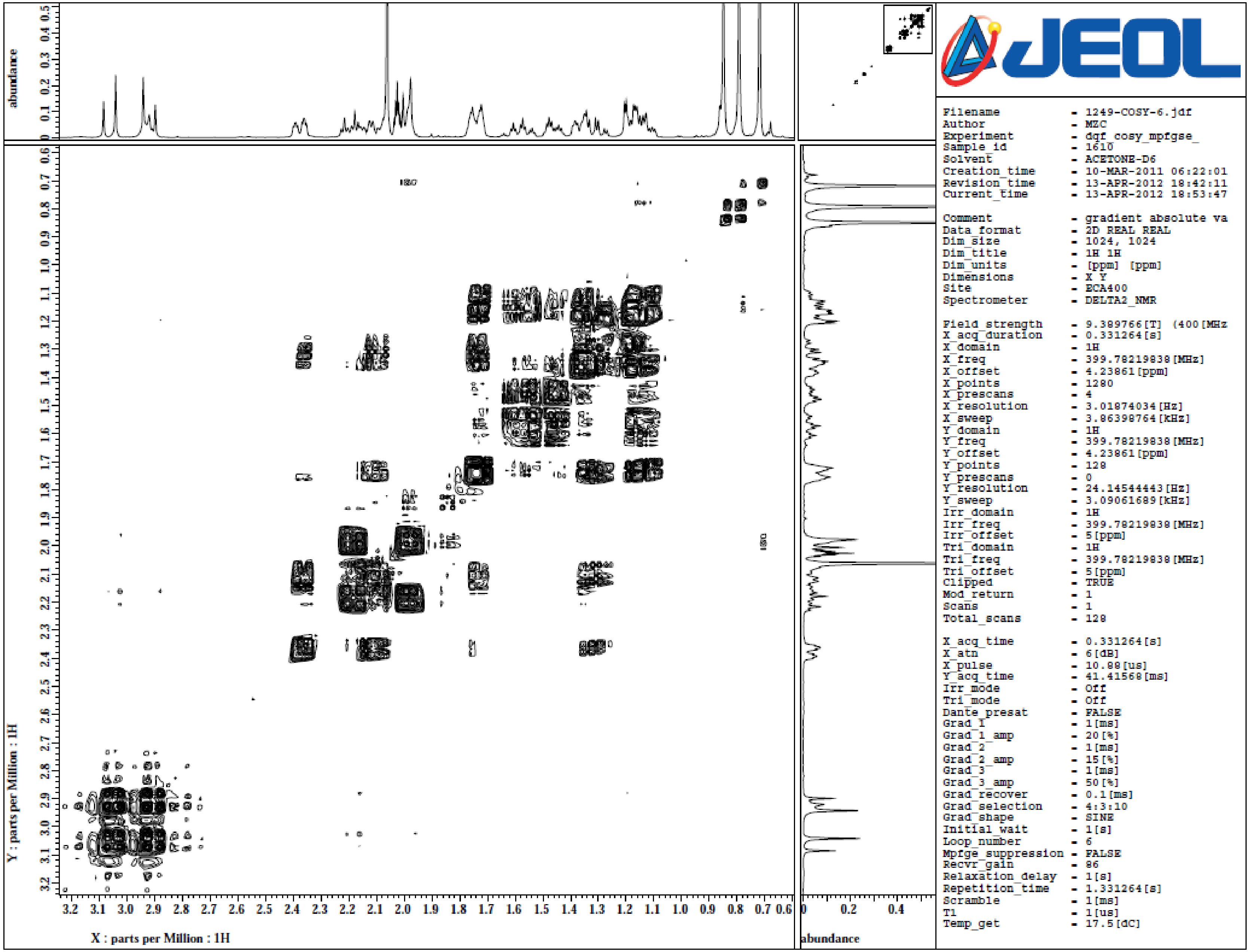

HMQC Spectrum of **1** in Acetone-d_6

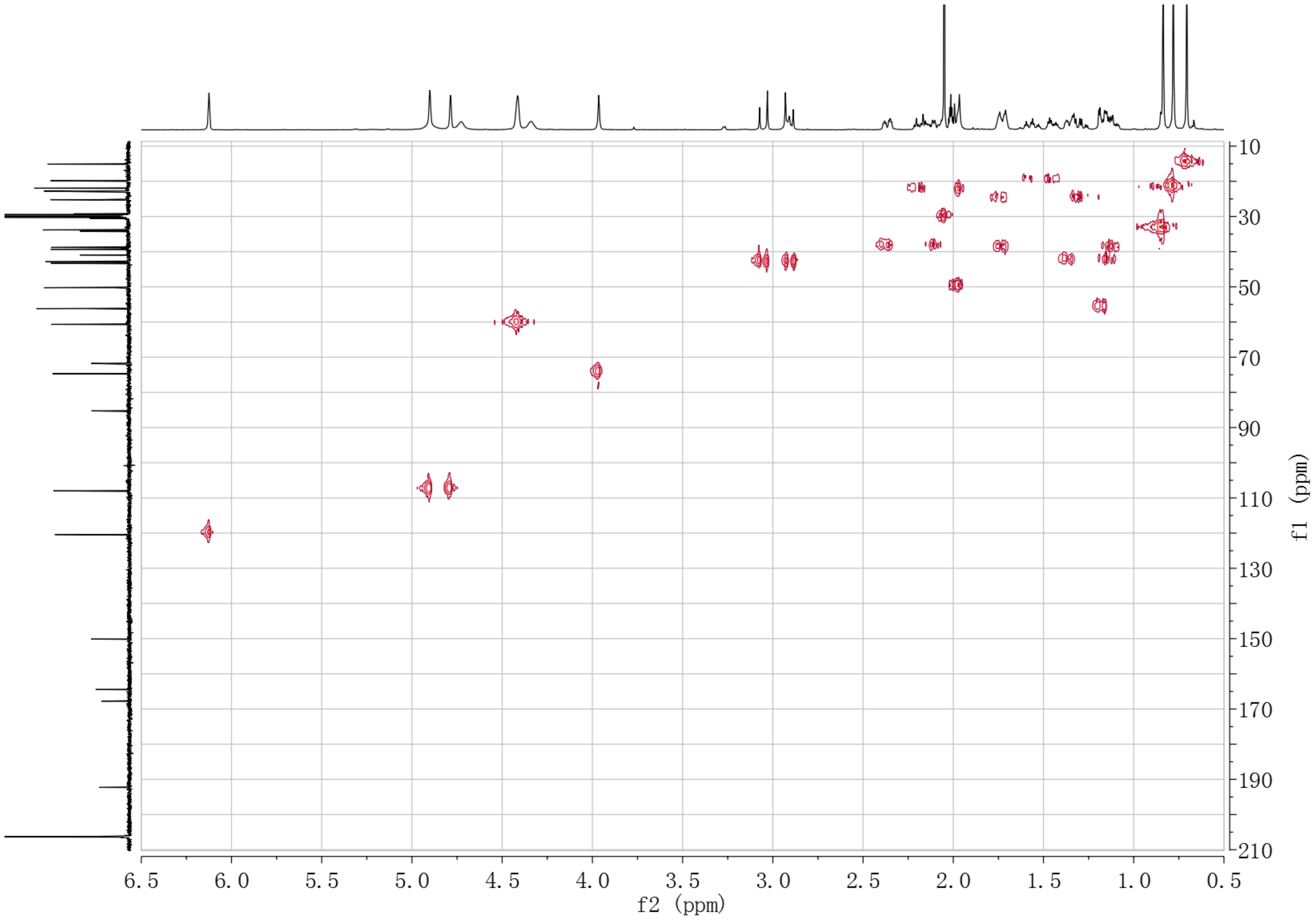

Expanded HMQC Spectrum of **1** in Acetone-d_6

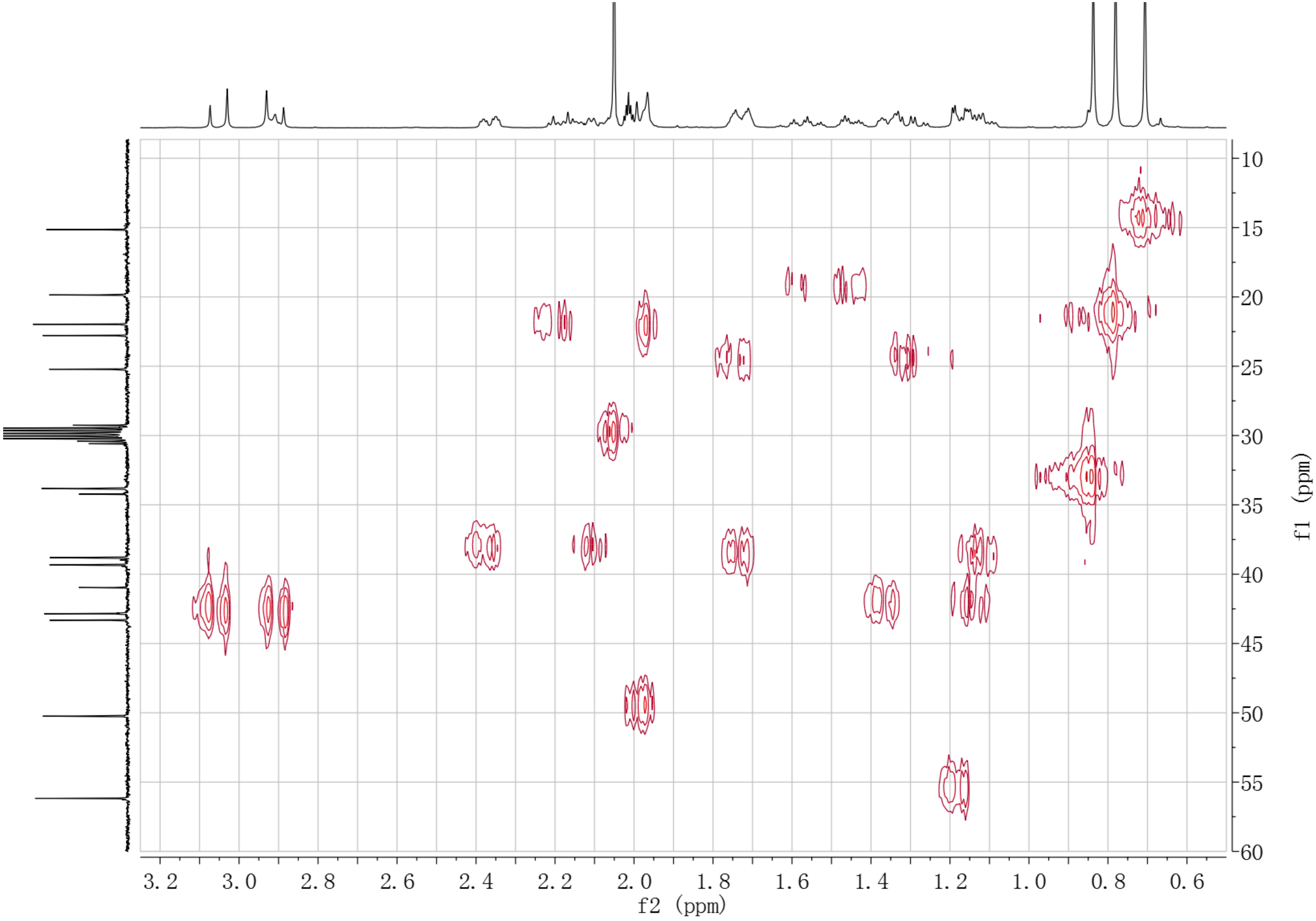

Expanded HMQC Spectrum of **1** in Acetone-d_6

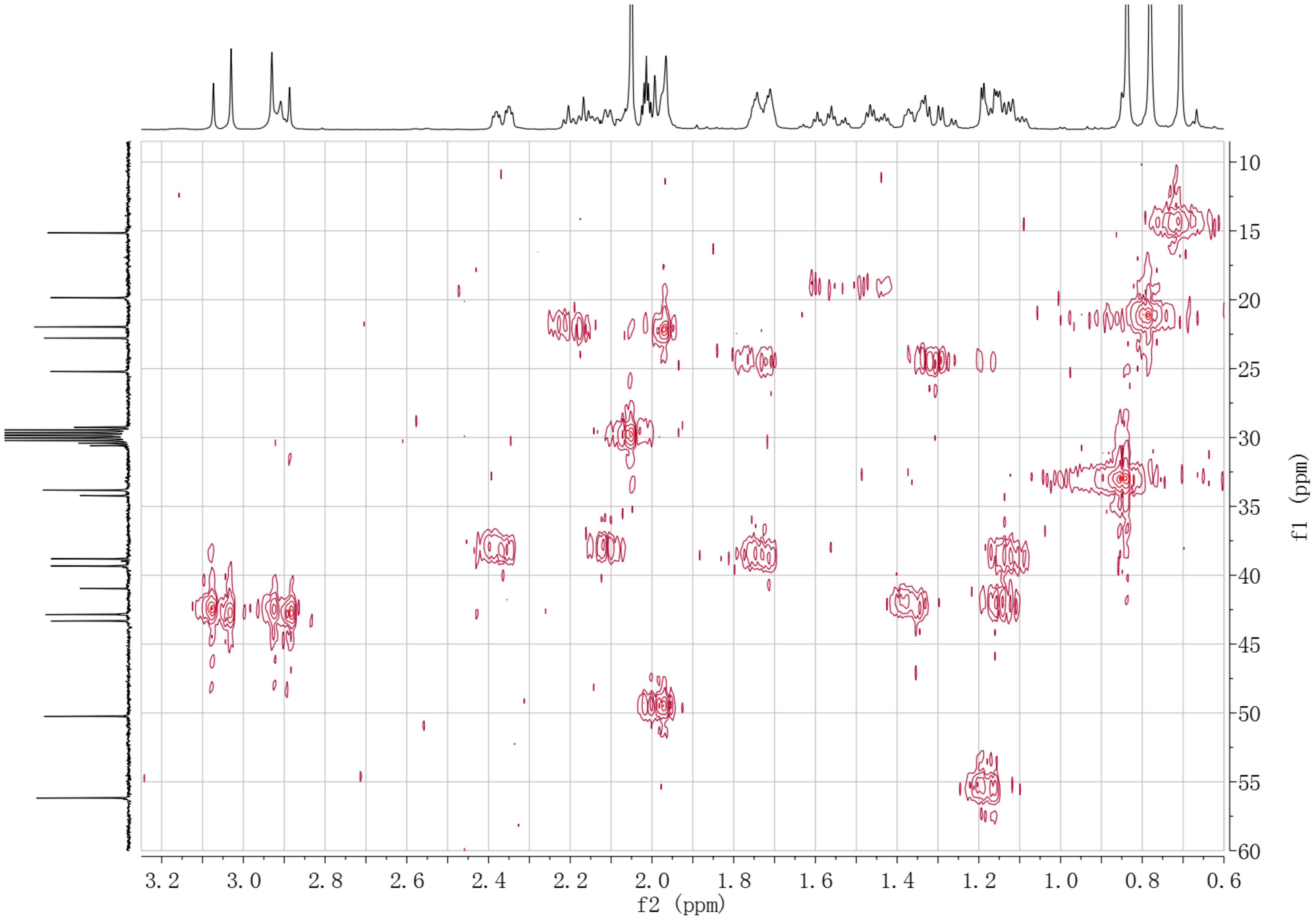

HMBC Spectrum of **1** in Acetone-d_6

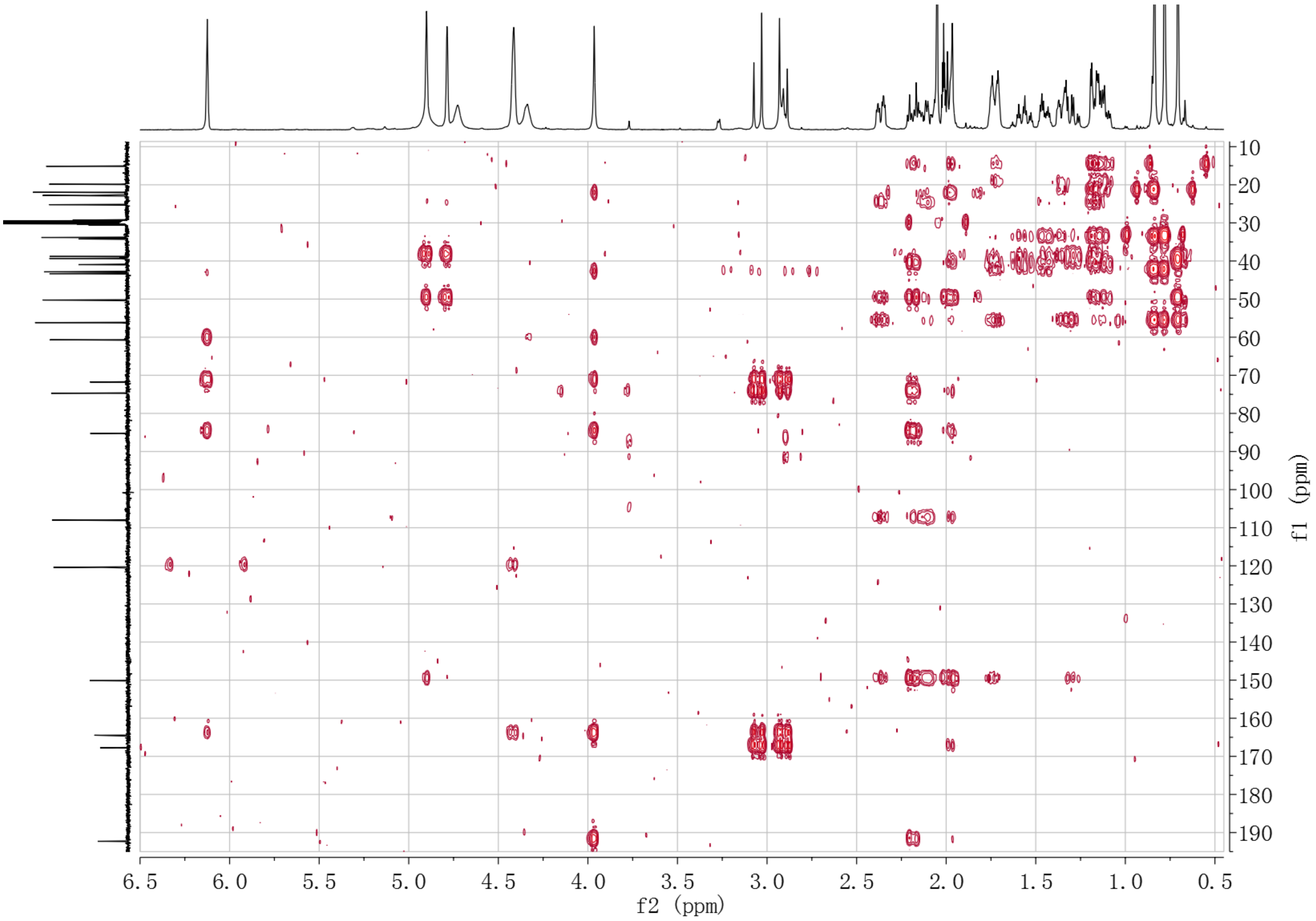

Expanded HMBC Spectrum of **1** in Acetone-d_6

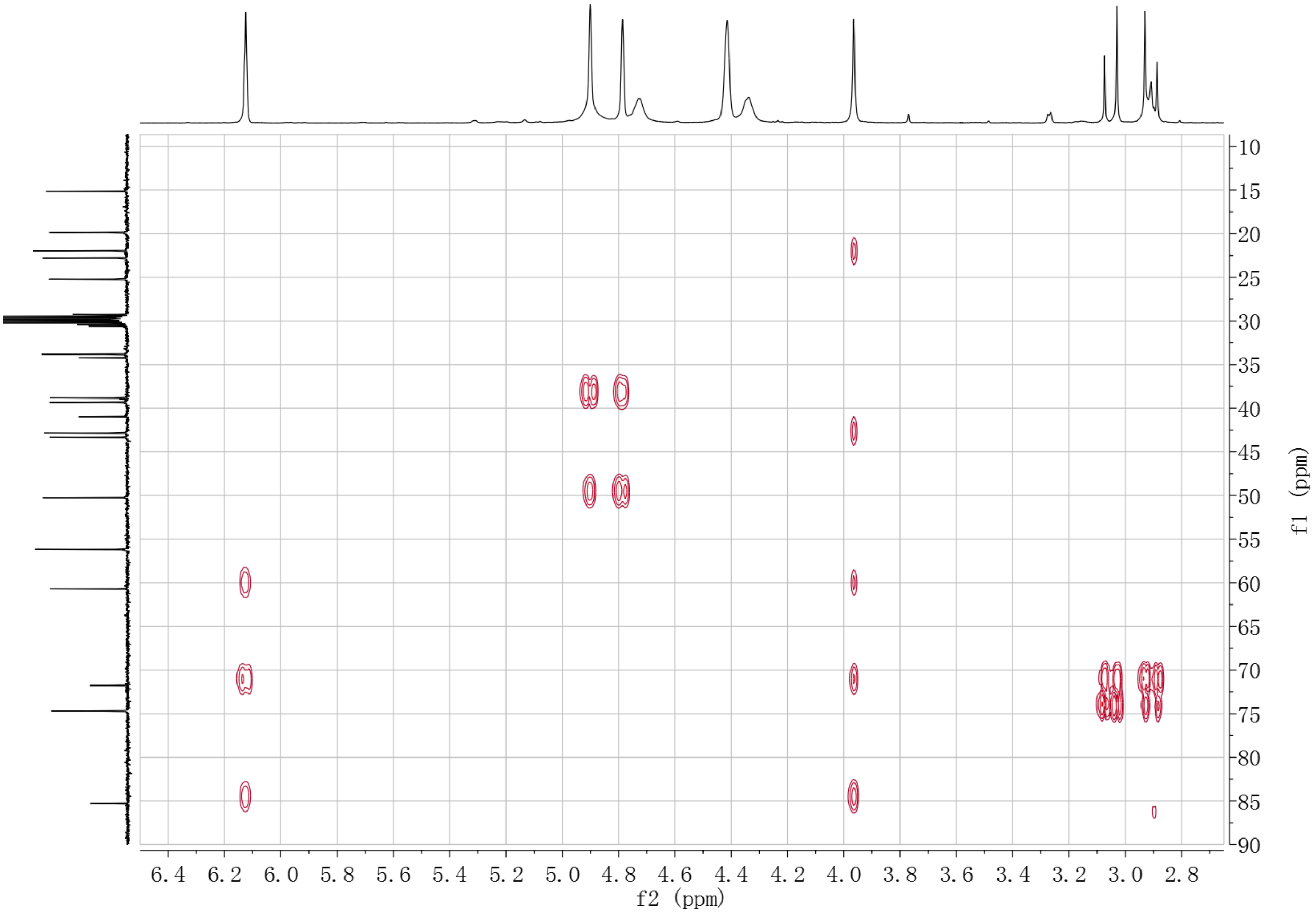

Expanded HMBC Spectrum of **1** in Acetone-d_6

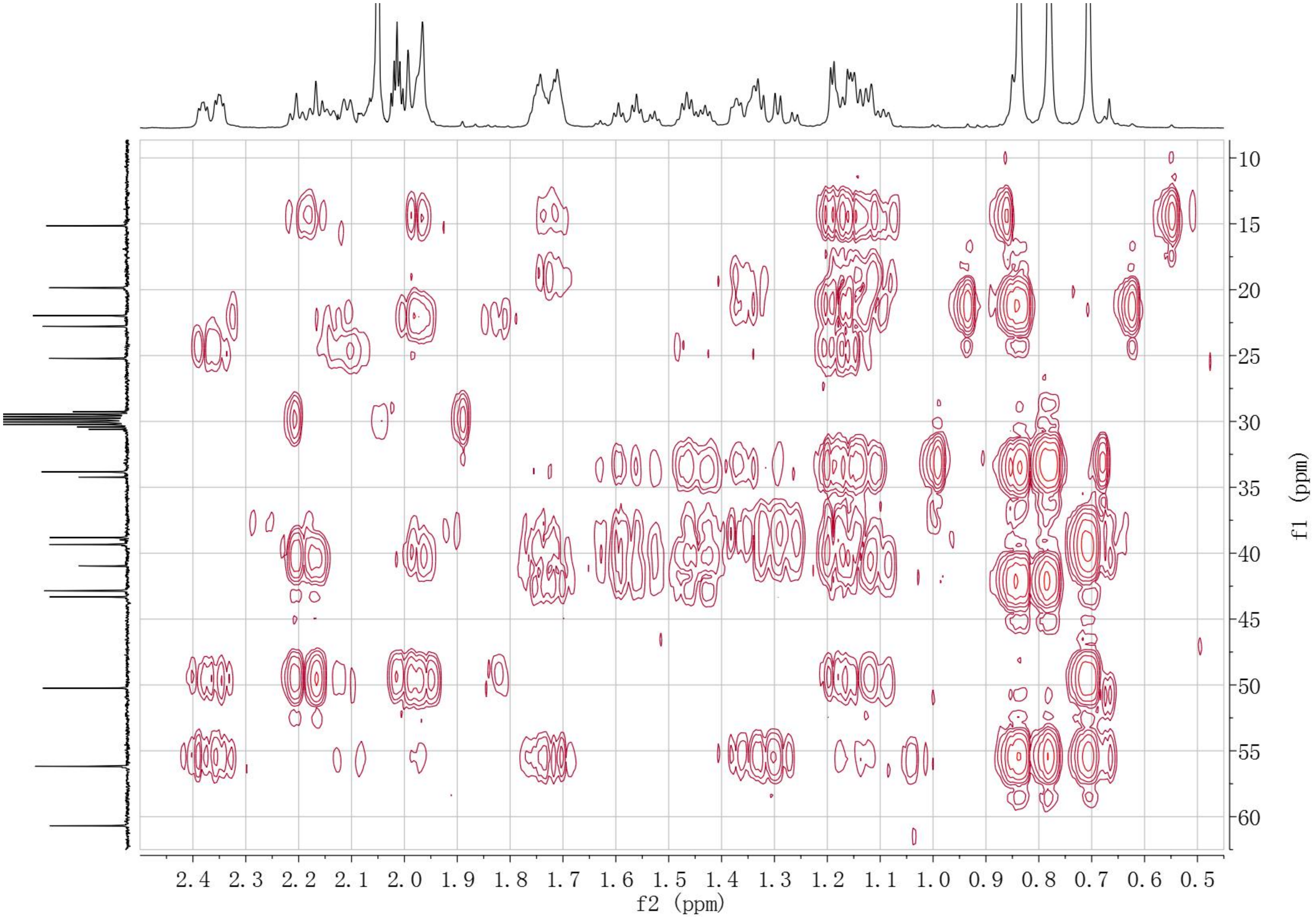

NOESY Spectrum of **1** in Acetone-d_6

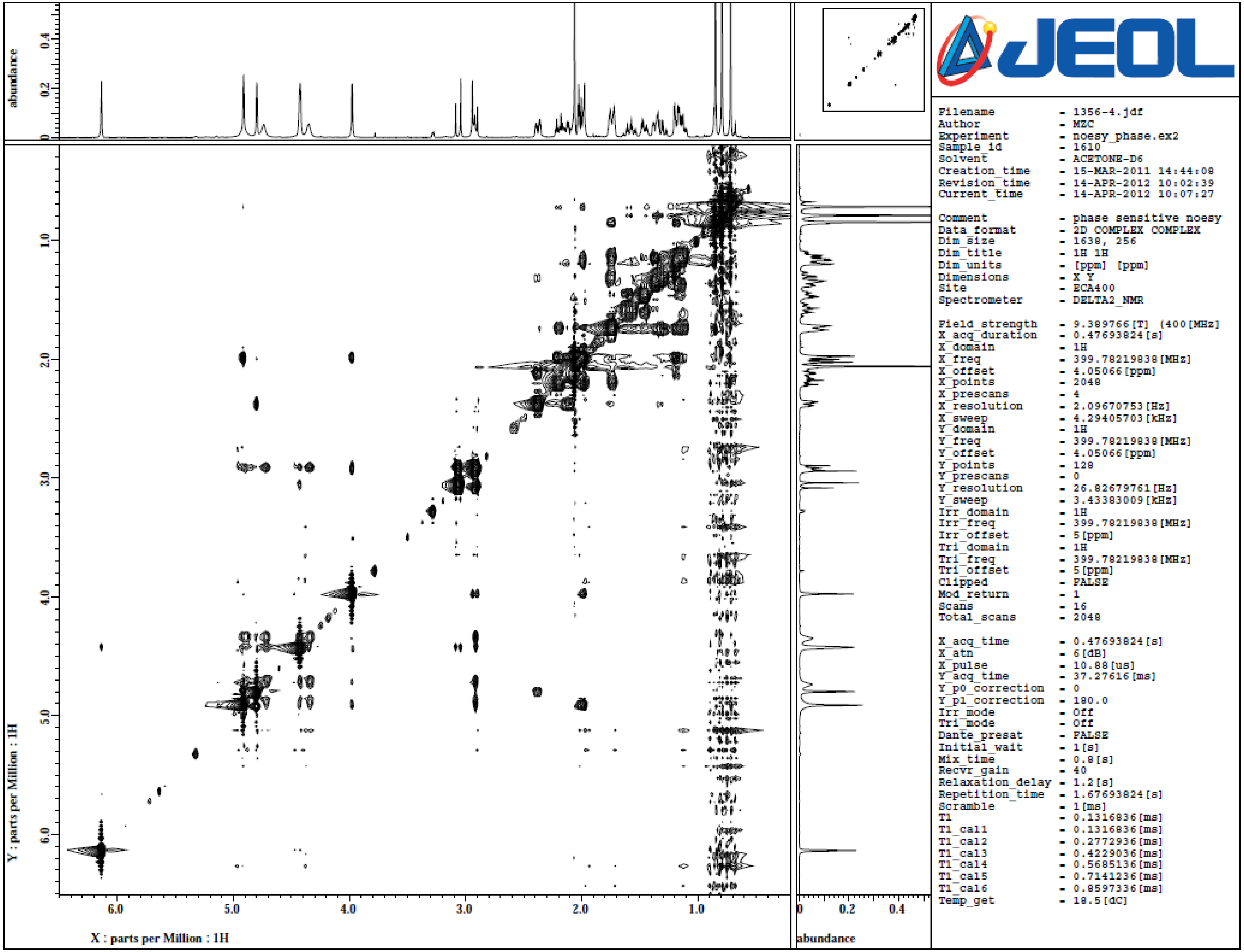

NOESY Spectrum of **1** in Acetone-d_6

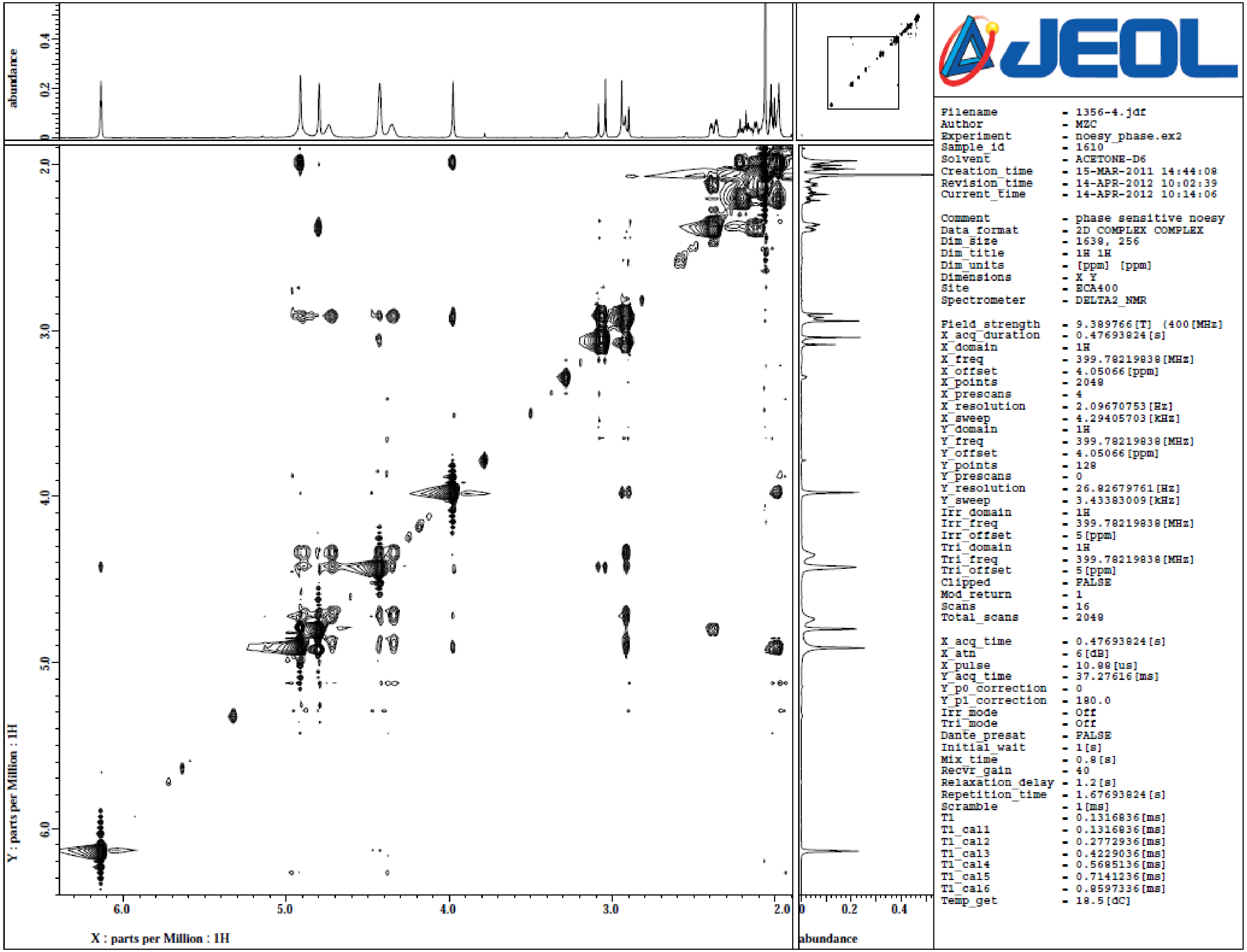

NOESY Spectrum of **1** in Acetone-d_6

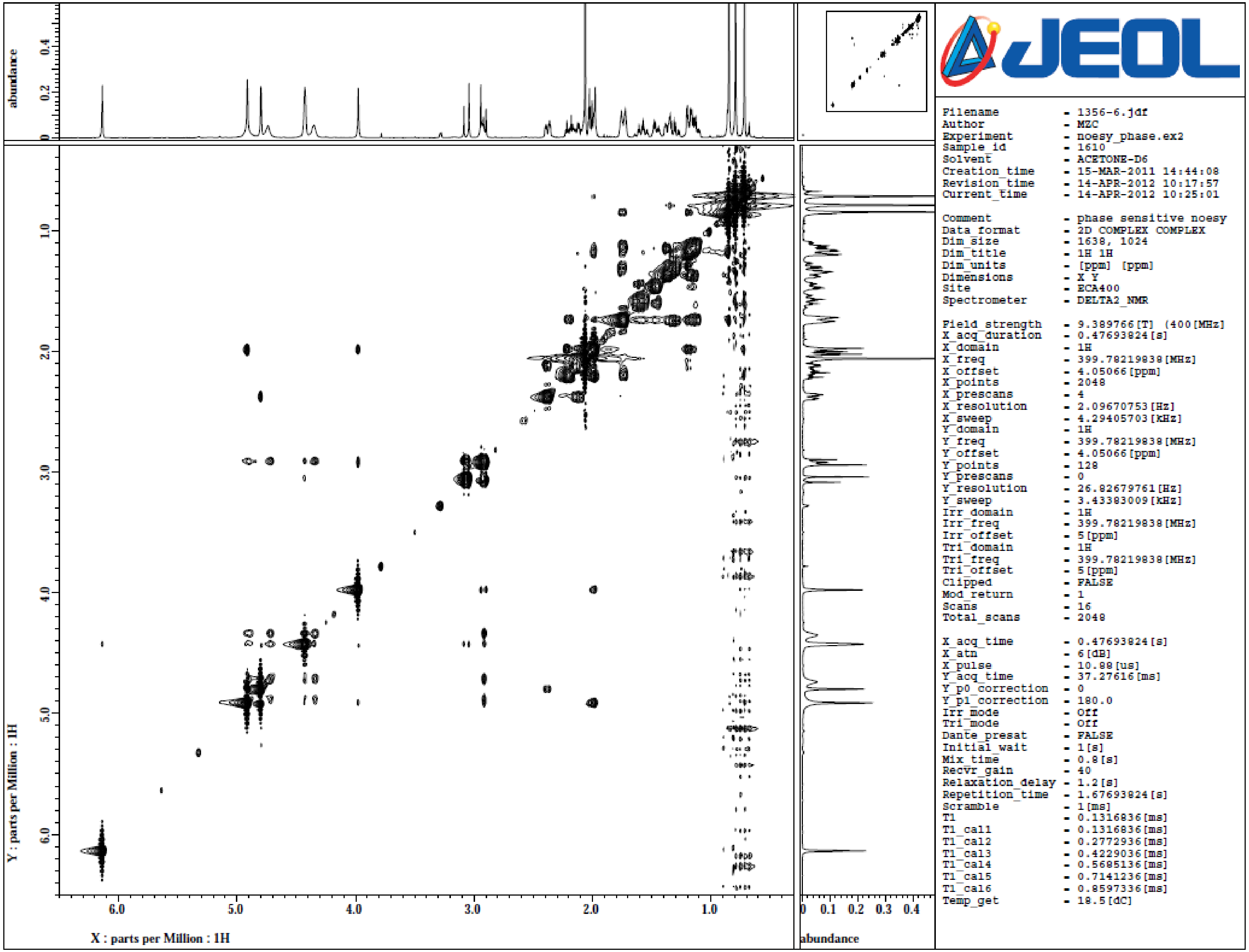

Expanded NOESY Spectrum of **1** in Acetone-d_6

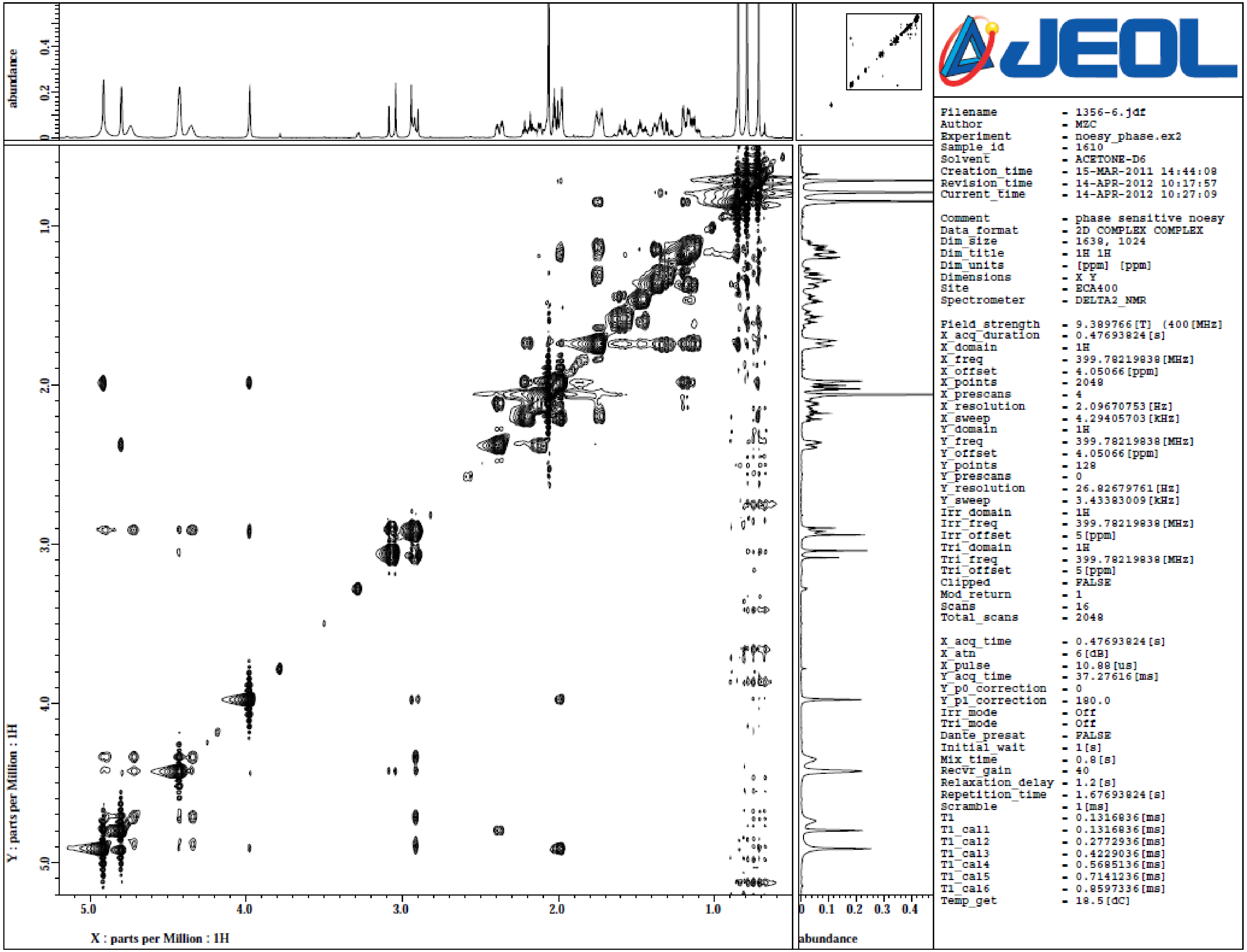

Expanded NOESY Spectrum of **1** in Acetone-d_6

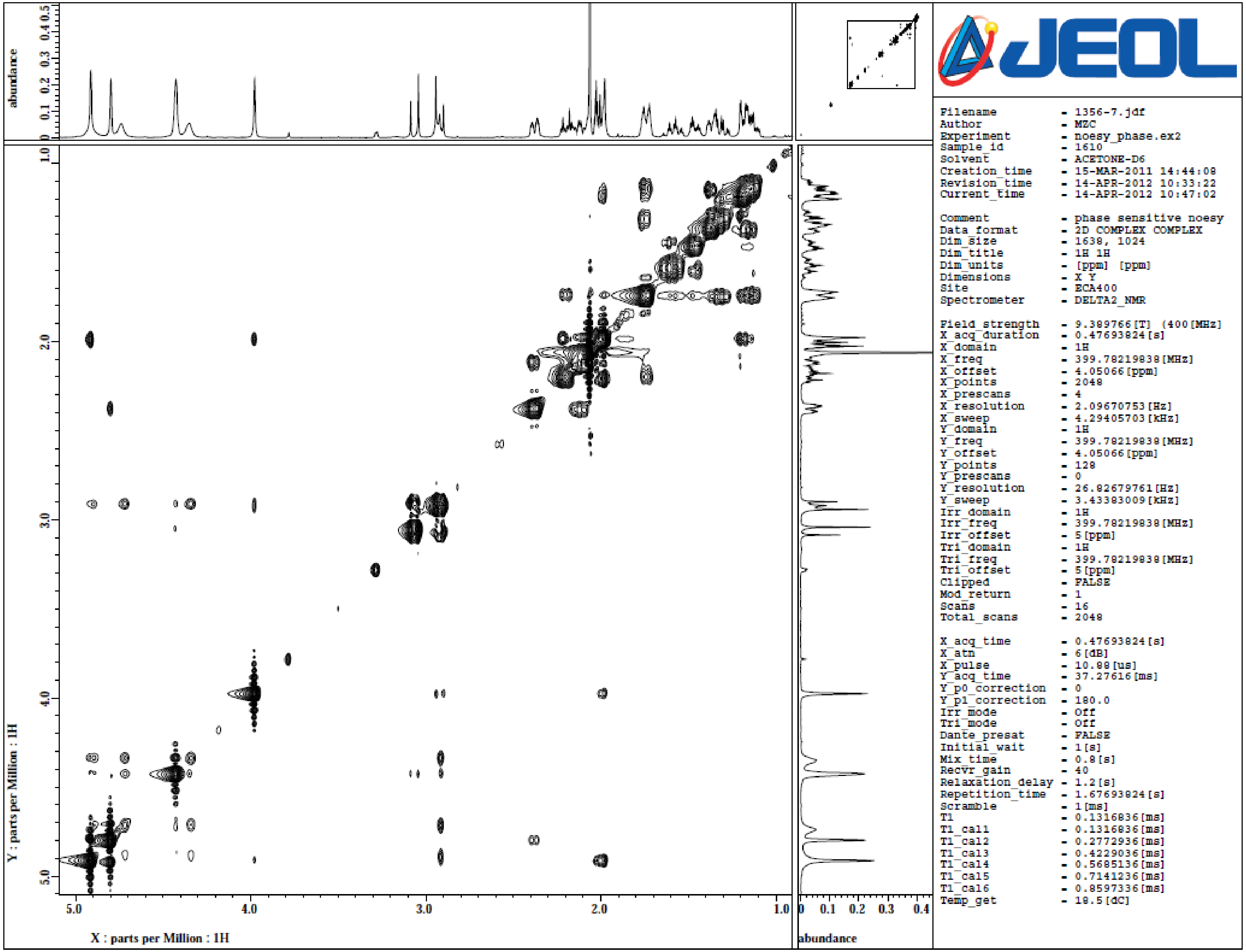

Expanded NOESY Spectrum of **1** in Acetone-d_6

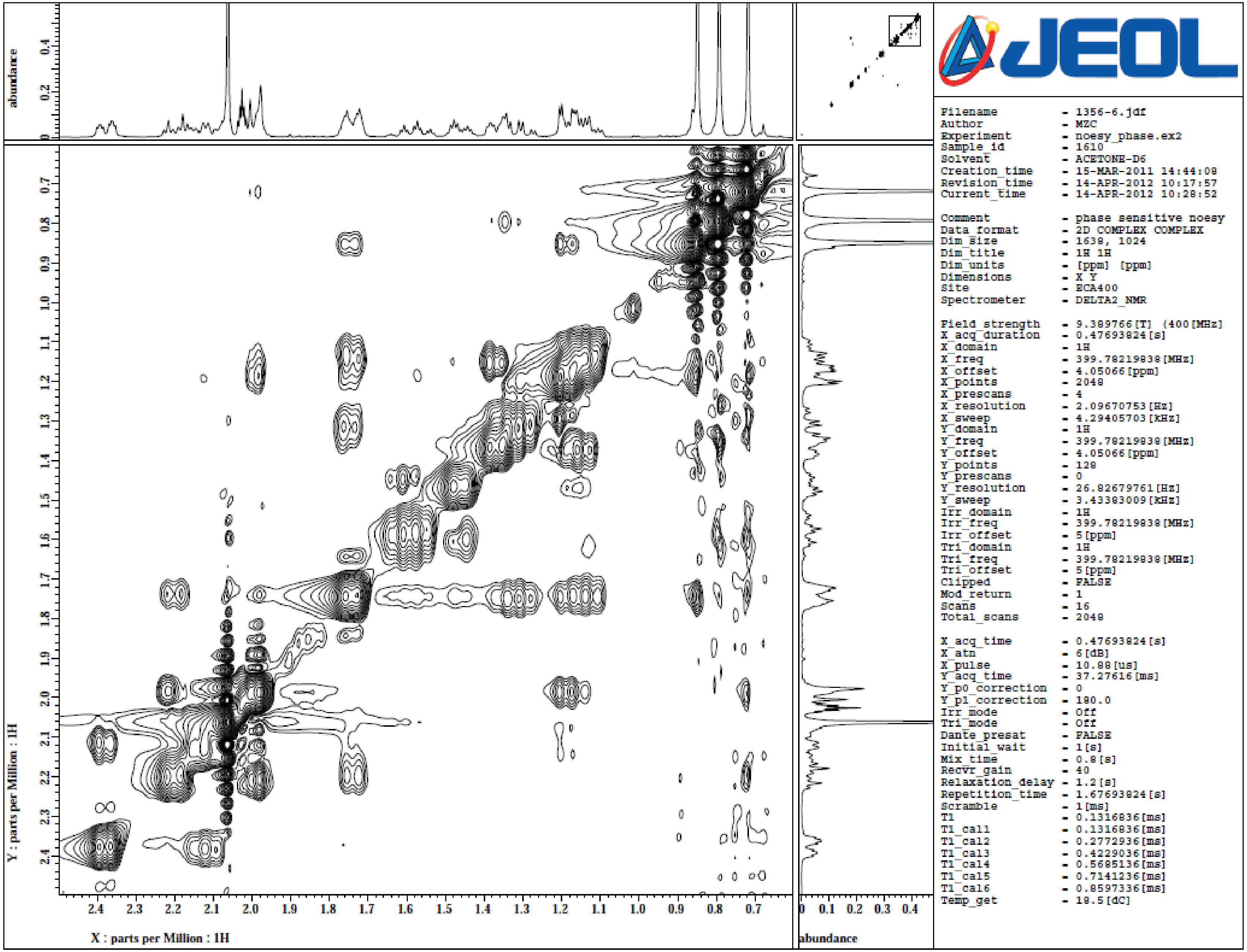

Expanded NOESY Spectrum of **1** in Acetone-d_6

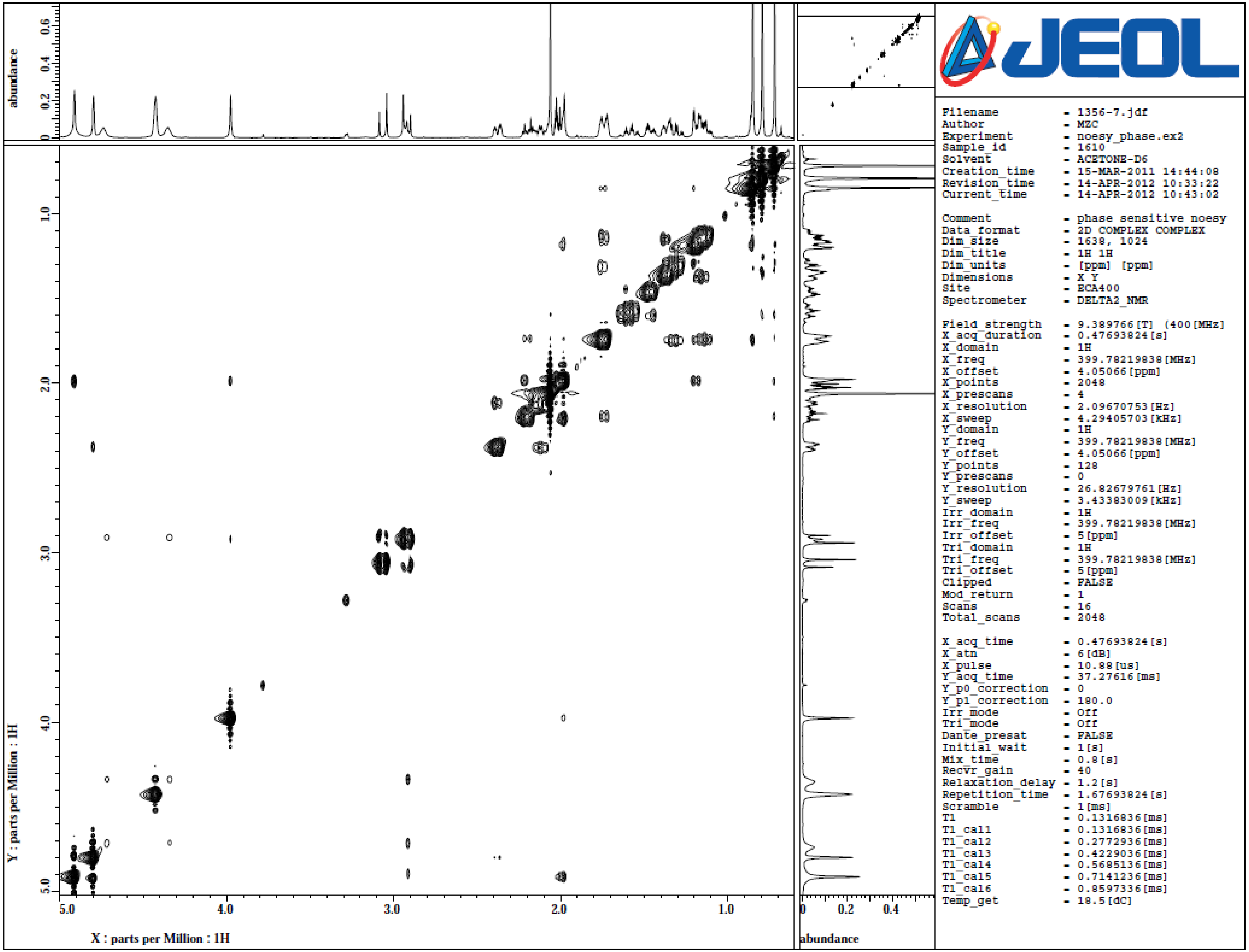

Expanded NOESY Spectrum of **1** in Acetone-d_6

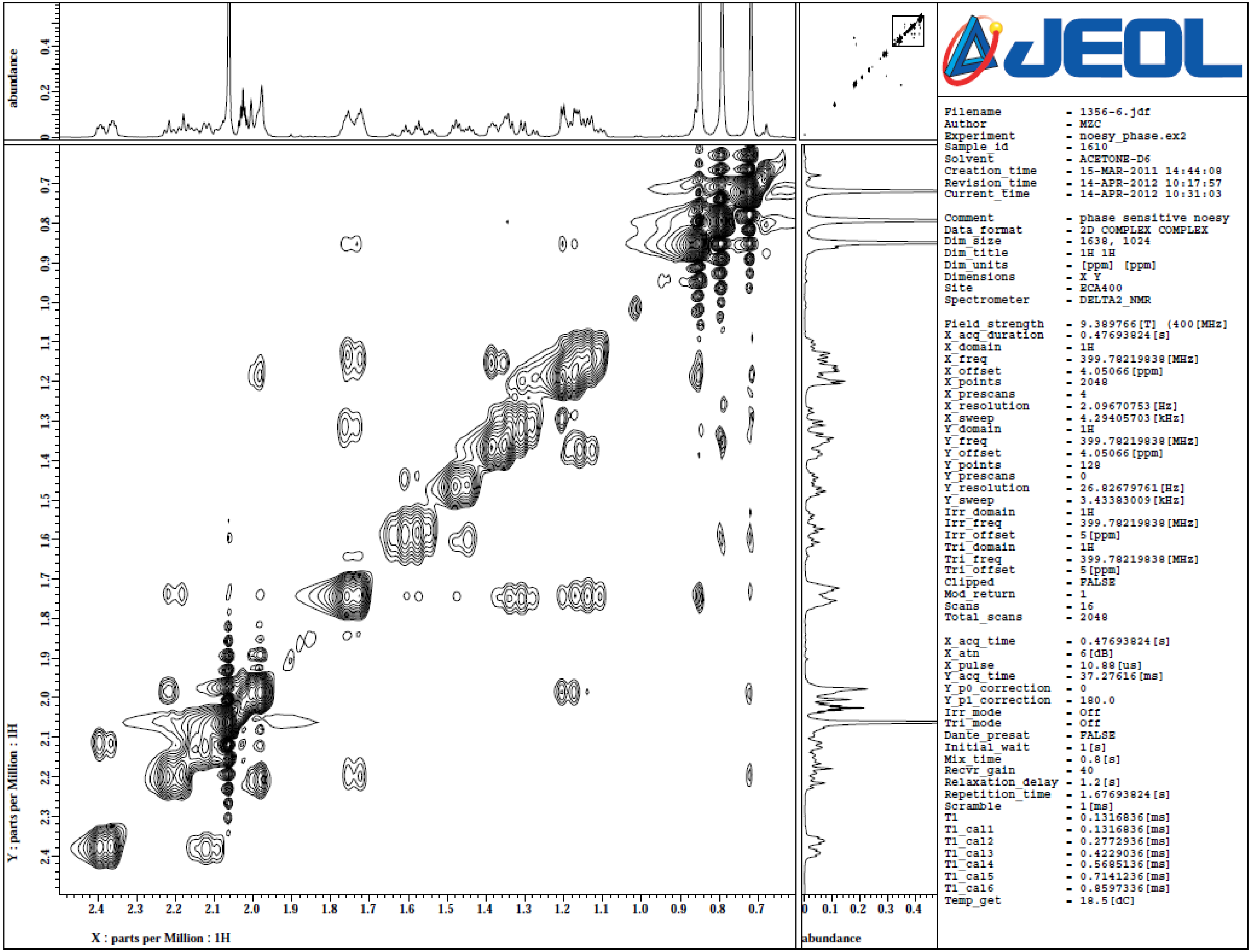

Expanded NOESY Spectrum of **1** in Acetone-d_6

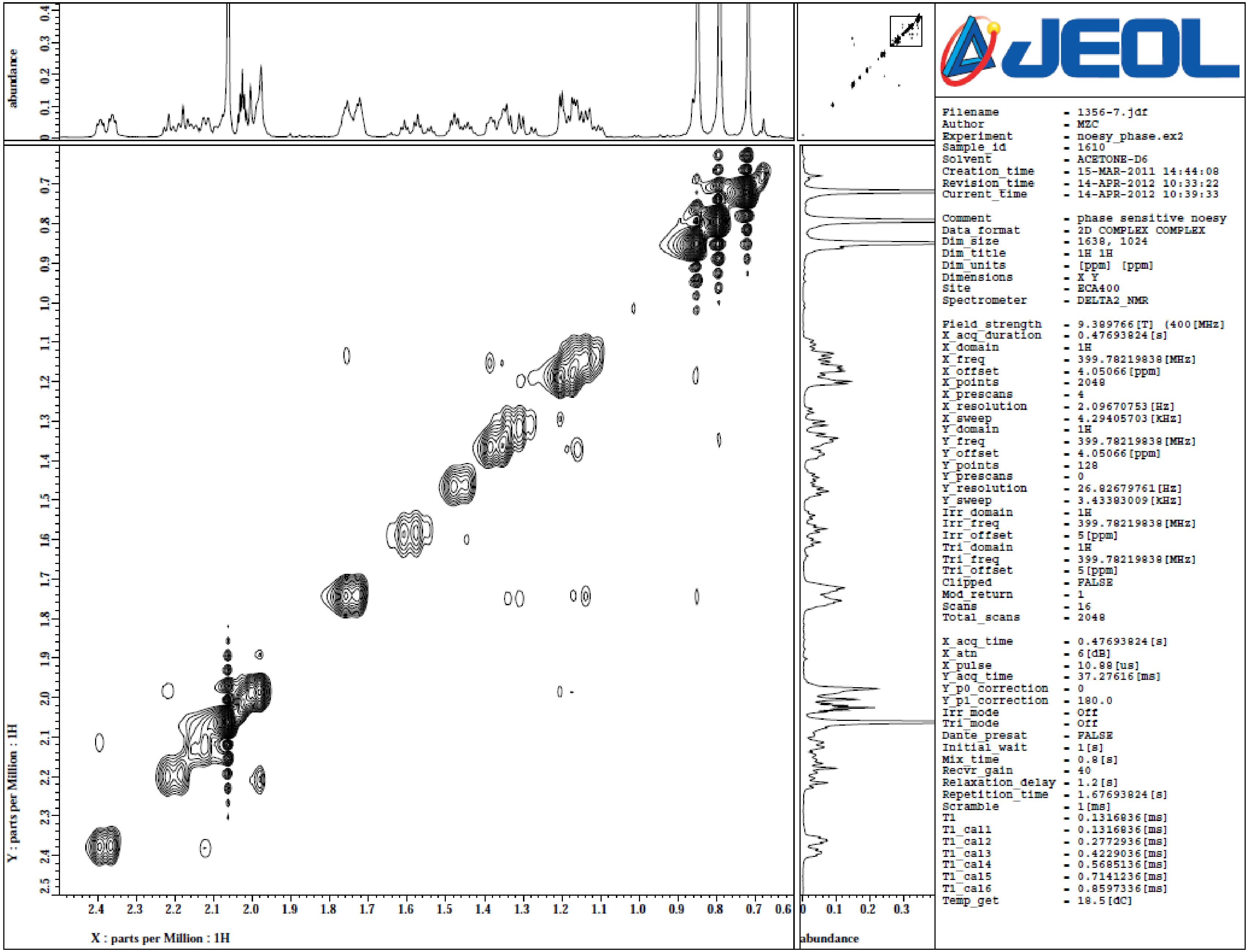

Supplement: Supplementary File 1: — ZIP-Document (ZIP, 8582 KB) [file marinedrugs-10-01266-s001.zip › Supplementary Data S1_MS, UV, IR and NMR spectra of 1.pdf]
