# Supplementary material for: Purpurogemutantin and Purpurogemutantidin, New Drimenyl Cyclohexenone Derivatives Produced by a Mutant Obtained by Diethyl Sulfate Mutagenesis of a Marine-Derived Penicillium purpurogenum G59
Source: Mar Drugs. 2012 Jun 4;10(6):1266–87. doi: 10.3390/md10061266 (PMC3397438; doi:10.3390/md10061266)

Supplementary Materials

Supplementary Data S2. CD and ECD spectra of 1 and 2

Contents

	Content	Page
1.	Computational description for ECD and $[\alpha]_D$ calculations	1
2.	CD spectrum and ECD and $[\alpha]_D$ calculations for **1**	2
3.	CD spectrum and ECD calculations for **2**	3

1. Computational description for ECD and $[\alpha]_D$ calculations

Electronic circular dichroism (ECD) spectra for each of four questionable stereoisomers of **1** and **2** were obtained by the quantum chemical time-dependent density functional theory (TDDFT) [1] calculations using the software package Gaussian 09 [2]. Conformational searches were performed by using the MMFF94S molecular mechanics force field and charged with MMFF94. The resulting conformation were optimized by DFT at the B3LYP/6-31G level. The theoretical ECD calculations on the optimized conformers were performed by using the TDDFT method at B3LYP/6-311++G (2d,p) level. Specific optical rotations were calculated at the B3LYP/6-31G(d) level using B3LYP/6-31+G(d) geometries. The polarizable continuum model (PCM) was adopted to consider solvent effects using the dielectric constant of methanol (ε=32.6) for both ECD and $[\alpha]_D$ calculations.

[1] Bringmann, G.; Bruhn, T.; Maksimenka, K.; Hemberger, Y. The assignment of absolute sterostructures through quantum chemical circular dichroism calculations. *Eur. J. Org. Chem.* **2009**, 2009 (17), 2717–2727.

[2] Frisch, M.J.; Trucks, G.W.; Schlegel, H.B.; Scuseria, G.E.; Robb, M.A.; Cheeseman, J.R.; Scalmani, G.; Barone, V.; Mennucci, B.; Petersson, G.A.; Nakatsuji, H.; Caricato, M.; Li, X.; Hratchian, H.P.; Izmaylov, A.F.; Bloino, J.; Zheng, G.; Sonnenberg, J.L.; Hada, M.; Ehara, M.; Toyota, K.; Fukuda, R.; Hasegawa, J.; Ishida, M.; Nakajima, T.; Honda, Y.; Kitao, O.; Nakai, H.; Vreven, T.; Montgomery Jr., J.A.; Peralta, J.E.; Ogliaro, F.; Bearpark, M.; Heyd, J.J.; Brothers, E.; Kudin, K.N.; Staroverov, V.N.; Kobayashi, R.; Normand, J.; Raghavachari, K.; Rendell, A.; Burant, J.C.; Iyengar, S.S.; Tomasi, J.; Cossi, M.; Rega, N.; Millam, J.M.; Klene, M.; Knox, J.E.; Cross, J.B.; Bakken, V.; Adamo, C.; Jaramillo, J.; Gomperts, R.; Stratmann, R.E.; Yazyev, O.; Austin, A.J.; Cammi, R.; Pomelli, C.; Ochterski, J.W.; Martin, R.L.; Morokuma, K.; Zakrzewski, V.G.; Voth, G.A.; Salvador, P.; Dannenberg, J.J.; Dapprich, S.; Daniels, A.D.; Farkas, O.; Foresman, J.B.; Ortiz, J.V.; Cioslowski, J.; Fox, D.J. *Gaussian 09, Revision A.02*. **2009**, Gaussian Inc., Wallingford CT.

2. CD spectrum and ECD and [α]$_D$ calculations for **1**

Spectroscopic studies on the structure of **1** suggested four possible stereoisomers belonging to two pairs of enantiomers with absolute configurations, 5*S*9*S*10*S*4'*R*5'*R*6'*S* (**A1**) and 5*R*9*R*10*R*4'*S*5'*S*6'*R* (**A2**), and 5*S*9*S*10*S*4'*S*5'*S*6'*R* (**B1**) and 5*R*9*R*10*R*4'*R*5'*R*6'*S* (**B2**), respectively, as shown in Figure S2-1. Quantum chemical calculations were performed on all four possible stereoisomers, **A1**, **A2**, **B1**, and **B2**, to obtain their ECD spectra. A systematic conformational analysis was carried out using the MMFF94 molecular mechanics force field and charged with MMFF94. The MMFF94 conformers obtained were further optimized and verified stability at the B3LYP/6-31g(d) level. The polarizable continuum model (PCM) was adopted to consider solvent effects using the dielectric constant of methanol (ε=32.6). The 30 lowest electronic transitions were calculated, and rotational strengths for each electronic excitation were given using both dipole length and dipole velocity representations. ECD spectra of different conformers were simulated using a Gaussian function with a half-bandwidth of 0.3 eV. Equilibrium populations of conformers at 298.15 K were calculated from their relative free energies (ΔG) using Boltzmann statistics, as shown for **A1** and **B1** in Figures S2-2 and S2-3, and finally, the overall ECD spectra were then generated according to the Boltzmann weighting of each conformer.

Figure S2-1. Structures of four possible stereoisomers of **1**.

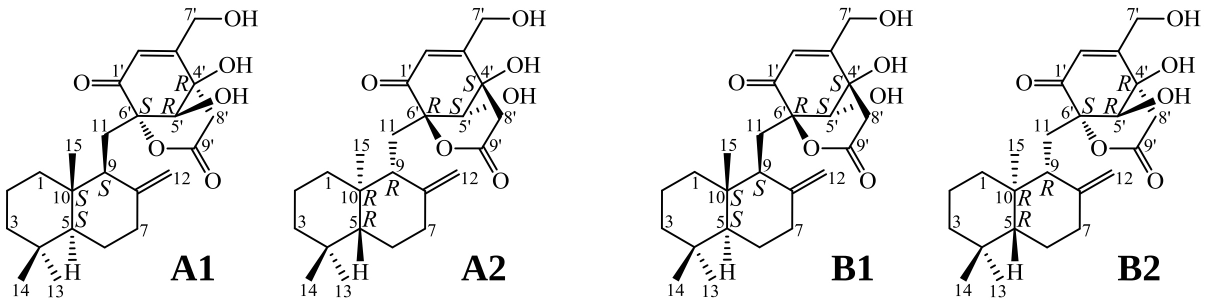

Figure S2-2. Optimized three conformers of **A1** and their equilibrium populations.

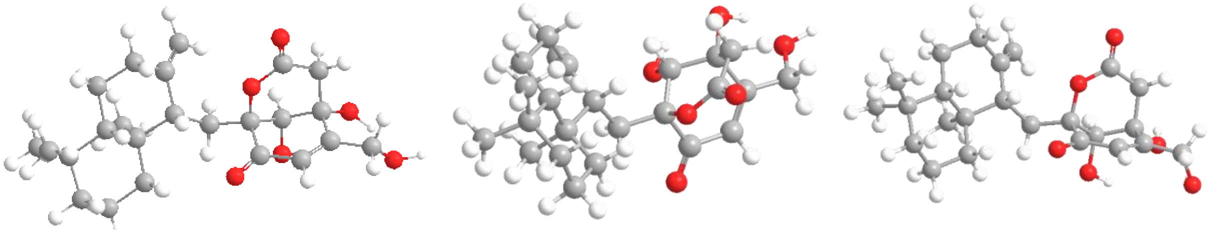

A1a (9.99%) **A1b (88.64%)** **A1c (1.37%)**

Figure S2-3. Optimized two conformers of **B1** and their equilibrium populations.

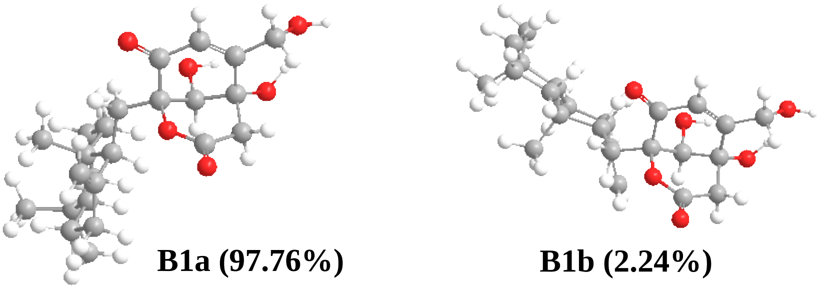

B1a (97.76%) **B1b (2.24%)**

Among the ECD spectra calculated (Figure S2-4), ECD spectrum of the stereoisomer **A1** best matched the experimental CD spectrum of **1**. However, it was insufficiently assigned straightforwardly to **1** only according to the ECD data because the stereoisomer **B2** with the opposite configuration of drimenyl residue also reproduced properly the CD spectrum of **1**. A quite large difference in the specific optical rotations was readily expected between two diastereoisomers **A1** and **B2**. We therefore carried out theoretical calculation of the specific optical rotations of **A1** and **B2** at the B3LYP/6-31G(d) level using B3LYP/6-31+G(d) geometries. This has afforded the calculated $[\alpha]_{\mathrm{D}}^{20}$ +15.1 for **A1** and $[\alpha]_{\mathrm{D}}^{20}$ +42.8 for **B2** both in MeOH, respectively. The calculated specific optical rotation of **A1** much better matched the measured $[\alpha]_{\mathrm{D}}^{20}$ +21.0 (1.0, MeOH) of **1**. Thus, the absolute configuration of **1** could be assigned as 5*S*9*S*10*S*4'*R*5'*R*6'*S*.

Figure S2-4. Measured CD spectrum and calculated ECD spectra for **1** in MeOH.

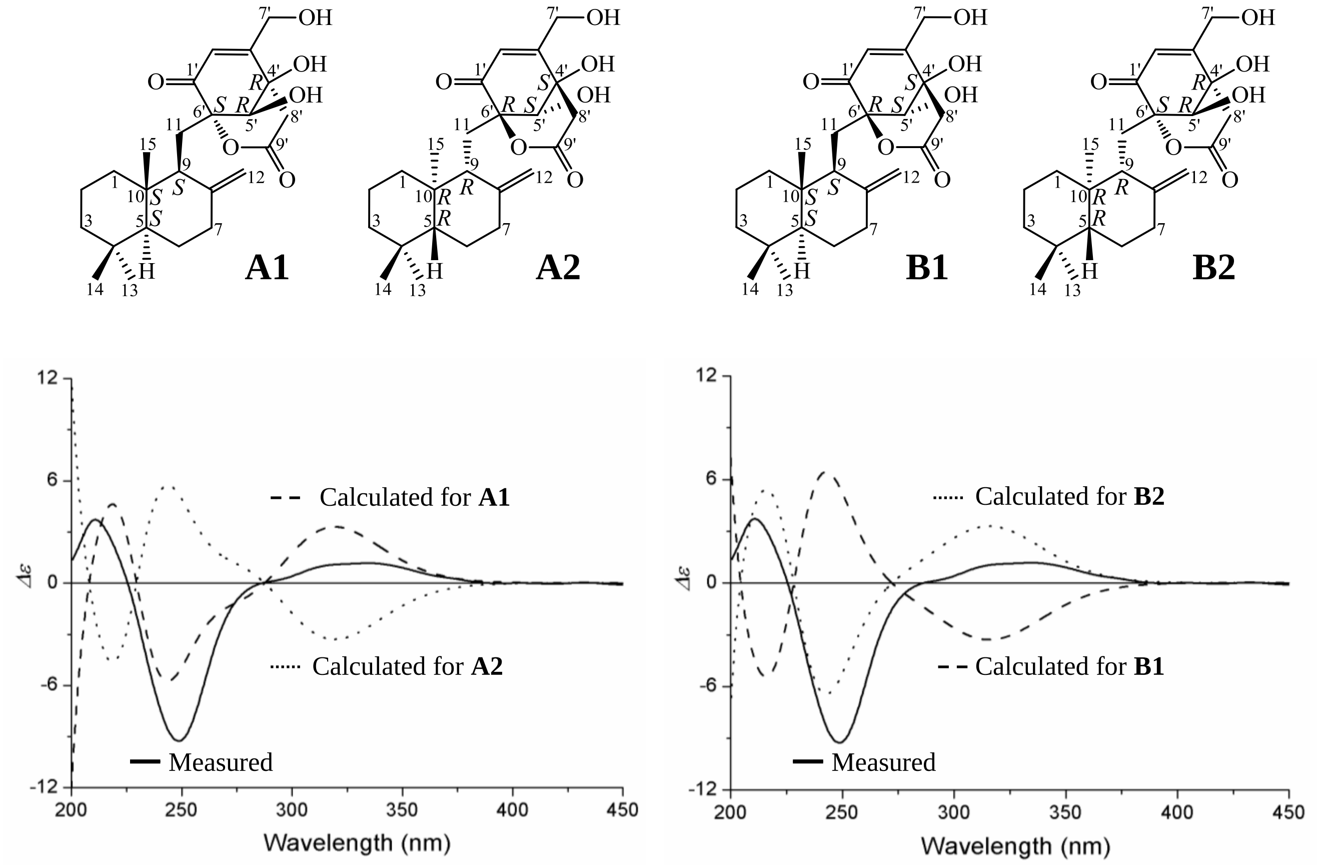

3. CD spectrum and ECD calculations for **2**

Spectroscopic studies on the chemical structure of **2** suggested four possible stereoisomers belonging to two pairs of enantiomers with absolute configurations, 5*S*9*S*10*S*6'*S* and 5*S*9*S*10*S*6'*R*, and 5*R*9*R*10*R*6'*S* and 5*R*9*R*10*R*6'*R*, respectively. Quantum chemical ECD calculations on the all four questionable isomers were also carried out to obtain their ECD spectra as mentioned for **1**. Among the ECD spectra obtained (Figure S2-5), ECD spectrum of the stereoisomer with 5*S*9*S*10*S*6'*S* absolute stereochemistry matched well the experimental CD spectrum of **2** (Figure S2-5). Thus the absolute configuration of **2** was determined as 5*S*9*S*10*S*6'*S*. The calculated ECD spectra and their comparisons with the experimental CD spectrum of **2** in MeOH are given in Figure S2-5.

Figure S2-5. Measured CD spectrum and calculated ECD spectra for **2** in MeOH.

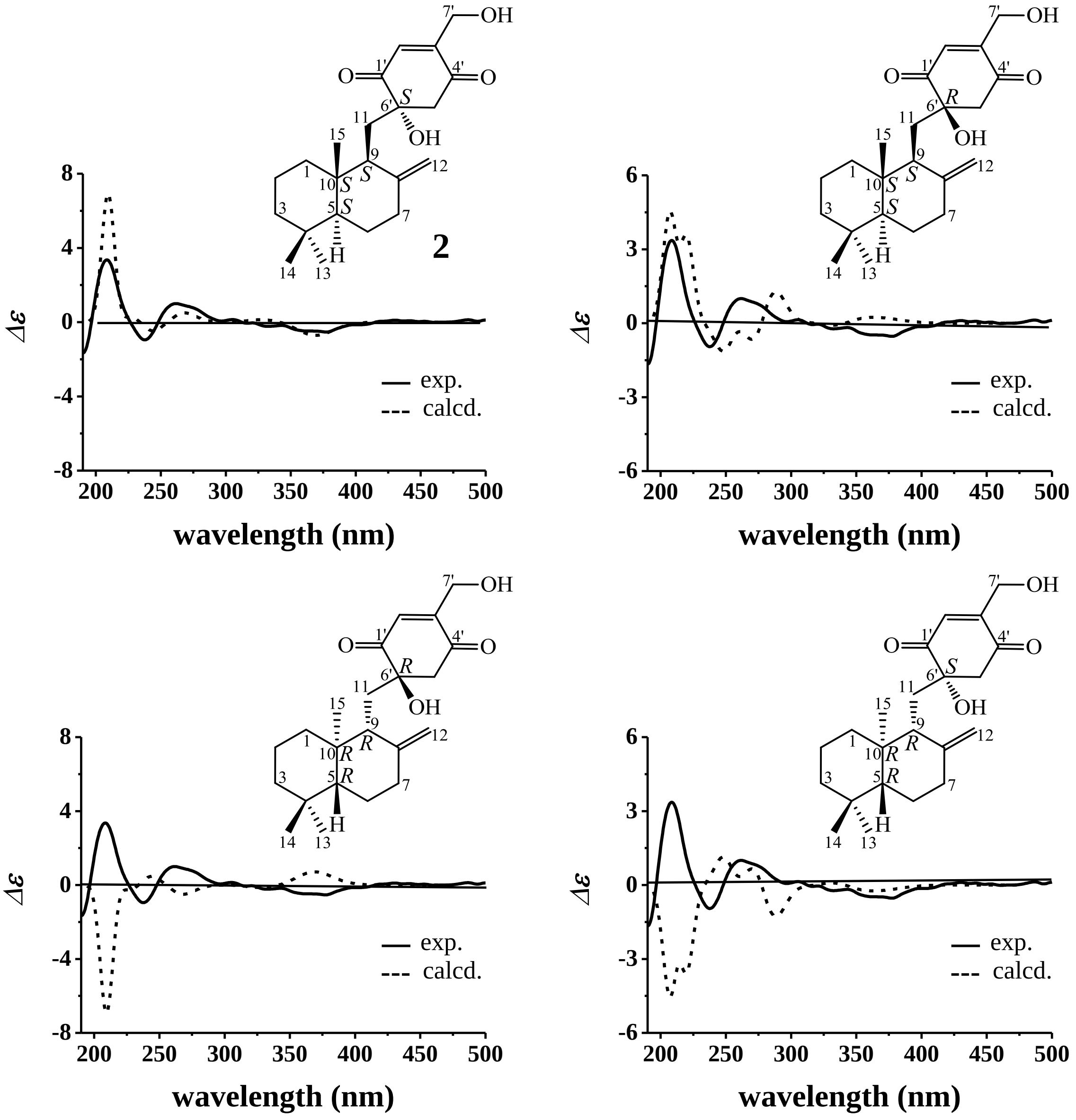

Supplement: Supplementary File 1: — ZIP-Document (ZIP, 8582 KB) [file marinedrugs-10-01266-s001.zip › Supplementary Data S2_CD and ECD spectra of 1 and 2.pdf]
