# Supplementary material for: Purpurogemutantin and Purpurogemutantidin, New Drimenyl Cyclohexenone Derivatives Produced by a Mutant Obtained by Diethyl Sulfate Mutagenesis of a Marine-Derived Penicillium purpurogenum G59
Source: Mar Drugs. 2012 Jun 4;10(6):1266–87. doi: 10.3390/md10061266 (PMC3397438; doi:10.3390/md10061266)

Supplementary Materials

Supplementary Data S3. MS, UV, IR and NMR spectra of 2

Contents

	Content	Page
1.	Positive and negative ESIMS spectra of **2**	2
2.	Positive HRESIMS spectrum of **2**	3
3.	Negative HRESIMS spectrum of **2**	4
4.	UV spectrum of **2**	5
5.	IR spectrum of **2**	6
6.	^{1}H NMR spectrum of **2**	7
7.	^{13}C NMR and DEPT spectra of **2**	8
8.	^{1}H-^{1}H COSY spectrum of **2**	10
9.	HMQC spectrum of **2**	14
10.	HMBC spectrum of **2**	16
11.	NOESY spectrum of **2**	19

ESIMS and HRESIMS Spectra of **2**

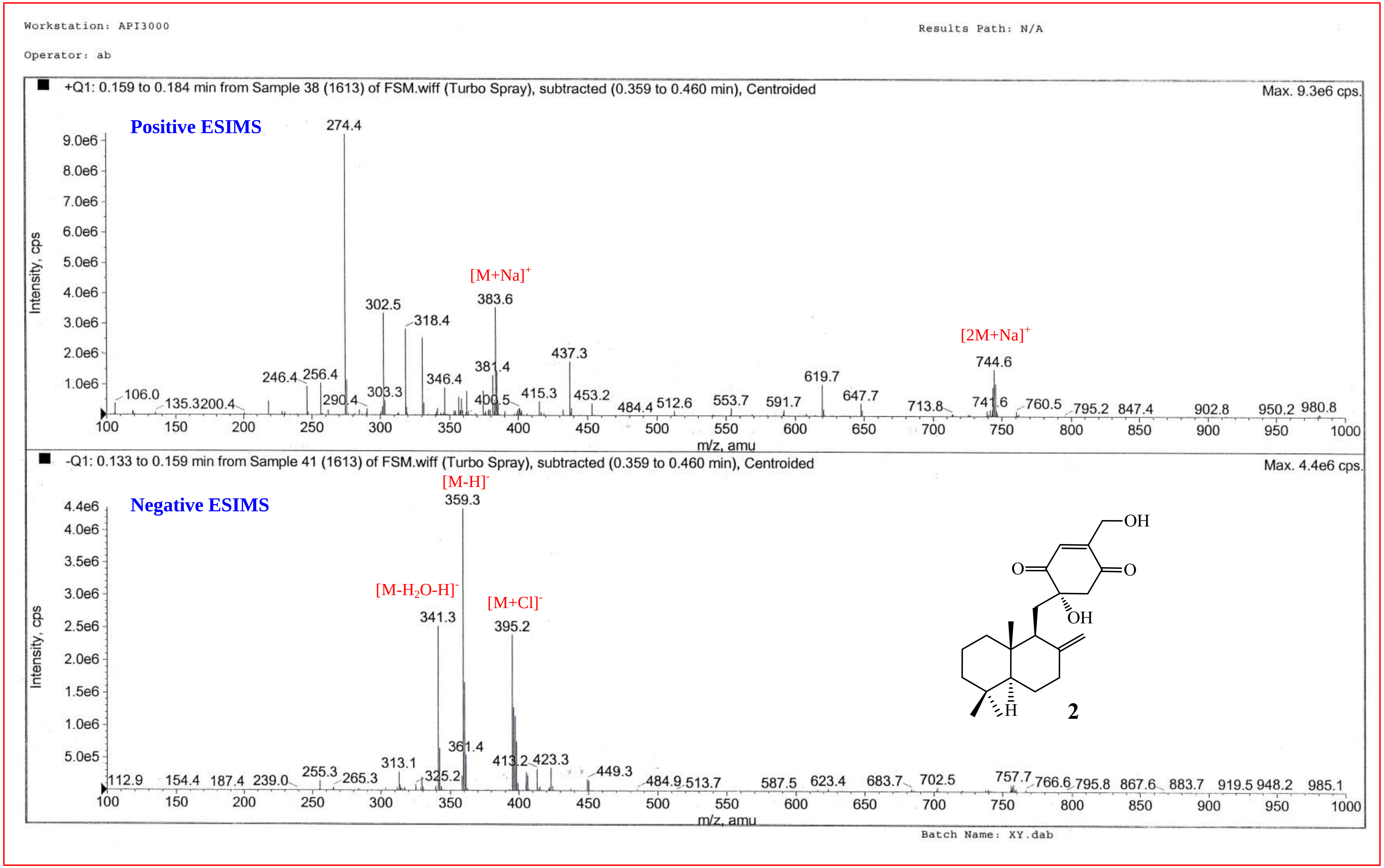

Positive HRESIMS Of 1613

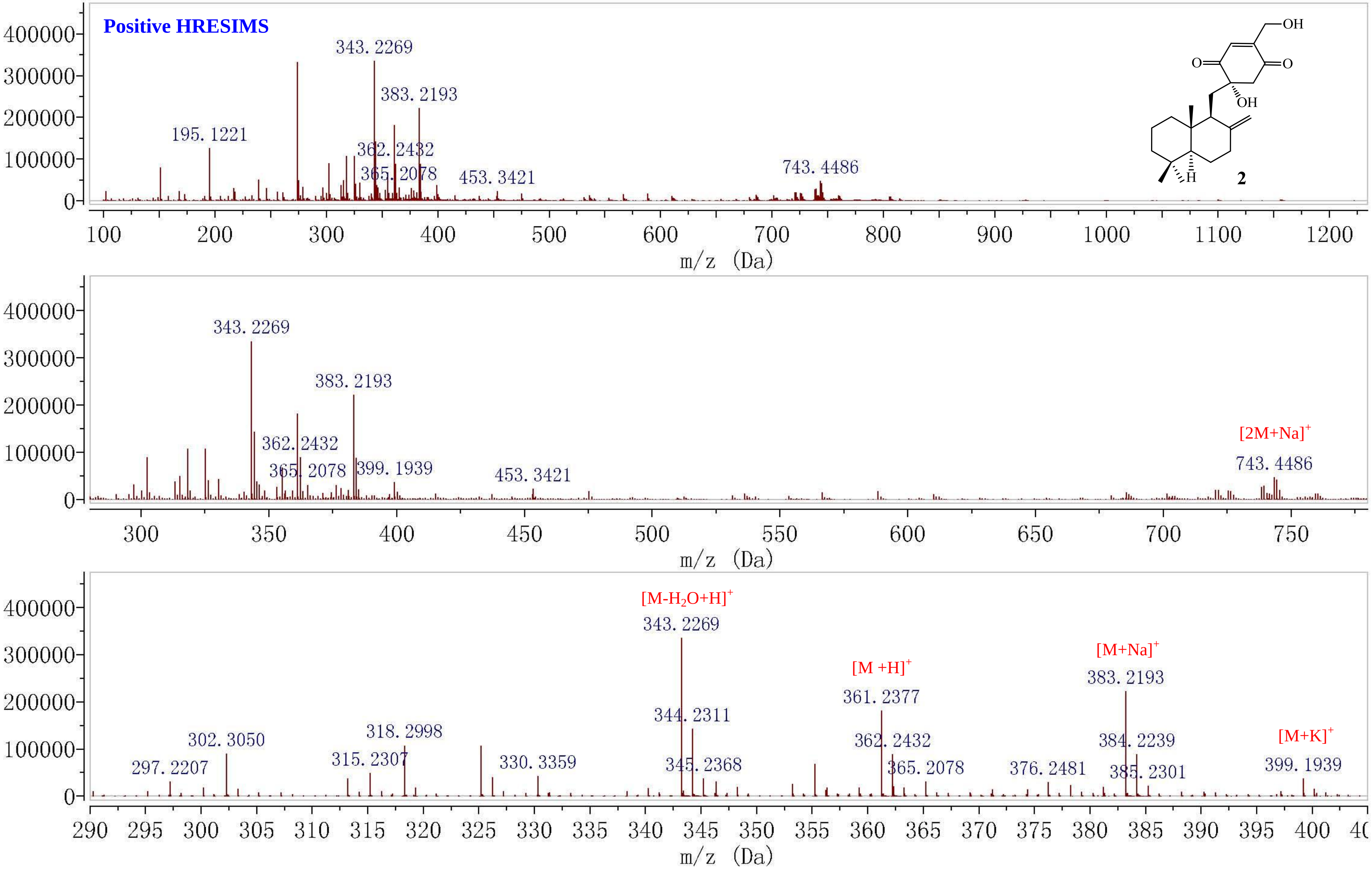

Total Ion Chormatogram (TIC) of Negative HRESIMS of 1613

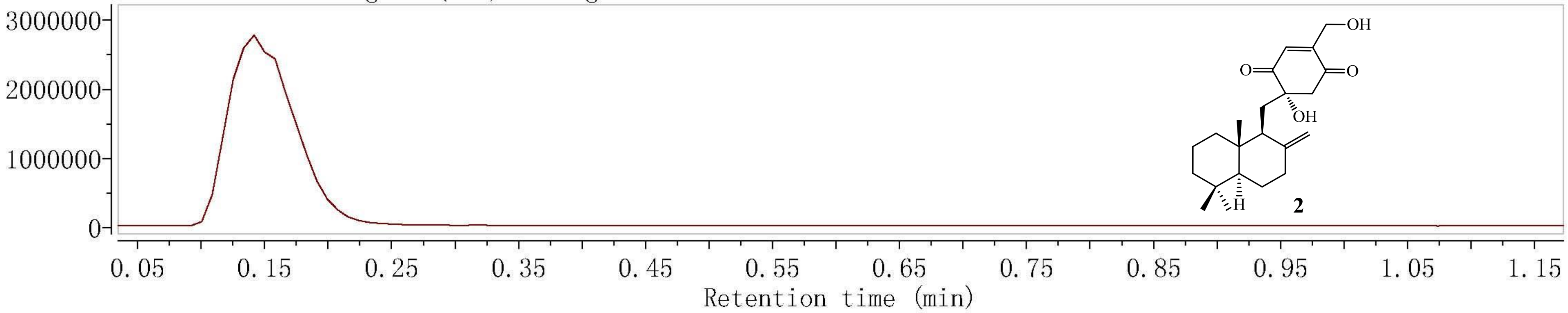

Negative HRESIMS of 1613 at the retention time of 0.180840-0.109232 min in TIC

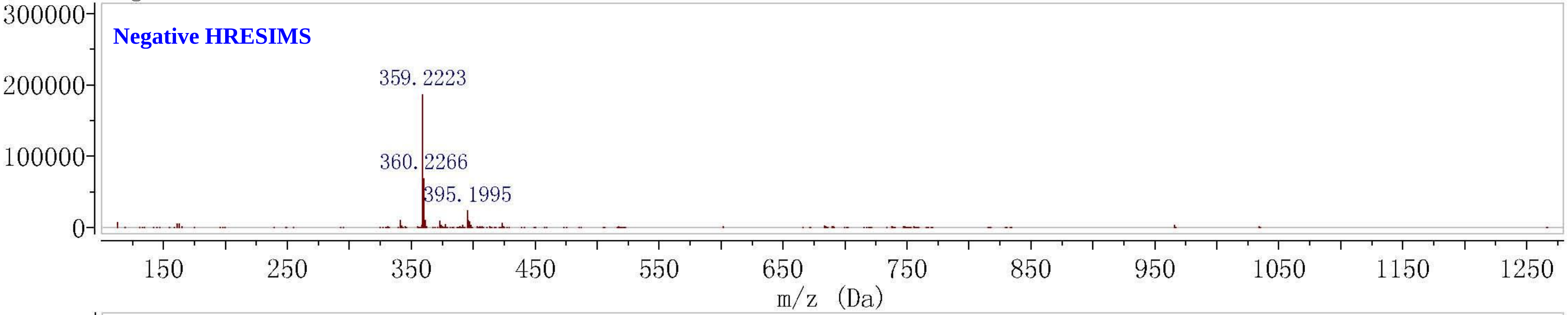

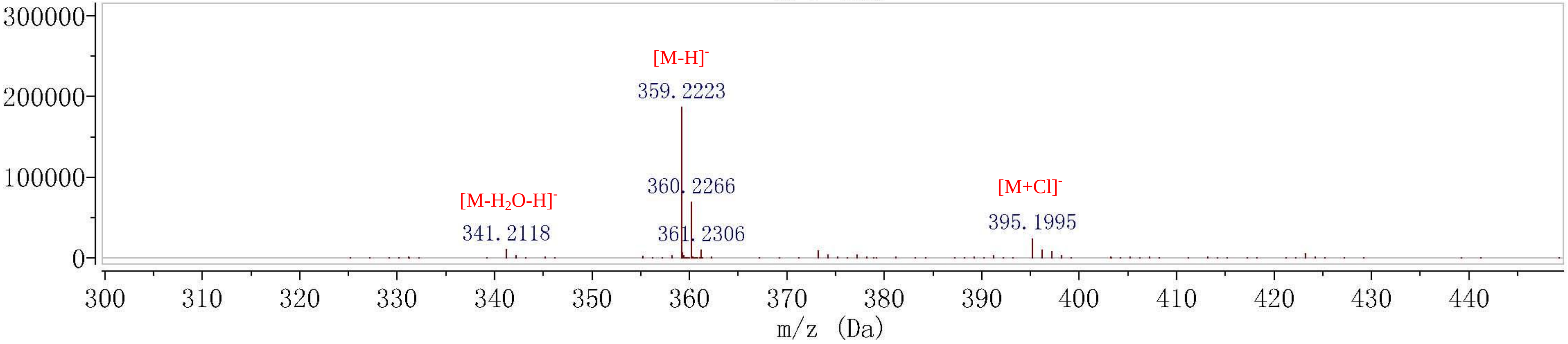

UV Spectrum of **2** in MeOH

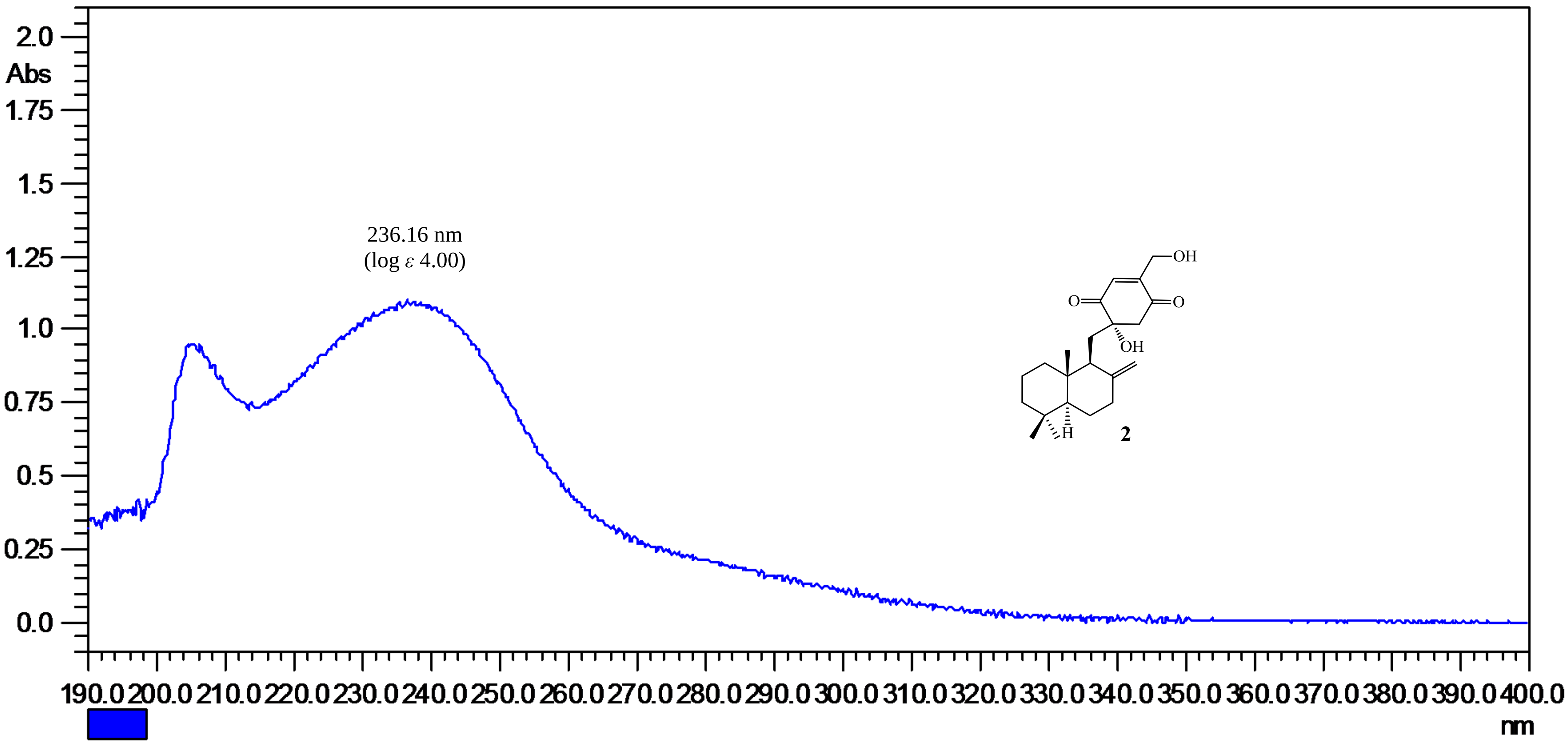

IR Spectrum of **2**

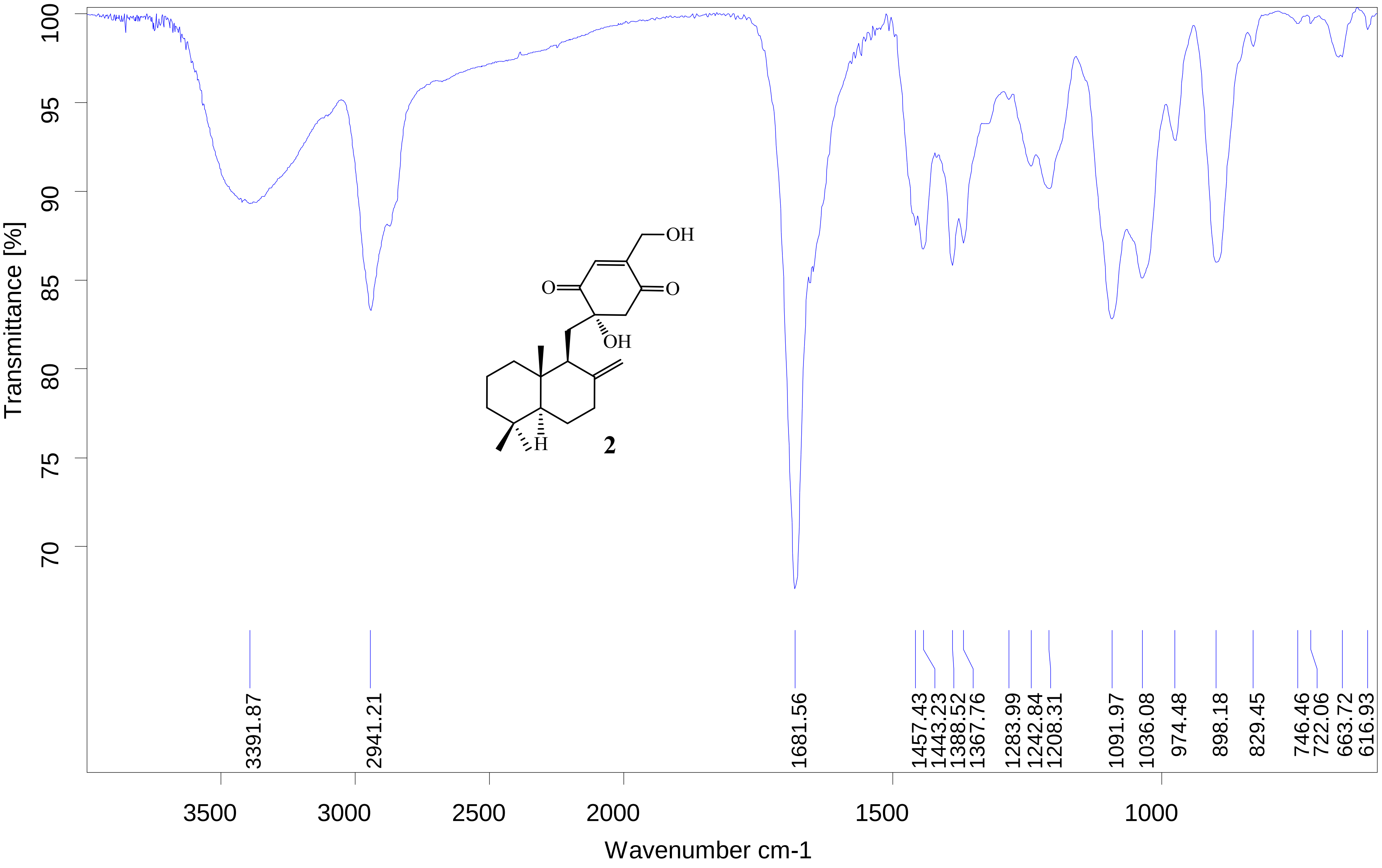

400 MHz ^1H NMR Spectrum of **2** in $CDCl_3$

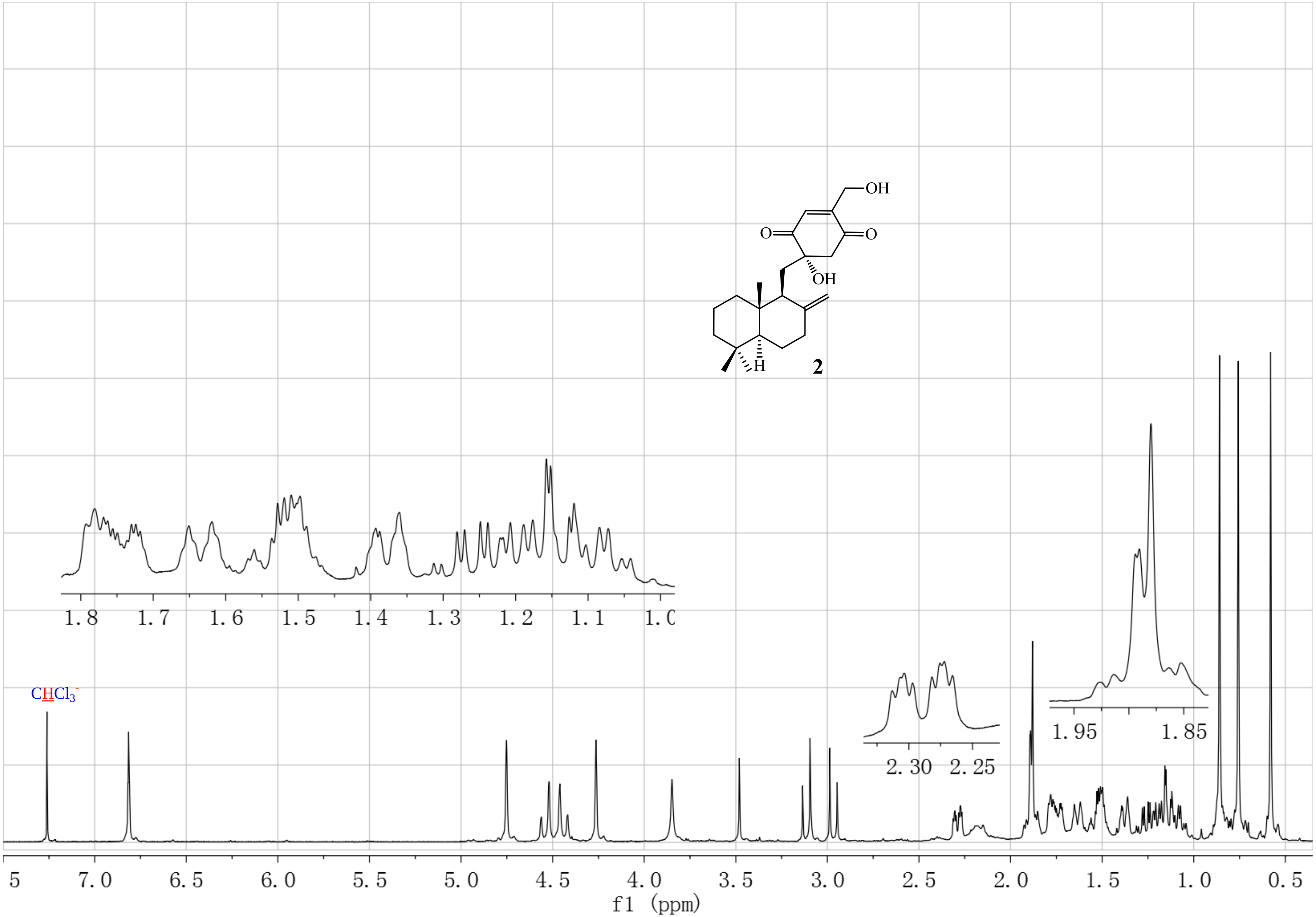

100 MHz ^{13}C NMR Spectrum of **2** in $CDCl_3$

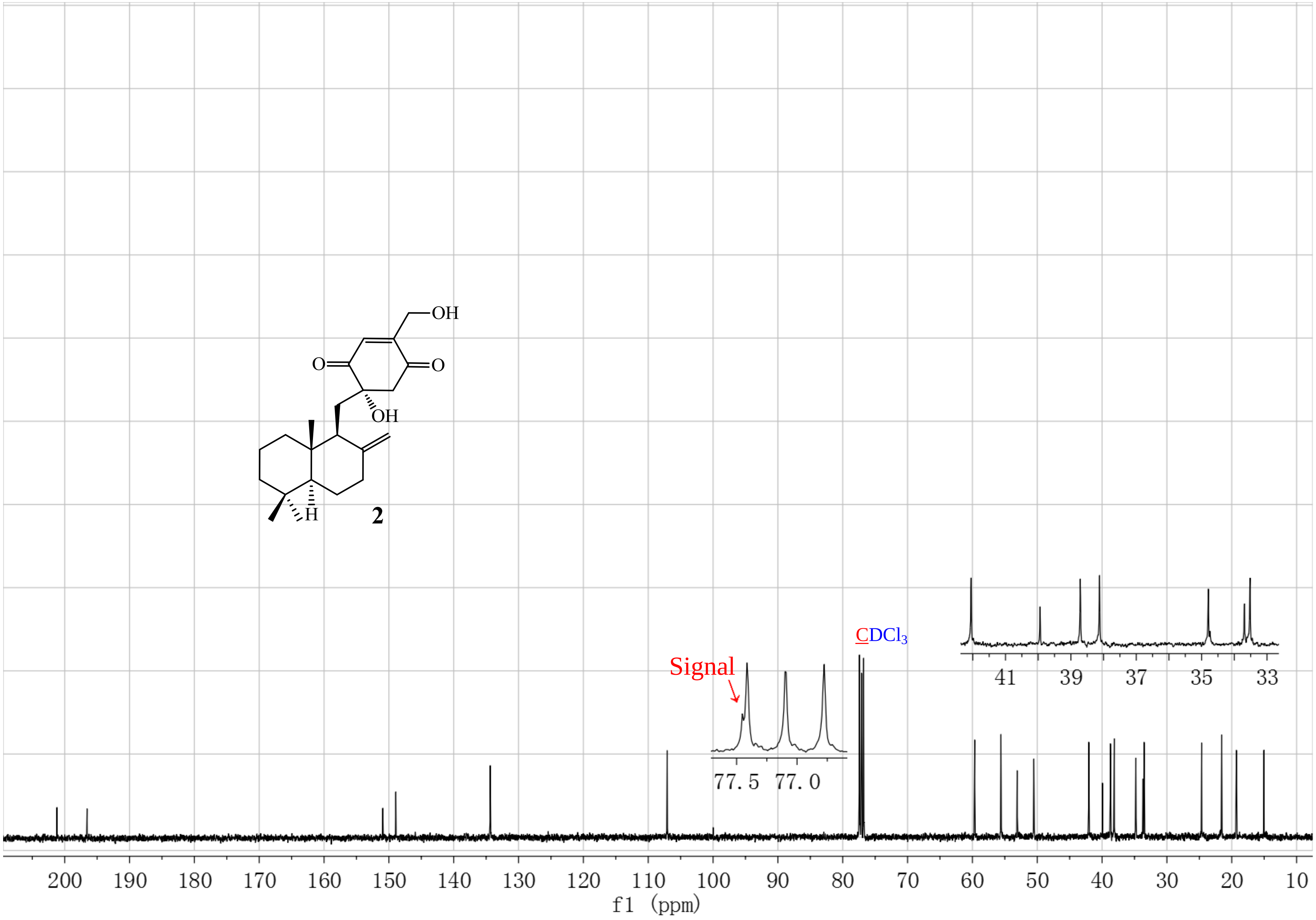

100 MHz ^{13}C NMR and DEPT Spectra of **2** in $CDCl_3$

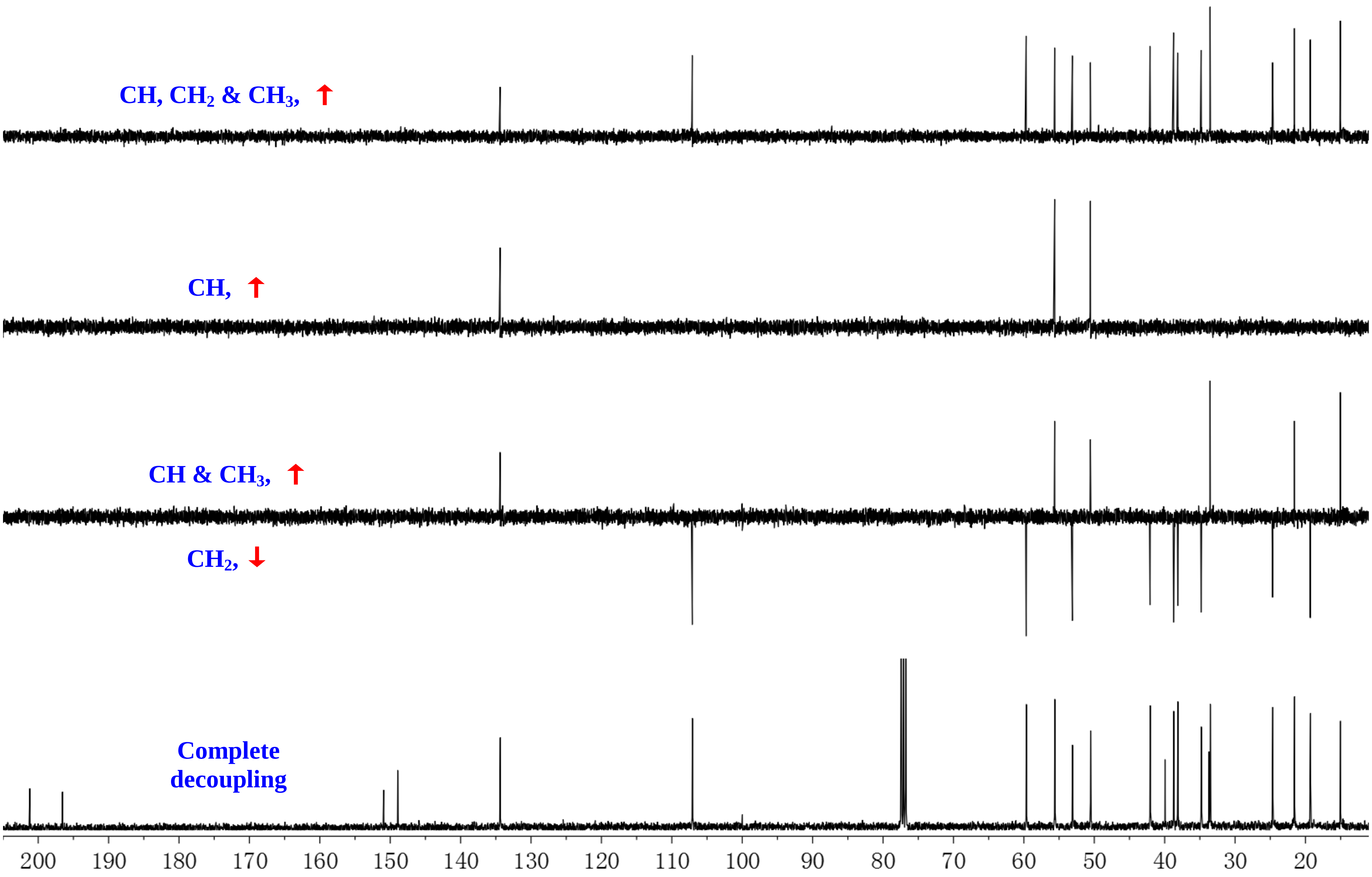

^{1}H-^{1}H COSY Spectrum of **2** in $CDCl_3$

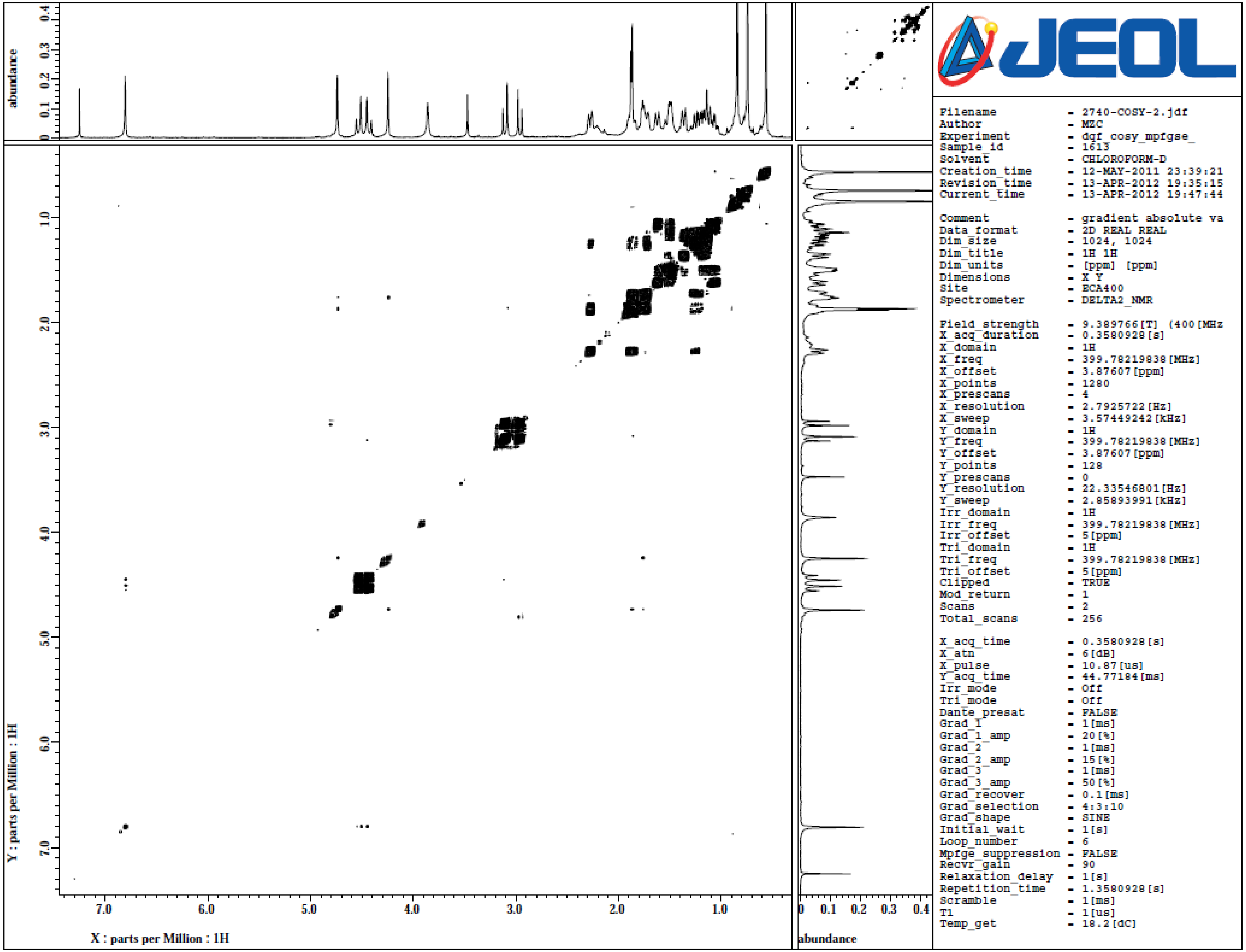

Expanded ^1H-^1H COSY Spectrum of **2** in $CDCl_3$

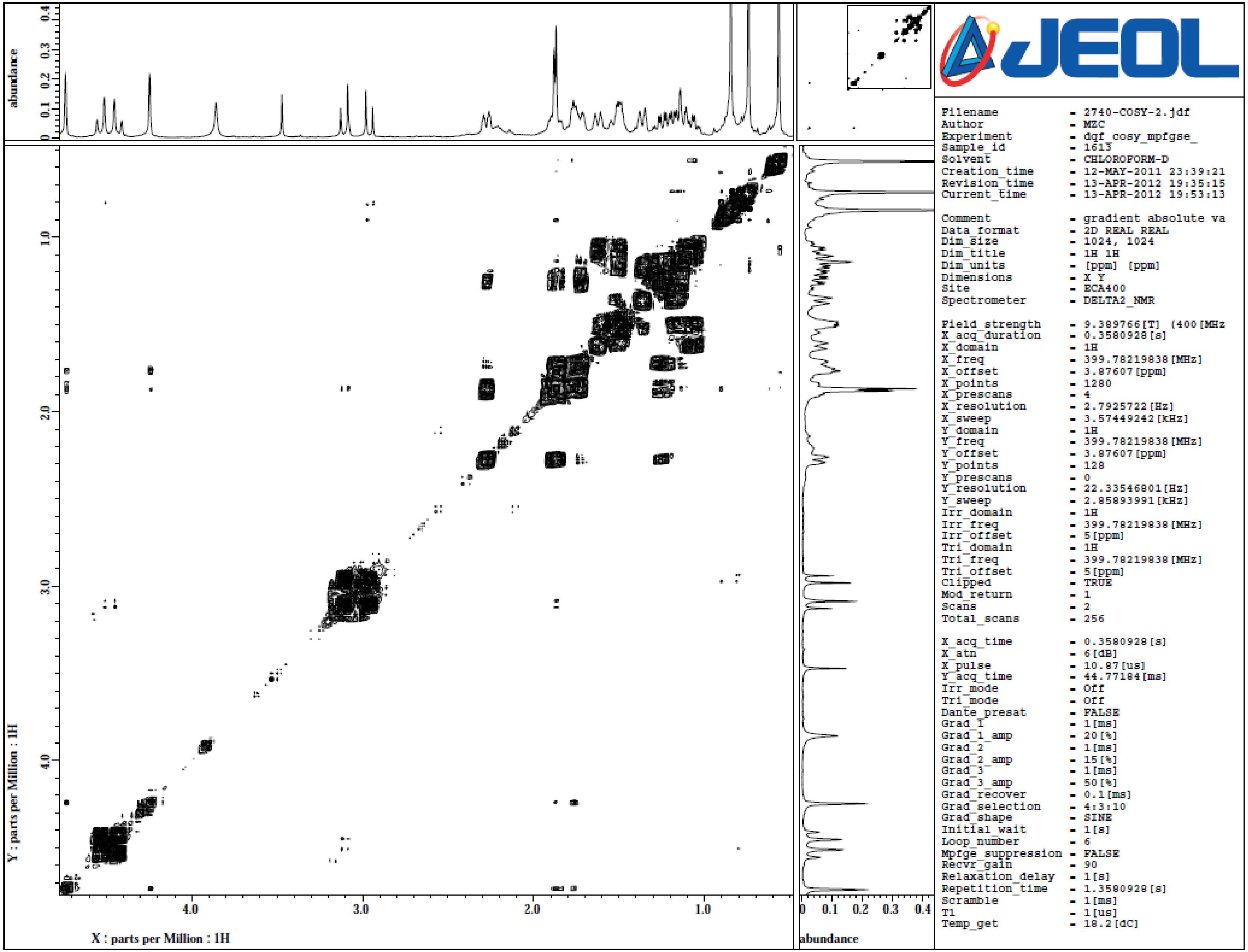

Expanded ^{1}H-^{1}H COSY Spectrum of **2** in $CDCl_3$

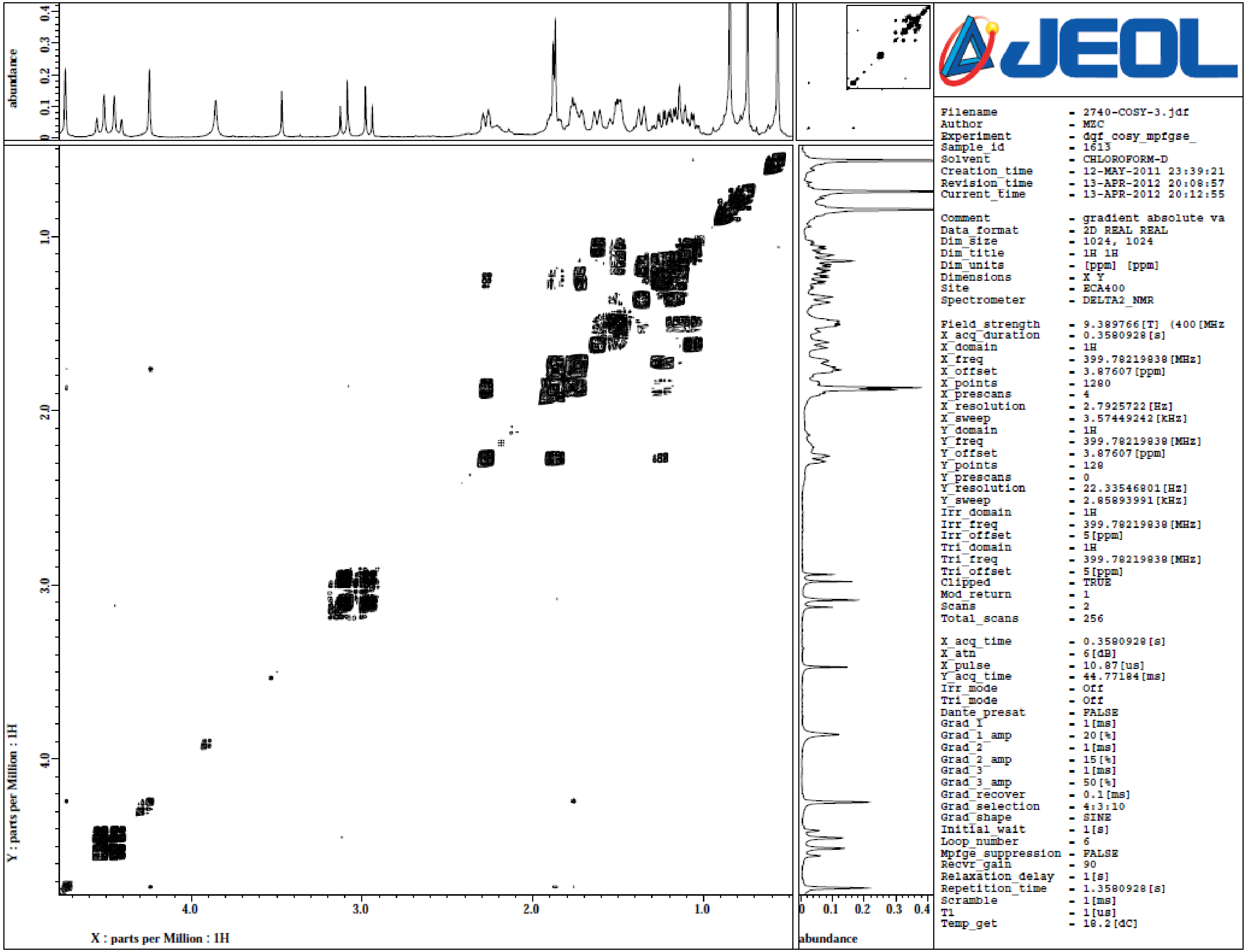

Expanded ^{1}H-^{1}H COSY Spectrum of **2** in $CDCl_3$

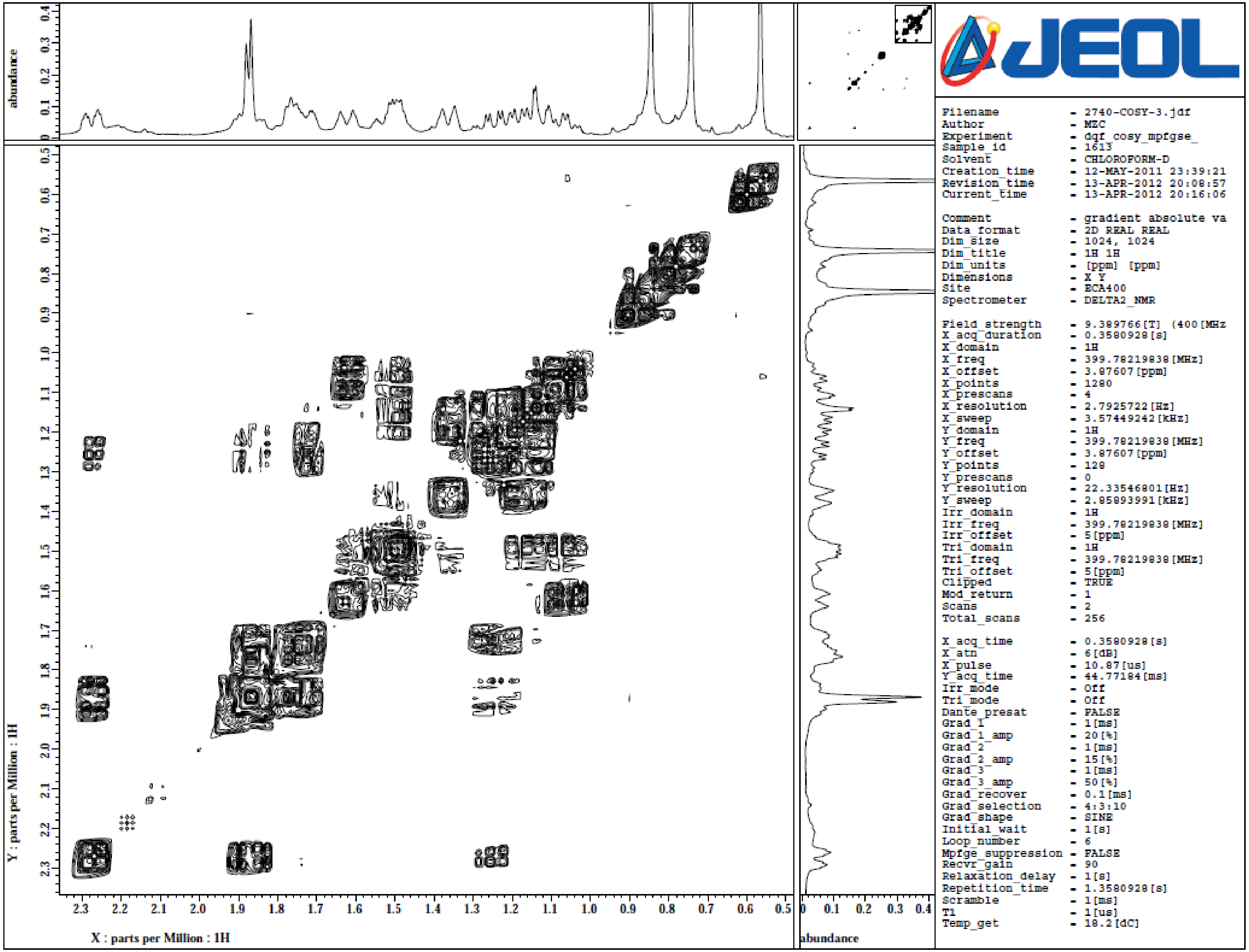

HMQC Spectrum of **2** in $CDCl_3$

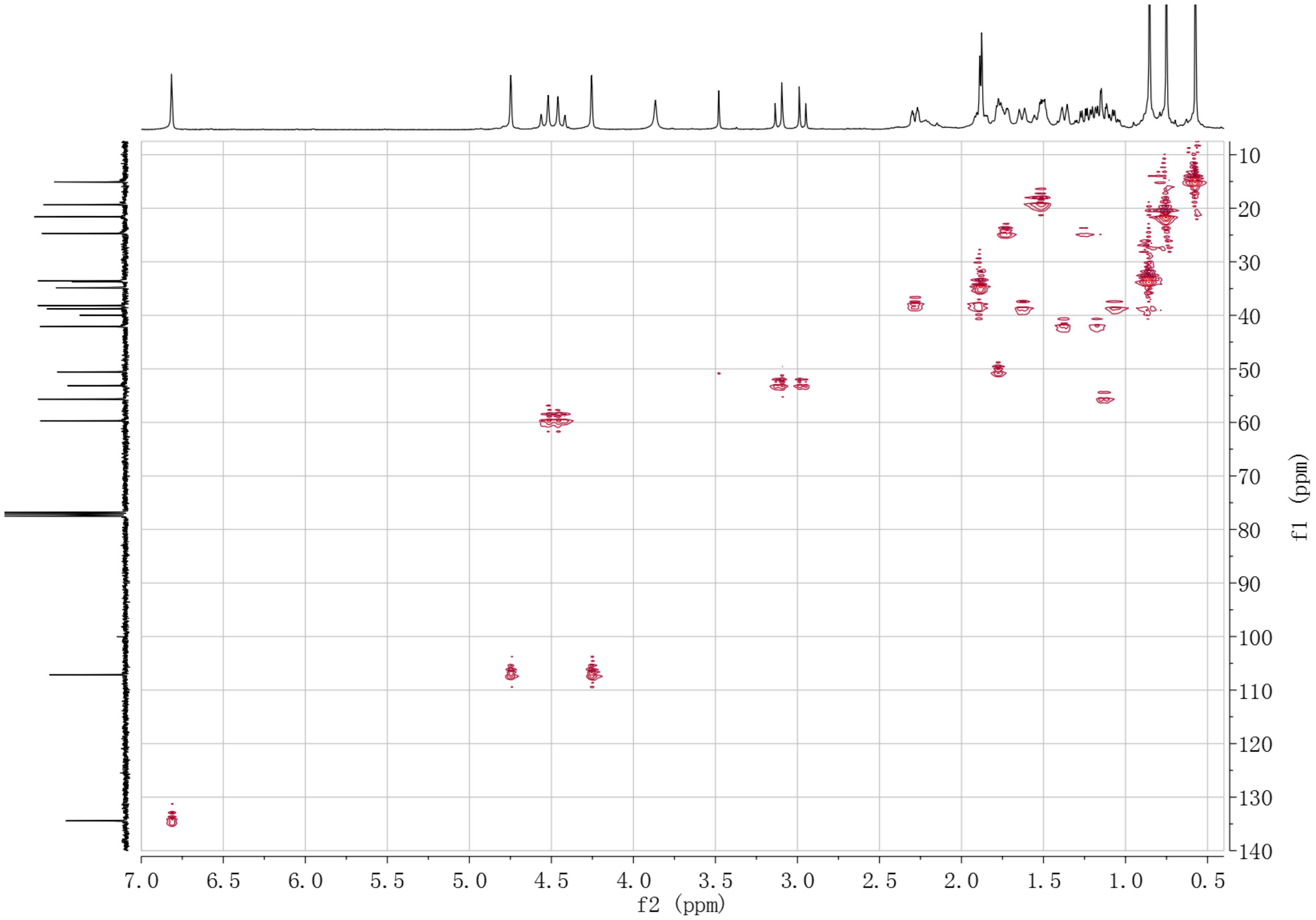

Expanded HMQC Spectrum of **2** in $CDCl_3$

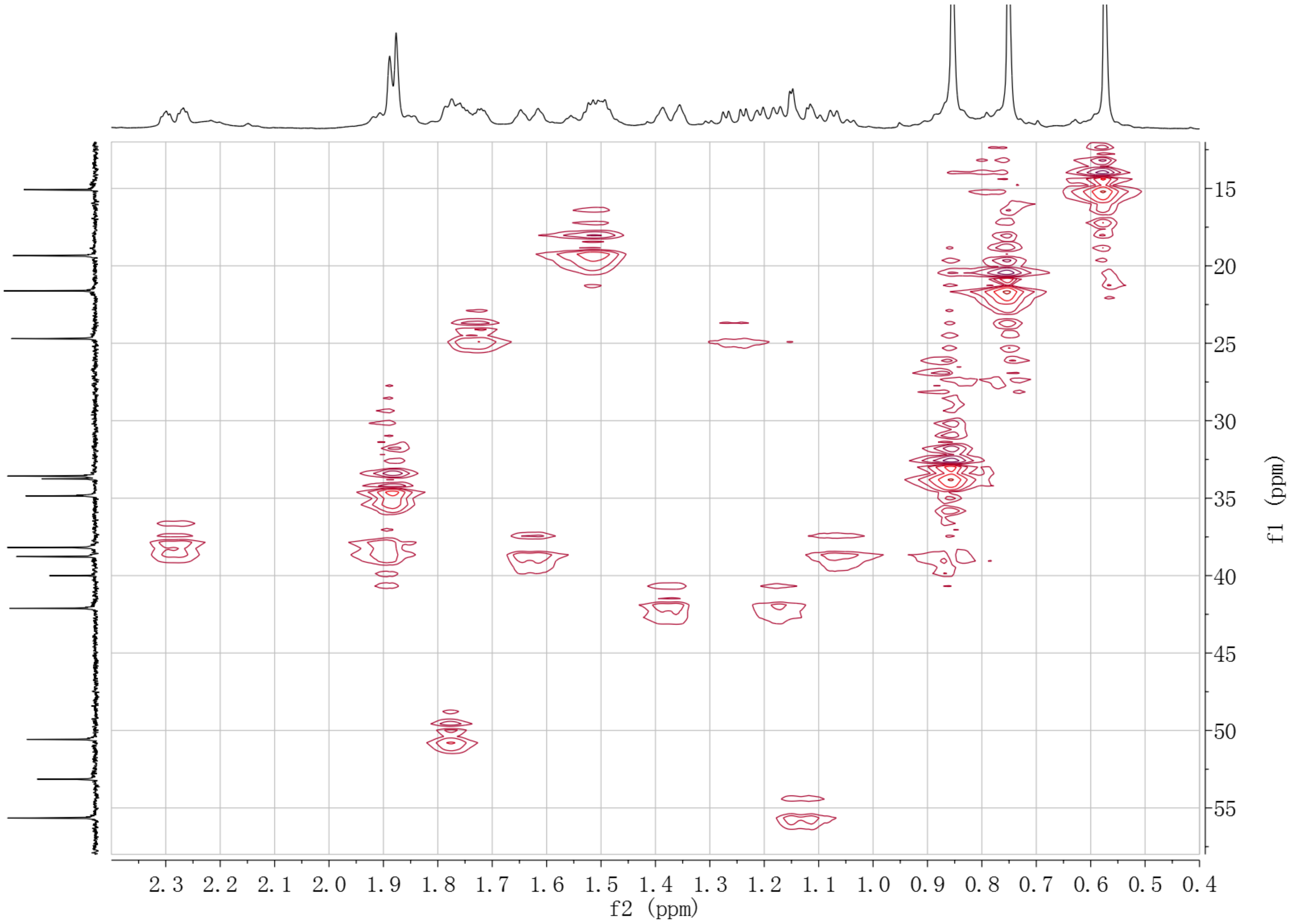

HMBC Spectrum of **2** in $CDCl_3$

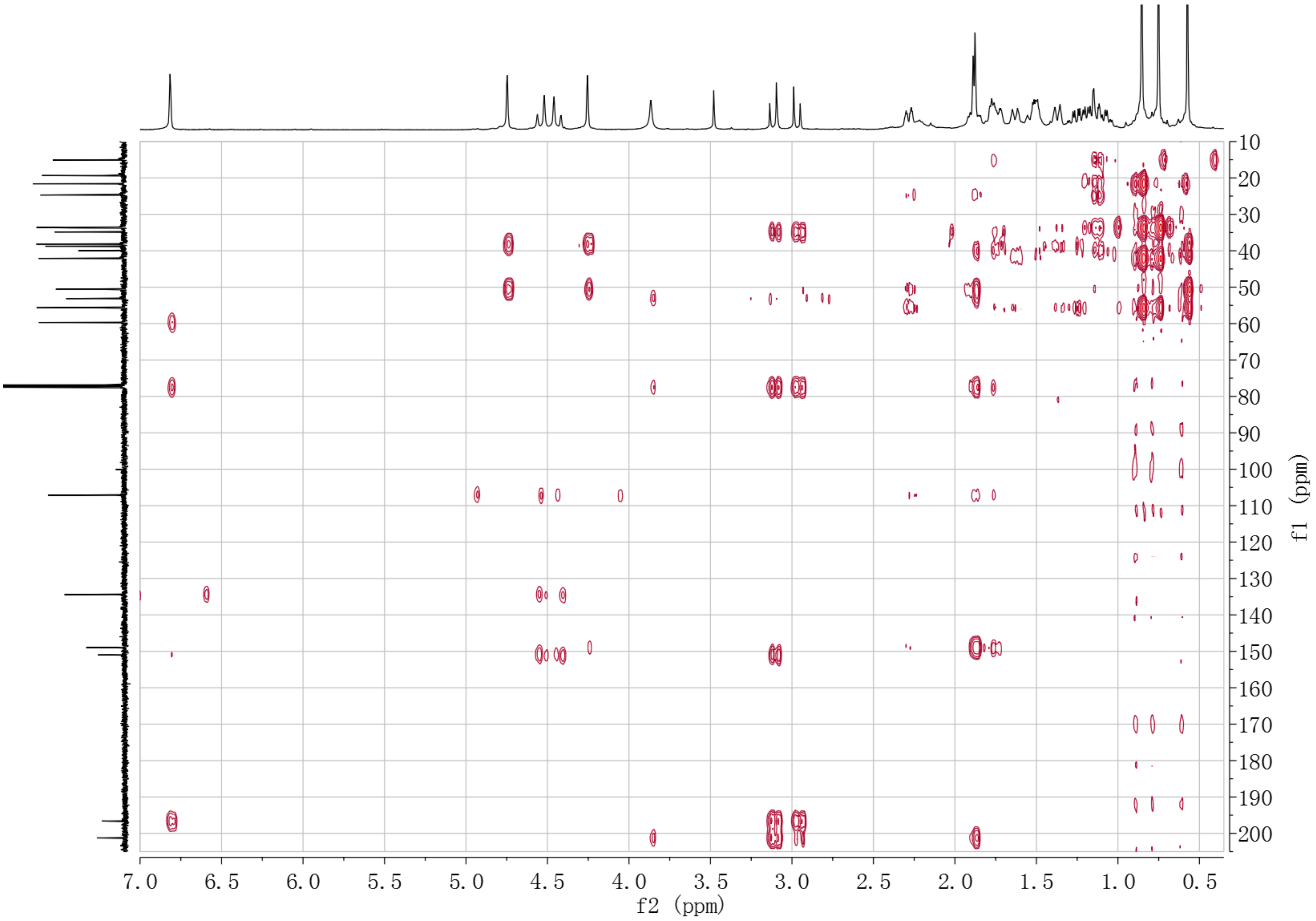

Expanded HMBC Spectrum of **2** in $CDCl_3$

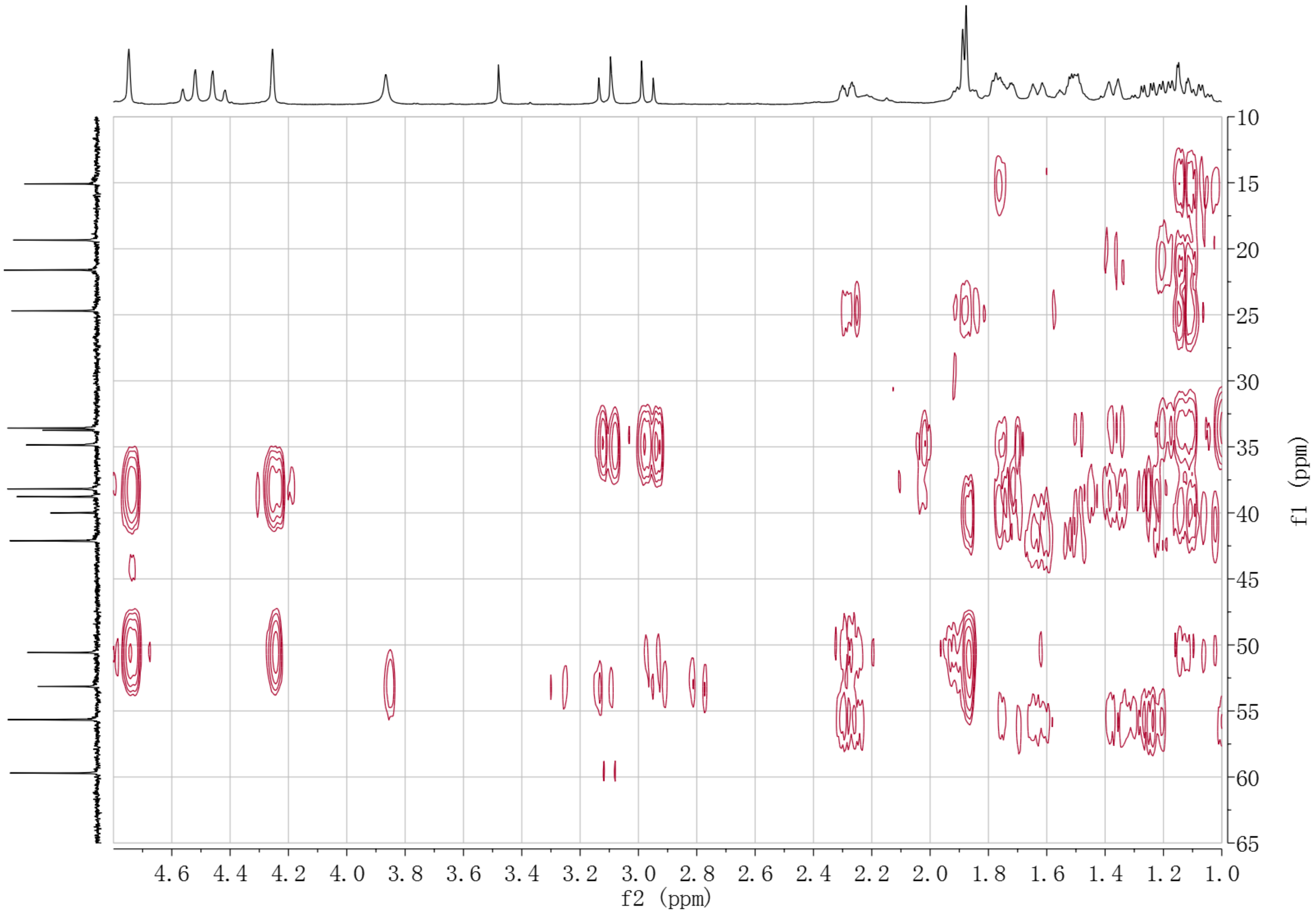

Expanded HMBC Spectrum of **2** in $CDCl_3$

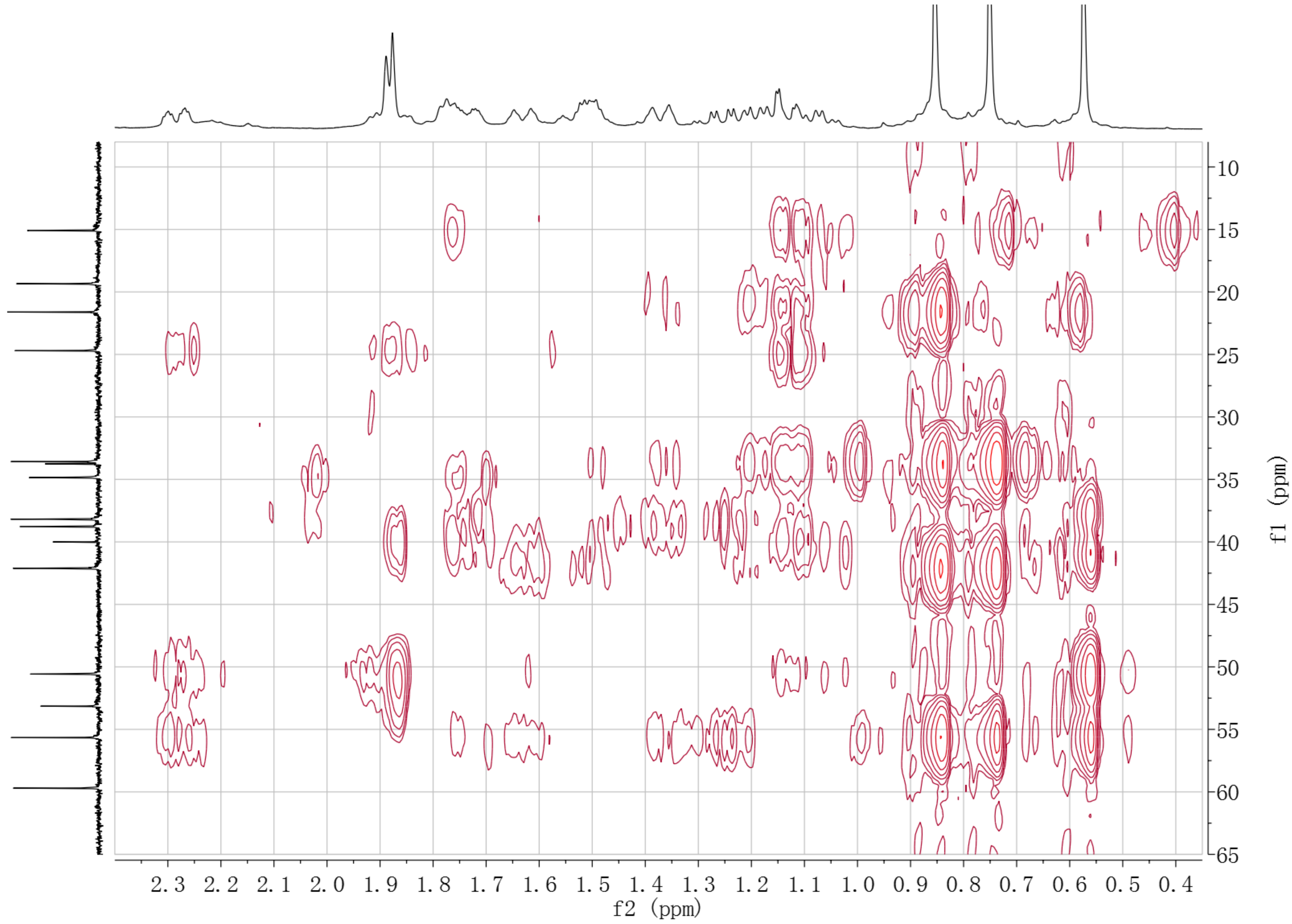

NOESY Spectrum of **2** in $CDCl_3$

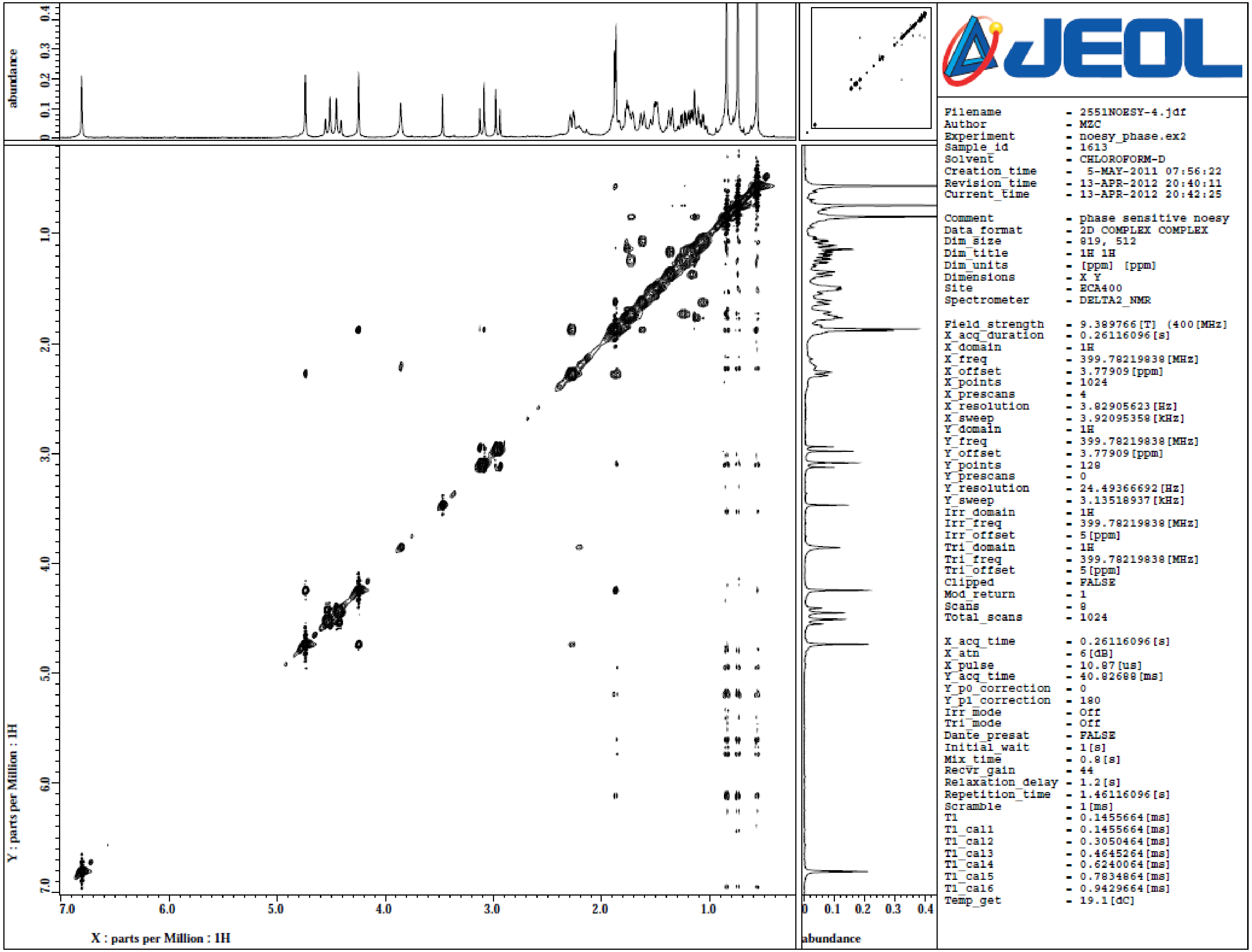

Expanded NOESY Spectrum of **2** in $CDCl_3$

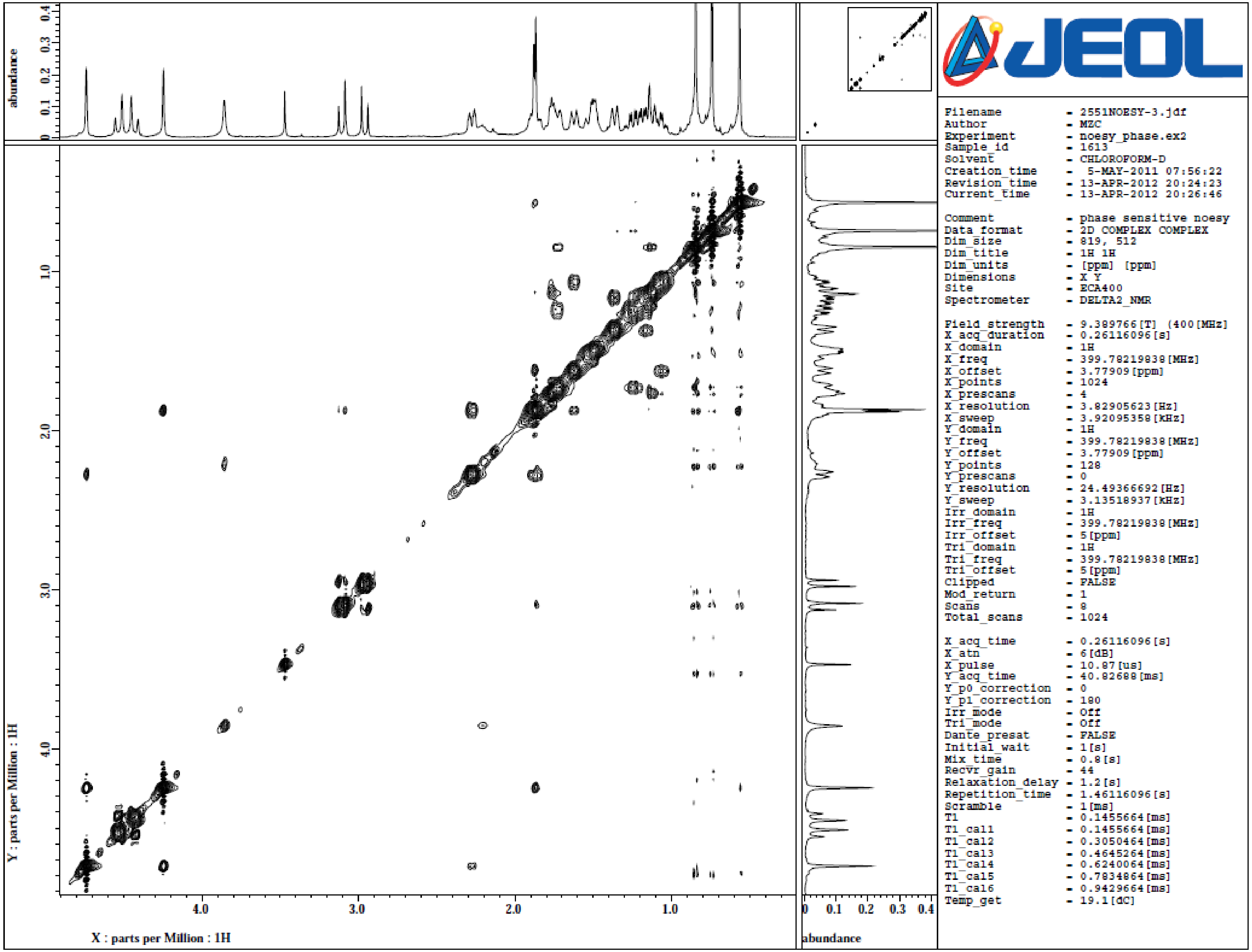

Expanded NOESY Spectrum of **2** in $CDCl_3$

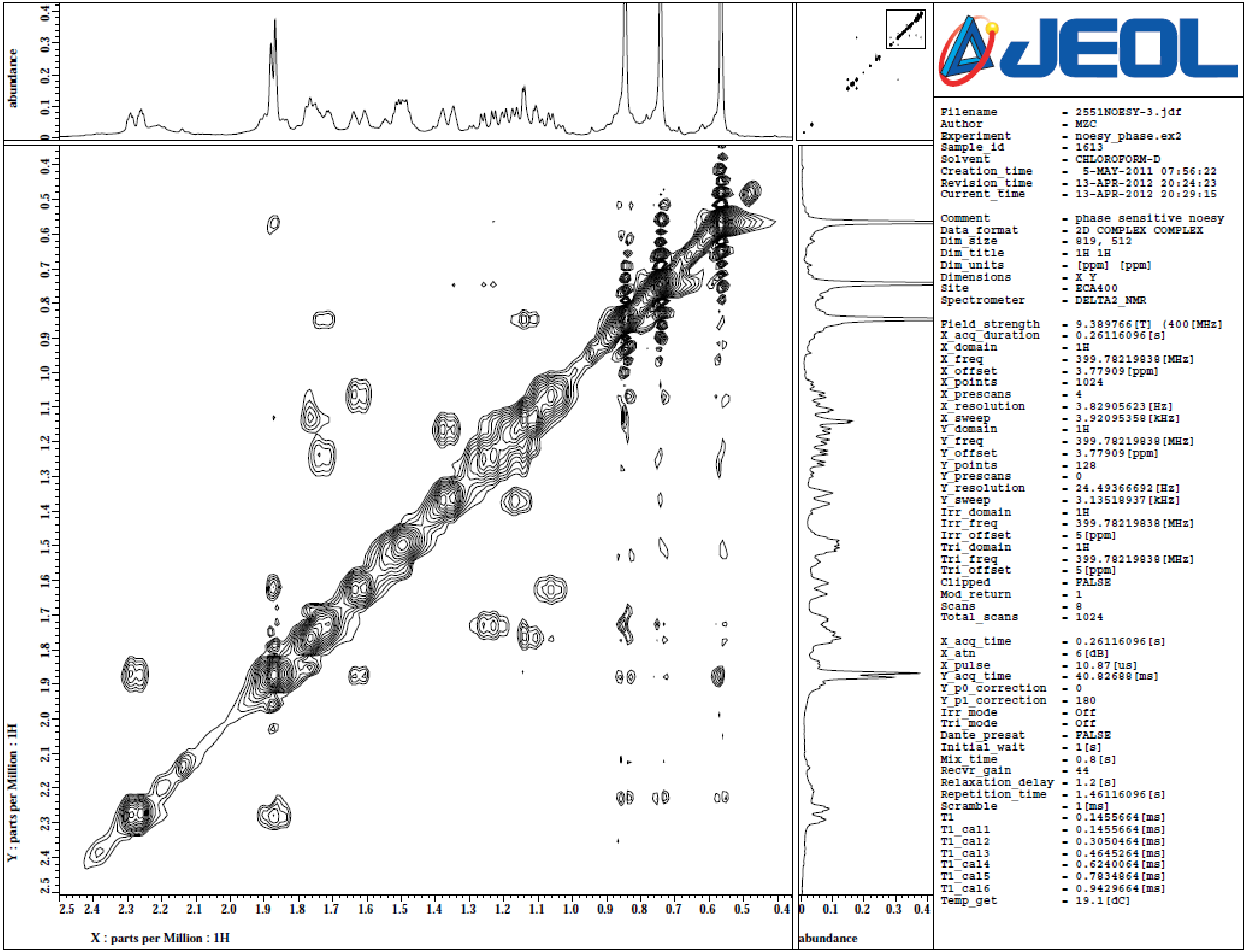

Supplement: Supplementary File 1: — ZIP-Document (ZIP, 8582 KB) [file marinedrugs-10-01266-s001.zip › Supplementary Data S3_MS, UV, IR and NMR spectra of 2.pdf]
