# Supplementary material for: Purpurogemutantin and Purpurogemutantidin, New Drimenyl Cyclohexenone Derivatives Produced by a Mutant Obtained by Diethyl Sulfate Mutagenesis of a Marine-Derived Penicillium purpurogenum G59
Source: Mar Drugs. 2012 Jun 4;10(6):1266–87. doi: 10.3390/md10061266 (PMC3397438; doi:10.3390/md10061266)

Supplementary Materials

Supplementary Data S4. HPLC Analysis for 1–3 and the G59 and BD-1-6 Extracts

Contents

Content	Page
1. Conditions for the HPLC analysis	1
2. HPLC profiles detected at 210 nm	2
UV absorption curves of **1**–**3** and the BD-1-6 and G59 extracts at the indicated t_R	2
UV absorption curves of the peaks in HPLC profiles of BD-1-6 and G59 at the indicated t_R	3
3. HPLC profiles detected at 236 nm	5
4. HPLC profiles detected at 254 nm	7
5. HPLC profiles detected at 300 nm	7
6. HPLC profiles detected at 327 nm	8

1. Conditions for the HPLC analysis

The HPLC analysis was performed using a Waters HPLC equipment using Waters 600 controller, Waters 600 pump, Waters 2414 refractive index detector, Waters 2996 photodiode array detector and Waters Empower™ software.

The EtOAc extracts of G59 and BD-1-6 cultures and the compounds **1**–**3** were dissolved in MeOH to prepare a 10 mg/ml sample solution for HPLC analysis. HPLC analysis of the samples was carried out on a Venusil MP C18 column (5 μm, 100 Å, 4.6 mm × 250 mm; Agela Technologies) using the same Waters HPLC equipment mentioned. Each 5 μl of sample solutions was injected into the column after filtration by a 0.22 μm pore membrane filter. Then, the elution was performed using MeOH–H_2O in linear gradient (20% MeOH at initial time 0 min→ 100% MeOH at 60 min→ 100% MeOH at 90 min; flow rate, 1 ml/min). The acquired photodiode array (PDA) data were processed by Empower PDA software to obtain targeted HPLC data.

2. HPLC profiles detected at 210 nm

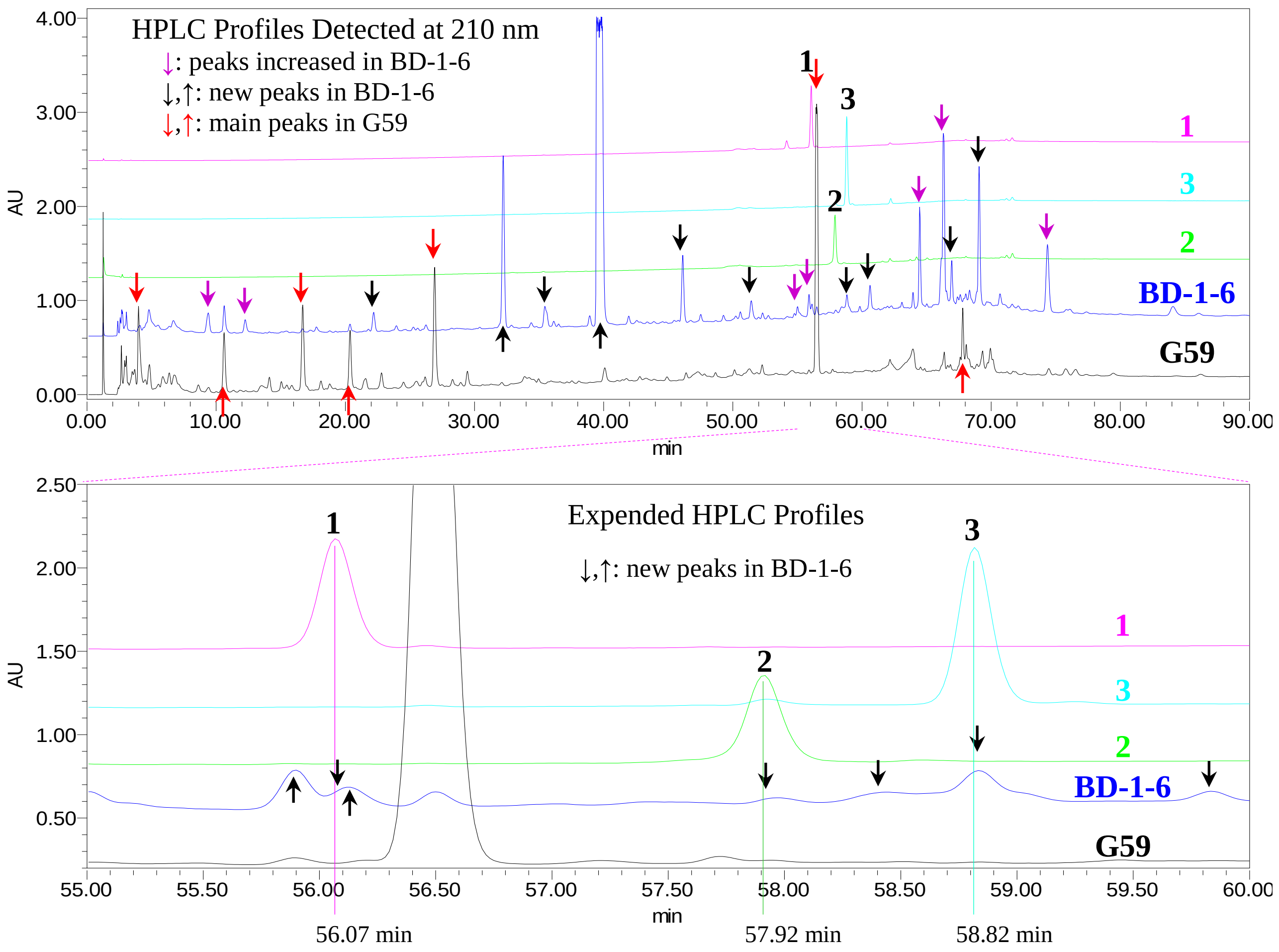

UV absorption curves of **1**–**3** and the BD-1-6 and G59 extracts at the indicated t_R

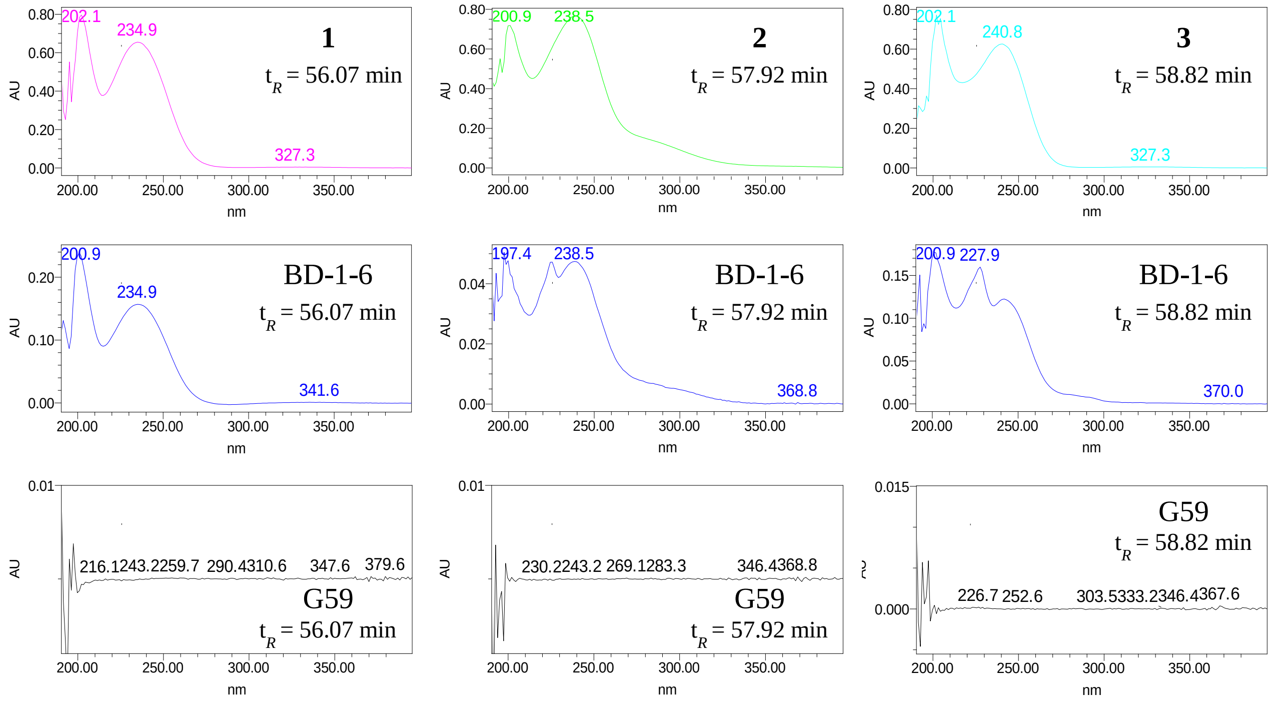

UV absorption curves of the peaks in HPLC profiles of BD-1-6 and G59 at the indicated t_R

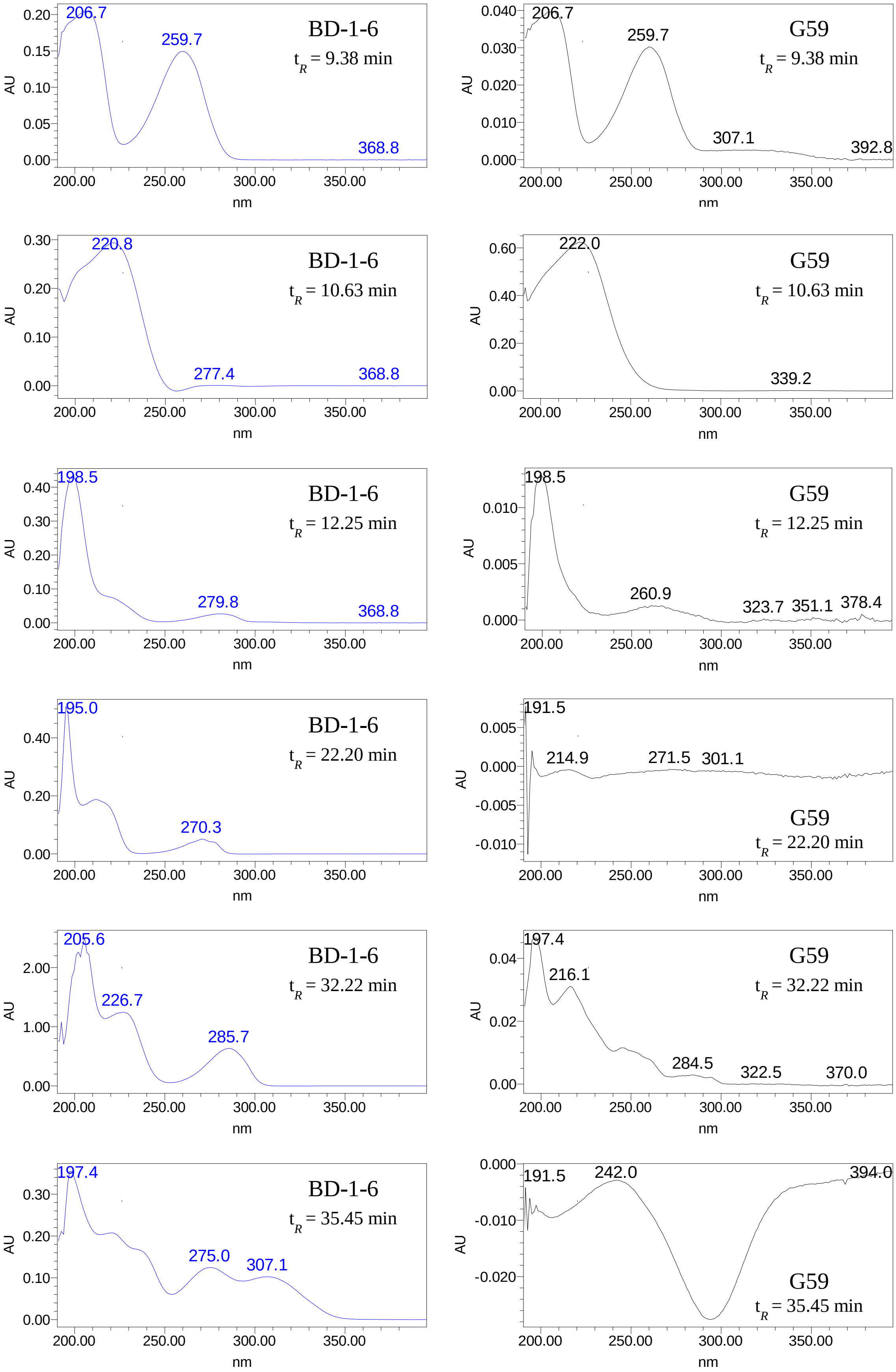

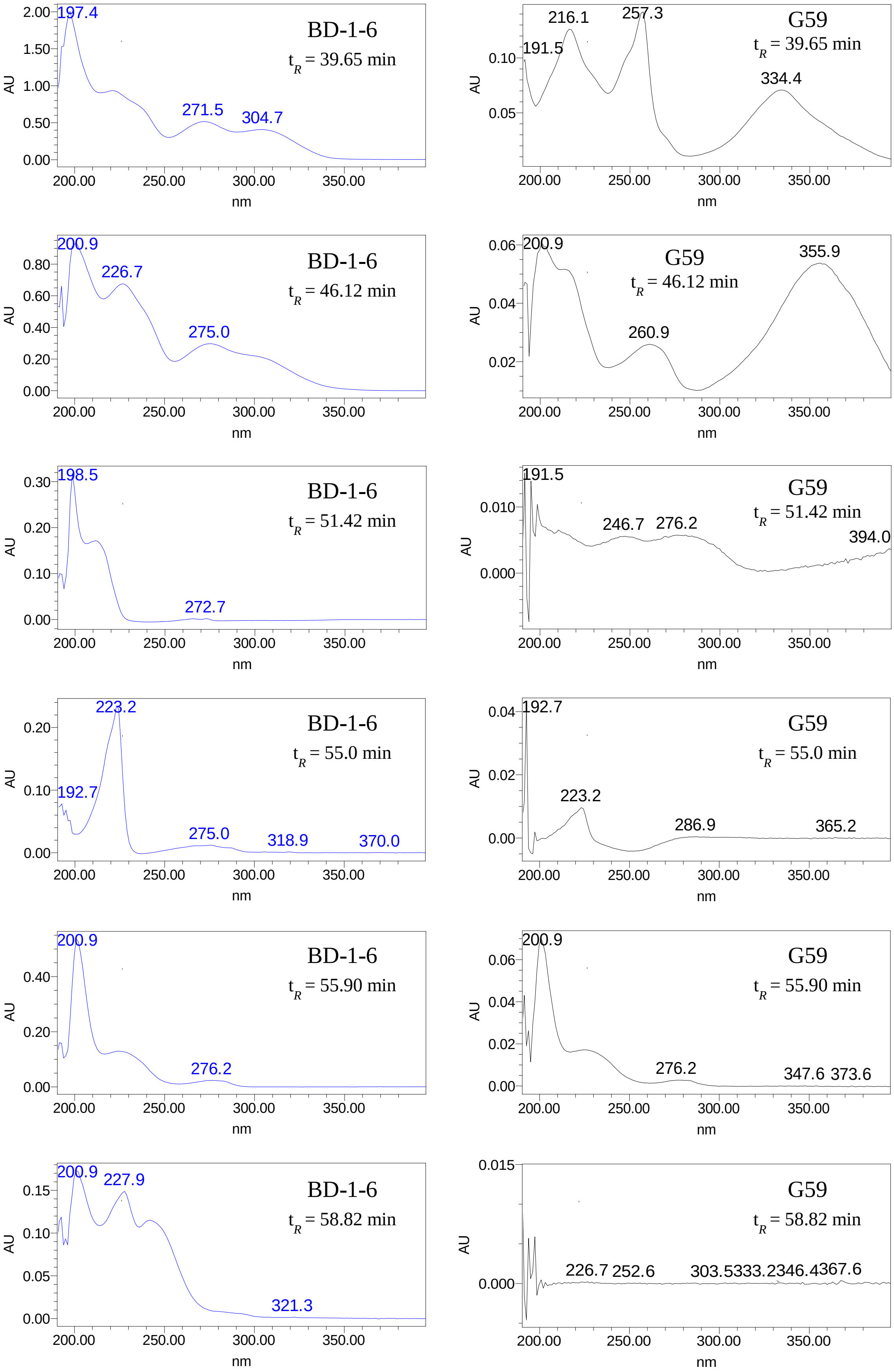

BD-1-6
t_R = 39.65 min
197.4
271.5
304.7
G59
t_R = 39.65 min
191.5
216.1
257.3
334.4
BD-1-6
t_R = 46.12 min
200.9
226.7
275.0
G59
t_R = 46.12 min
200.9
260.9
355.9
BD-1-6
t_R = 51.42 min
198.5
272.7
G59
t_R = 51.42 min
191.5
246.7
276.2
394.0
BD-1-6
t_R = 55.0 min
192.7
223.2
275.0
318.9
370.0
G59
t_R = 55.0 min
192.7
223.2
286.9
365.2
BD-1-6
t_R = 55.90 min
200.9
276.2
G59
t_R = 55.90 min
200.9
276.2
347.6 373.6
BD-1-6
t_R = 58.82 min
200.9
227.9
321.3
G59
t_R = 58.82 min
226.7 252.6
303.5333.2346.4367.6
AU
nm
200.00
250.00
300.00
350.00

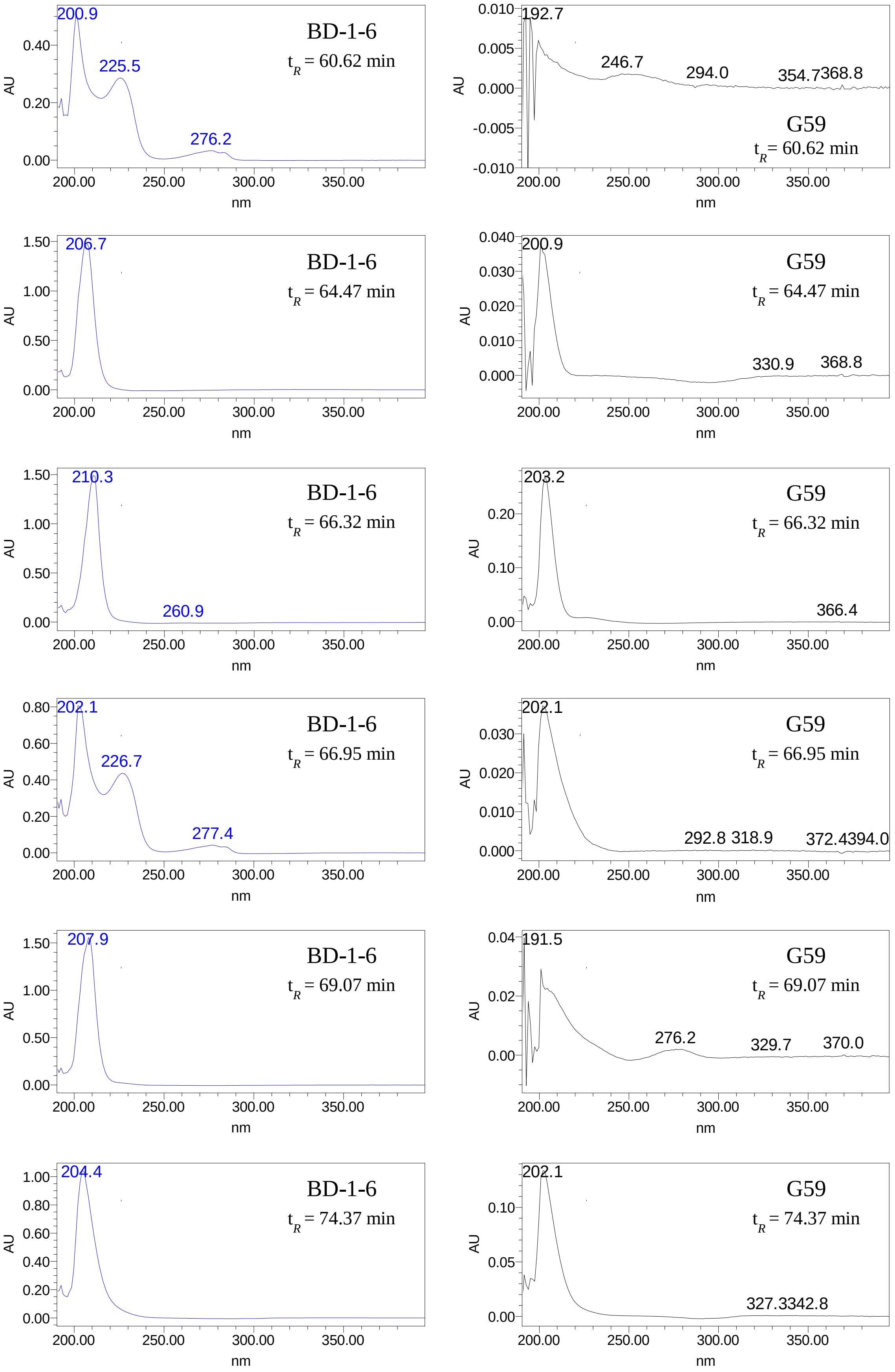

3. HPLC profiles detected at 236 nm

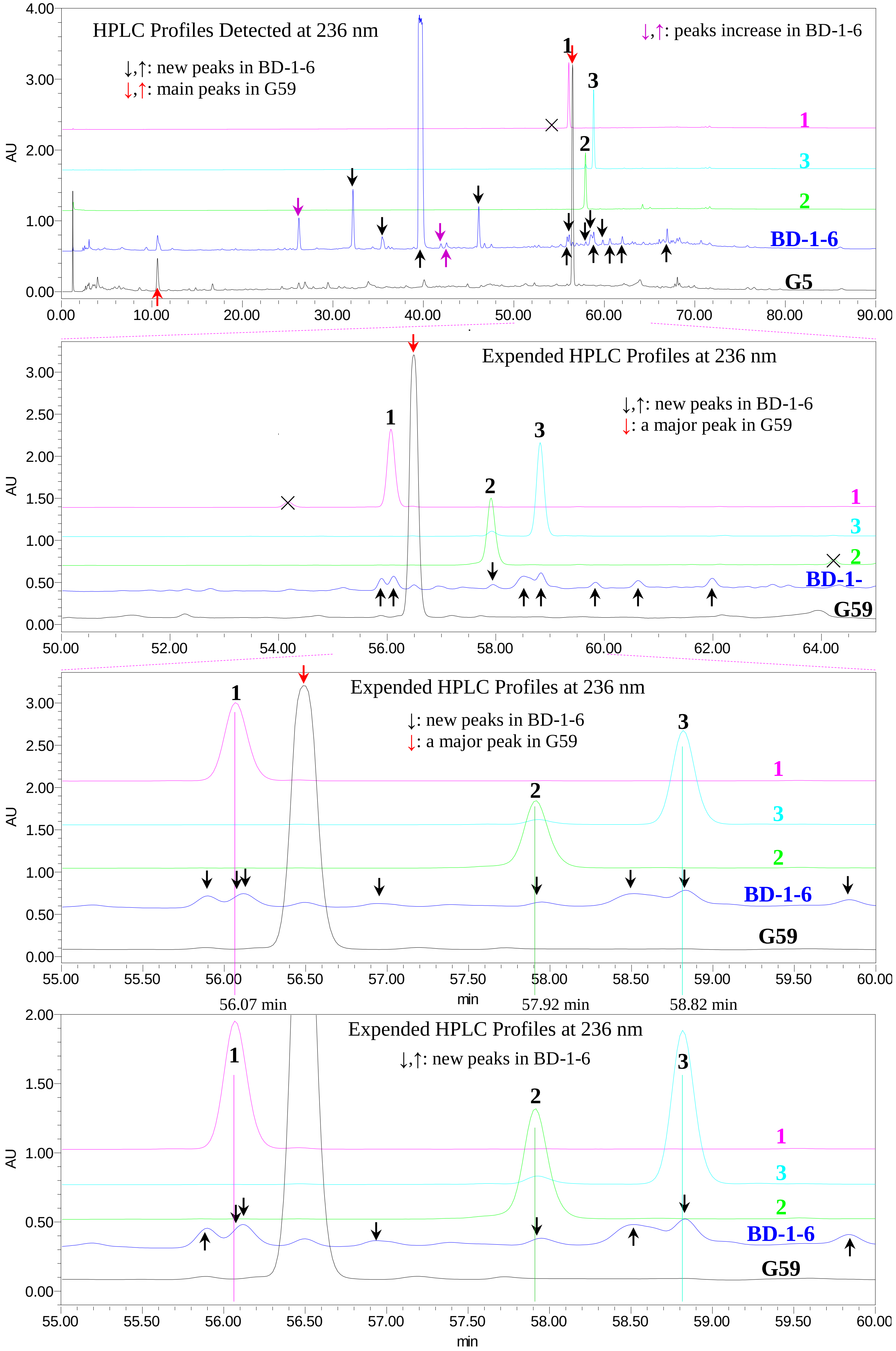

HPLC Profiles Detected at 236 nm
↓,↑: new peaks in BD-1-6
↓,↑: main peaks in G59
↓,↑: peaks increase in BD-1-6
1
2
3
BD-1-6
G5
AU
Expended HPLC Profiles at 236 nm
↓,↑: new peaks in BD-1-6
↓: a major peak in G59
BD-1-
G59
Expended HPLC Profiles at 236 nm
↓: new peaks in BD-1-6
↓: a major peak in G59
56.07 min
57.92 min
58.82 min
min
Expended HPLC Profiles at 236 nm
↓,↑: new peaks in BD-1-6
min

4. HPLC profiles detected at 254 nm

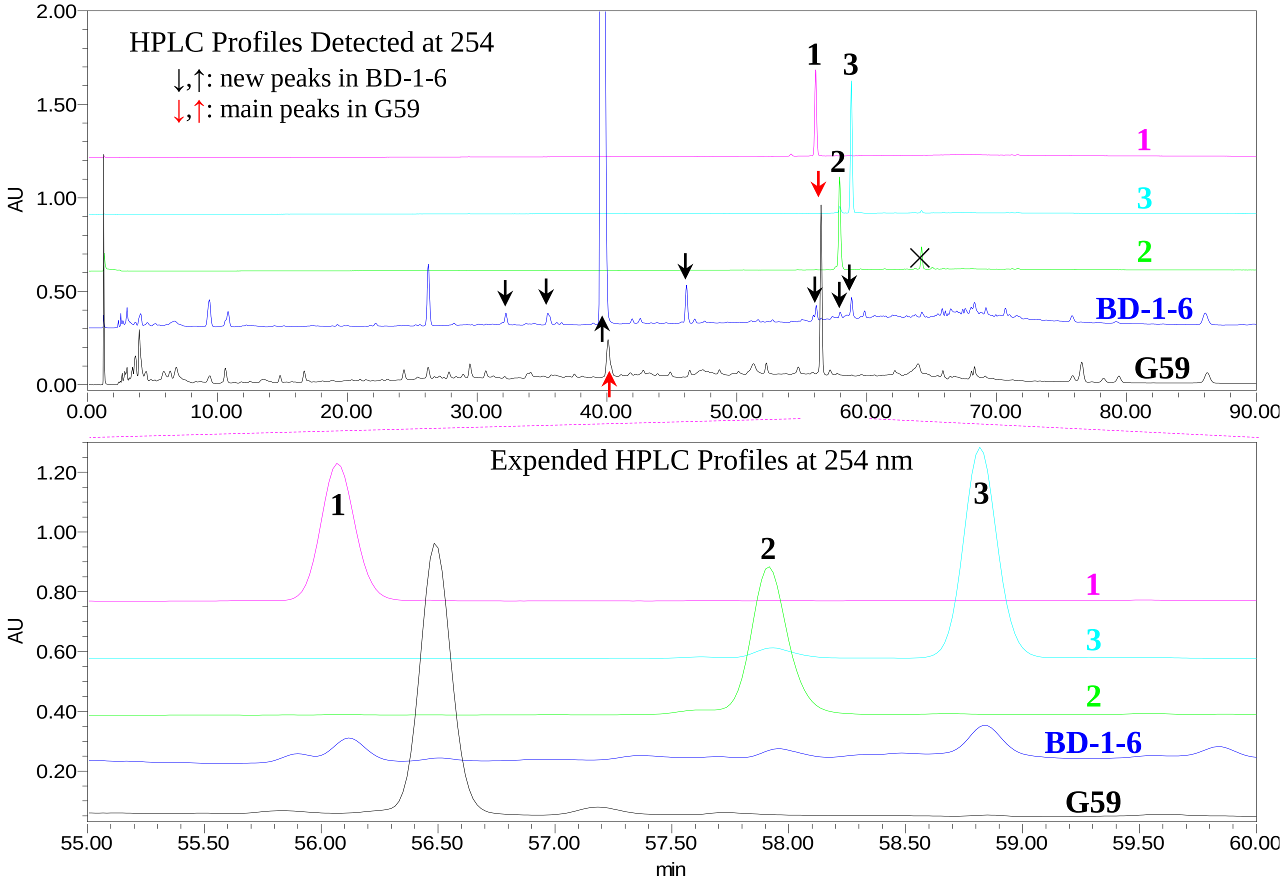

4. HPLC profiles detected at 300 nm

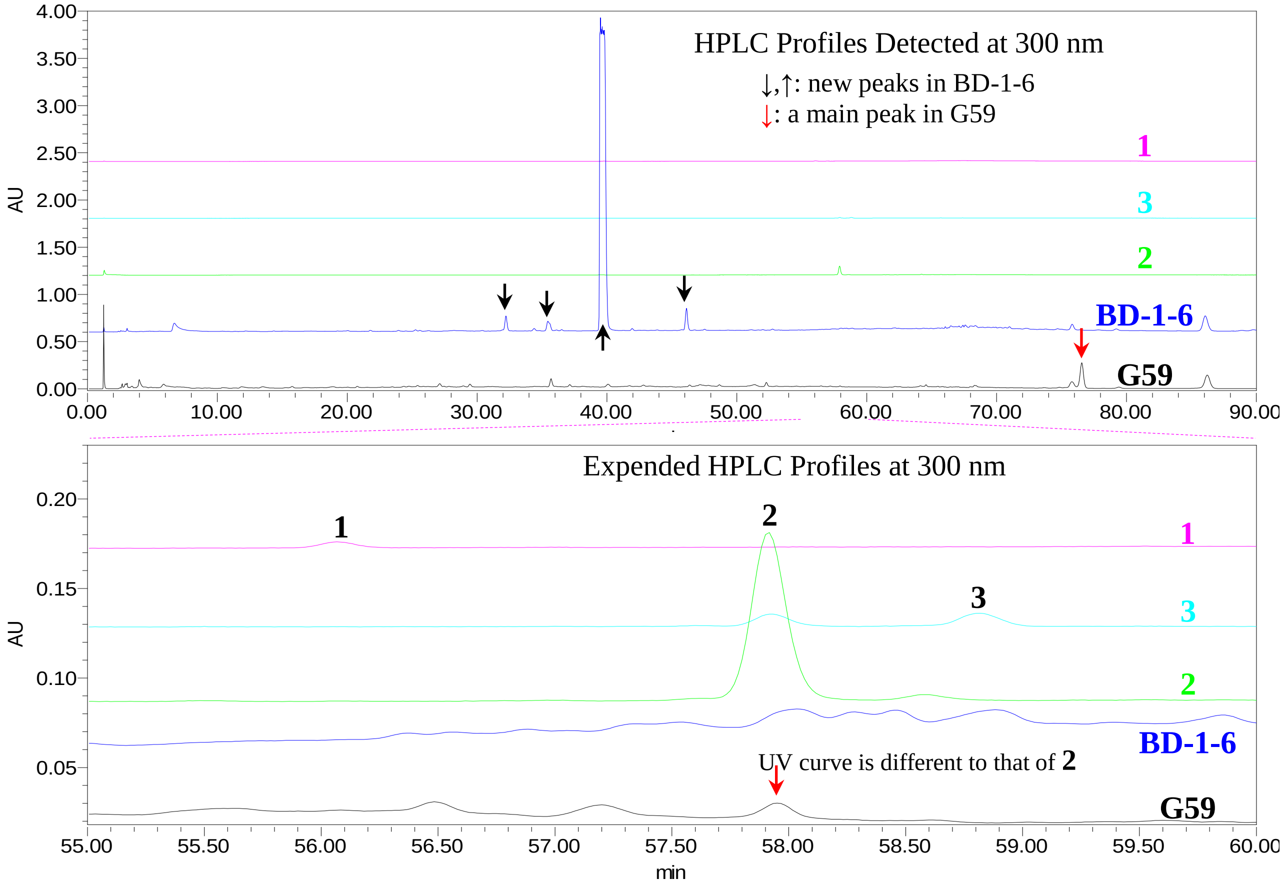

4. HPLC profiles detected at 327 nm

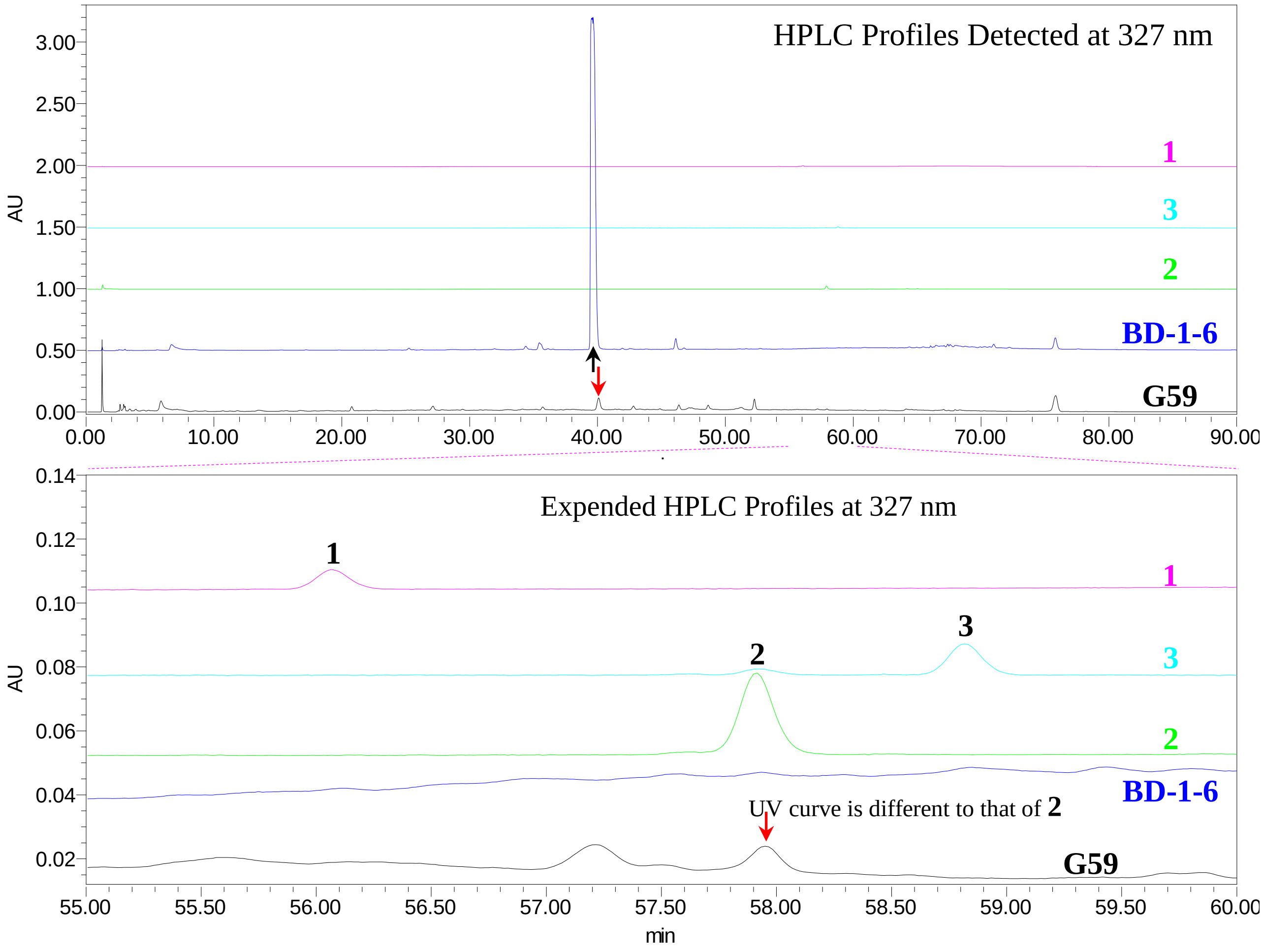

Supplement: Supplementary File 1: — ZIP-Document (ZIP, 8582 KB) [file marinedrugs-10-01266-s001.zip › Supplementary Data S4_HPLC analysis for 1¿C3.pdf]
