# Supplementary material for: Purpurogemutantin and Purpurogemutantidin, New Drimenyl Cyclohexenone Derivatives Produced by a Mutant Obtained by Diethyl Sulfate Mutagenesis of a Marine-Derived Penicillium purpurogenum G59
Source: Mar Drugs. 2012 Jun 4;10(6):1266–87. doi: 10.3390/md10061266 (PMC3397438; doi:10.3390/md10061266)

Supplementary Materials

Supplementary Data S5. LC-ESIMS Data for Detecting 1-3 in EtOAc Extracts of G59 and Its Mutant BD-1-6

Contents

Content	Page
1. Conditions and conclusions of LC-ESIMS analysis to detect compounds **1** - **3**	1
2. LC-ESIMS data for detecting **1** in parent G59 and its mutant BD-1-6 samples	2
3. LC-ESIMS data for detecting **2** in parent G59 and its mutant BD-1-6 samples	4
4. LC-ESIMS data for detecting **3** in parent G59 and its mutant BD-1-6 samples	5

1. Conditions and conclusions of LC-ESIMS analysis to detect compounds **1-3**

Conditions: LC-ESIMS analysis was performed on a LC-MS equipment equipped with Agilent 1100 HPLC system, AB Sciex API 3000 LC-MS/MS system, and AB Sciex Analyst 1.4 software. EtOAc extracts of G59 and BD-1-6 were employed for the LC-ESIMS analysis and crude **1**–**3** samples were used as control. HPLC was carried out on a Venusil MP C18 column (5 μm, 100 Å, 4.6 mm × 250 mm; Agela Technologies) using MeOH–H_2O in linear gradient (20% MeOH at initial time 0 min→ 100% MeOH at 60 min→ 100% MeOH at 90 min; flow rate, 1 ml/min) as mobile phase. The mass detector was set to scan a range from *m/z* 150 to 1500 in positive (for **1**–**3**) or negative (for **1**) mode. The acquired data were processed by Analyst 1.4 software to obtain targeted LC-ESIMS data.

Conclusions: The $[M+Na]^+$ or $[M-H]^-$ ions of **1**, **2**, and **3** were appeared as peaks with retention times of 52.3, 53.8, and 55.1 min, respectively. Compounds **1**–**3** in BD-1-6 and G59 were examined by using selective ion monitoring and all of **1-3** were detected only in BD-1-6 but entirely not in G59 samples.

LC-ESIMS Data for Detecting **1** ($[M+Na]^+$ ion) in Parent G59 and Its Mutant BD-1-6 Samples

Positive Selective Ion (*m/z* 441.0-442.0) Monitoring Chromatograms

ESIMS Spectra in the Range of 51.999-53.003 min Retention Times

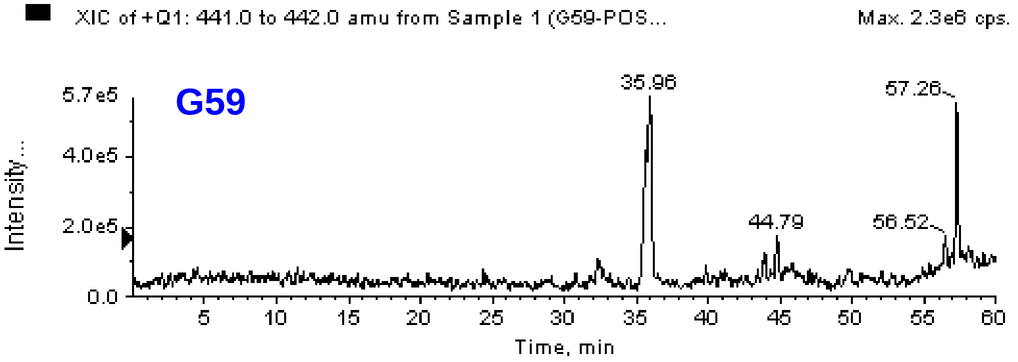

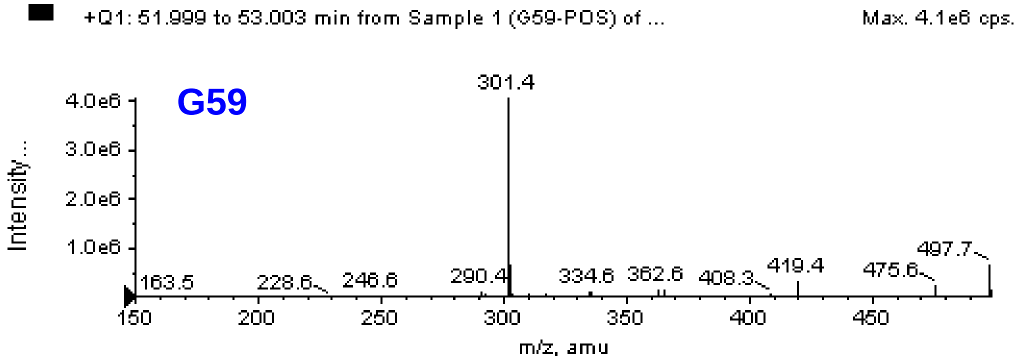

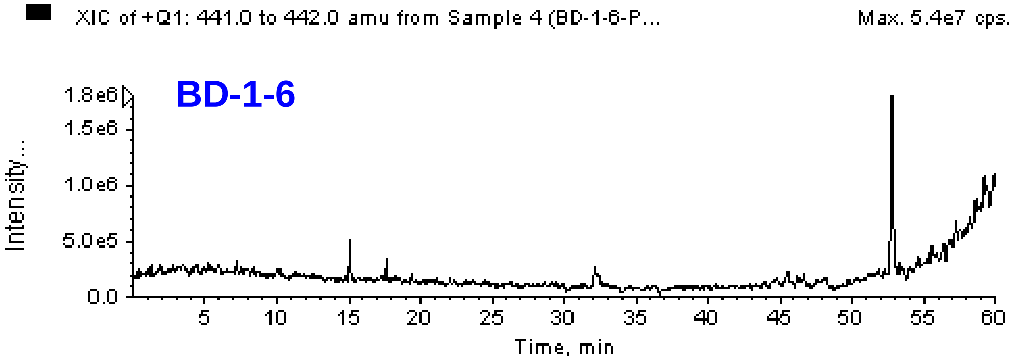

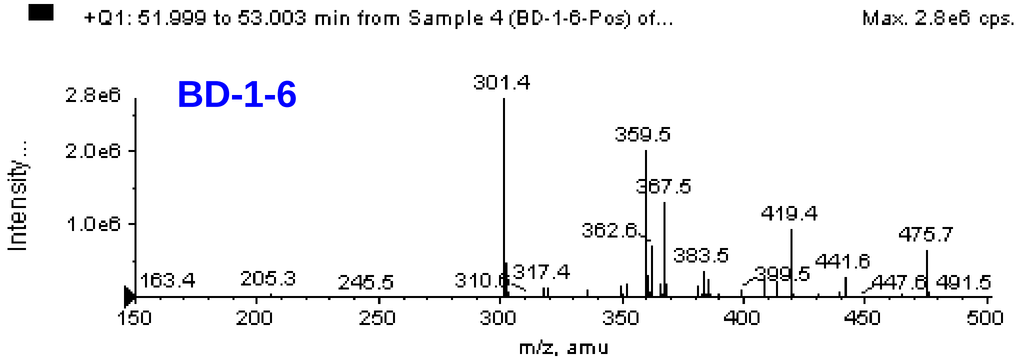

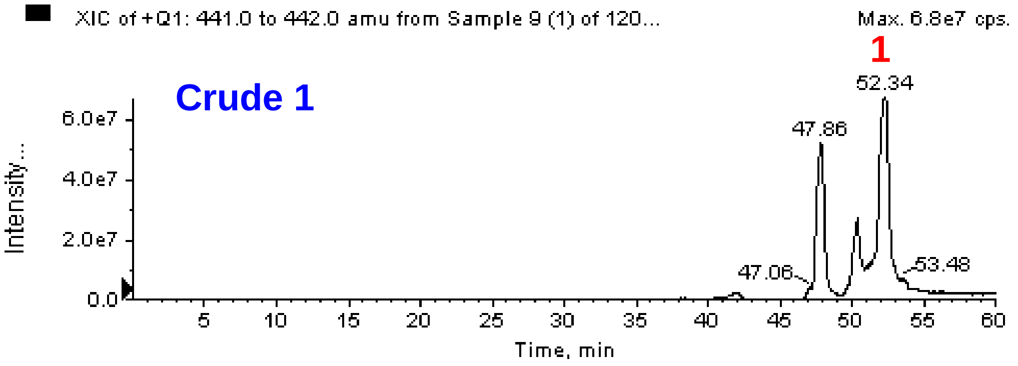

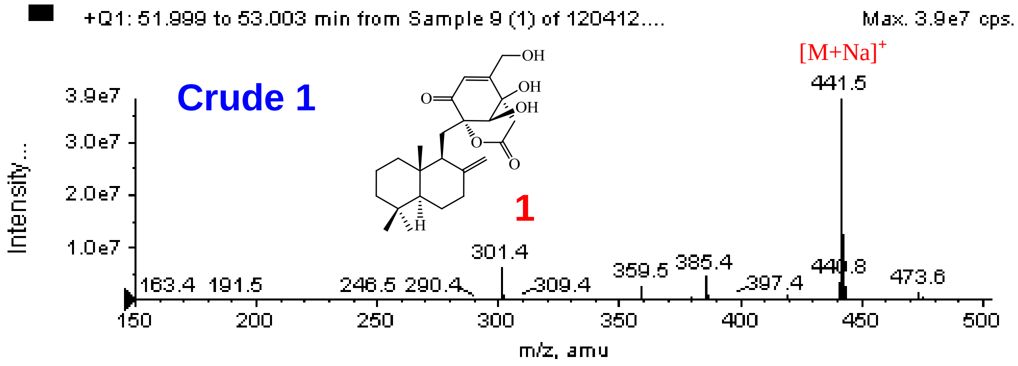

LC-ESIMS Data for Detecting **1** ([M-H]$^-$ ion) in Parent G59 and Its Mutant BD-1-6 Samples

Negative Selective Ion (*m/z* 417.0-418.0) Monitoring Chromatograms

ESIMS Spectra in the Range of 51.999-53.003 min Retention Times

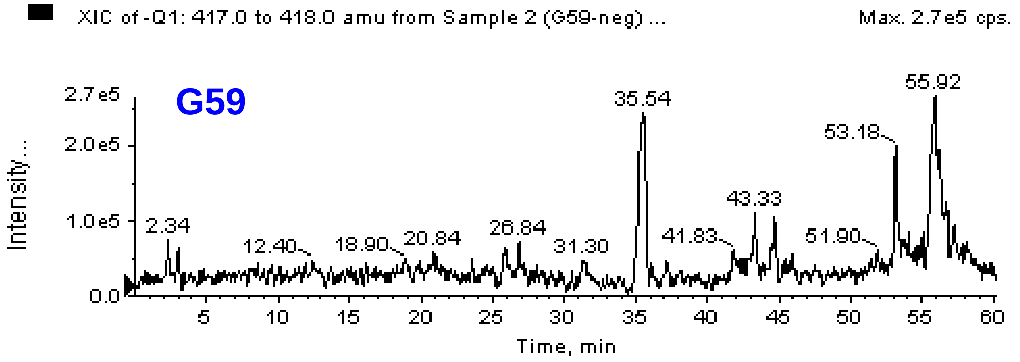

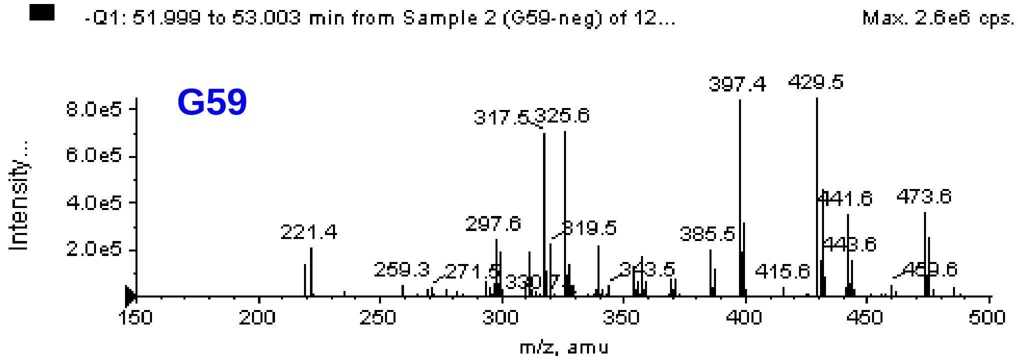

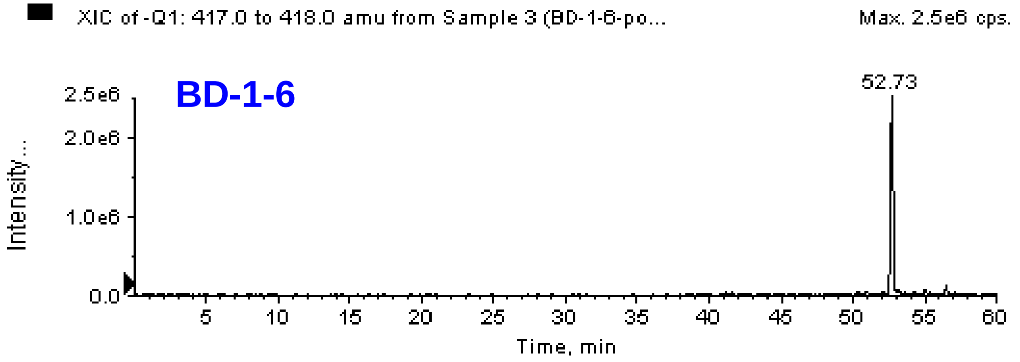

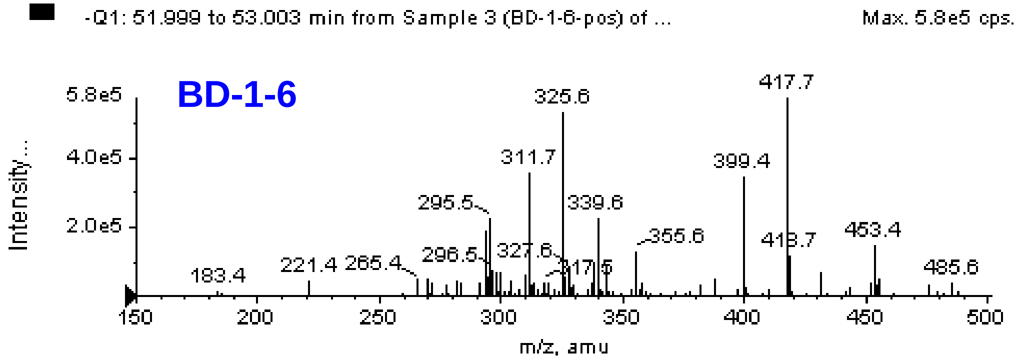

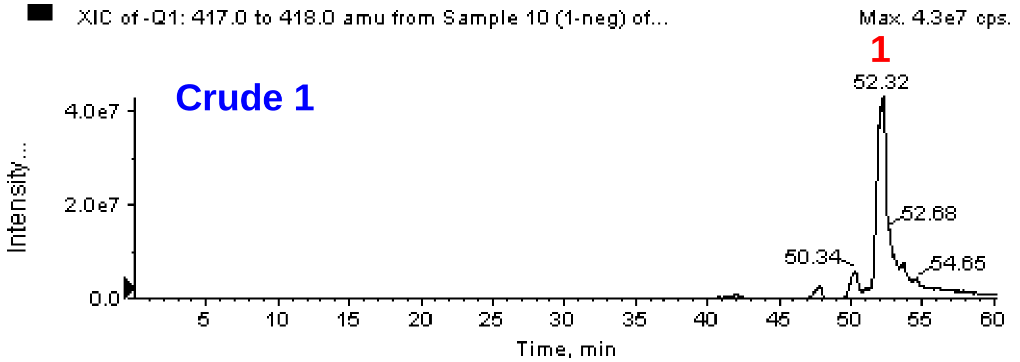

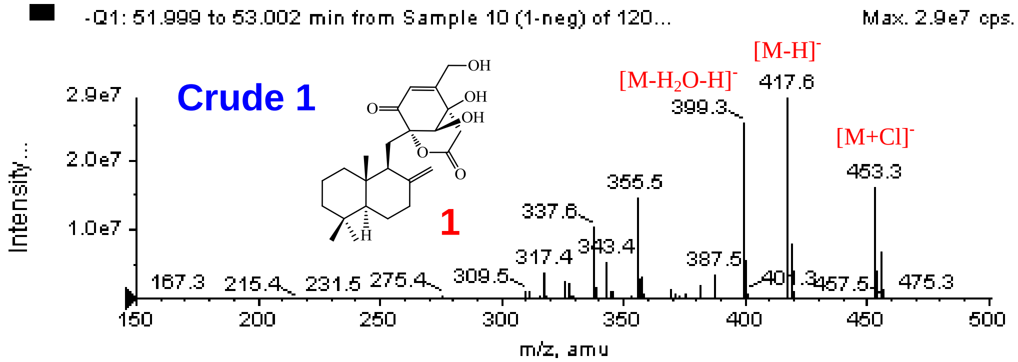

LC-ESIMS Data for Detecting **2** ([M+Na]$^+$ ion) in Parent G59 and Its Mutant BD-1-6 Samples

Positive Selective Ion (*m/z* 383.0-384.0) Monitoring Chromatograms | **ESIMS Spectra in the Range of 53.605-54.608 min Retention Times**

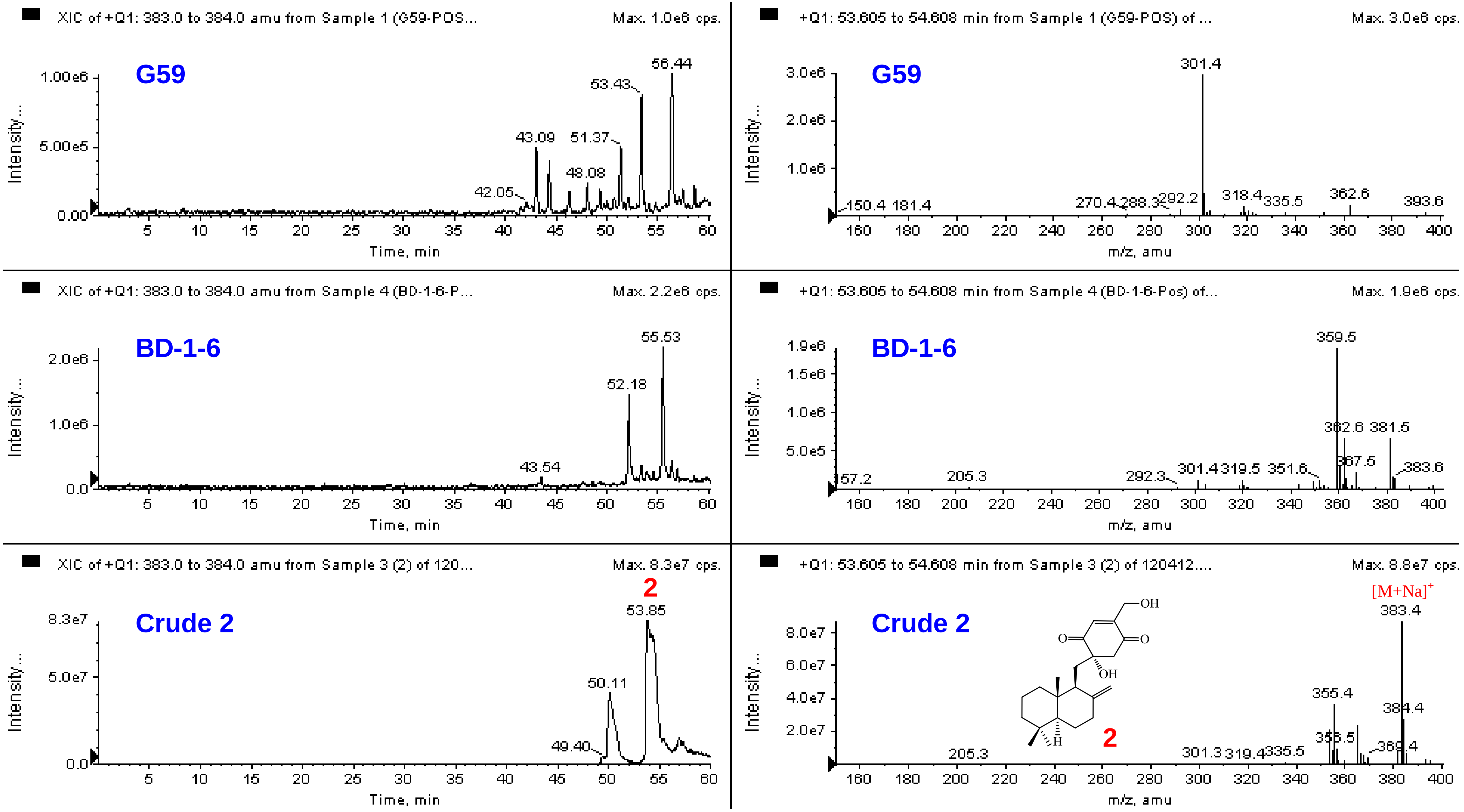

LC-ESIMS Data for Detecting **3** ([M+Na]$^+$ ion) in Parent G59 and Its Mutant BD-1-6 Samples

Positive Selective Ion (*m/z* 383.0-384.0) Monitoring Chromatograms | **ESIMS Spectra in the Range of 54.708-55.511 min Retention Times**

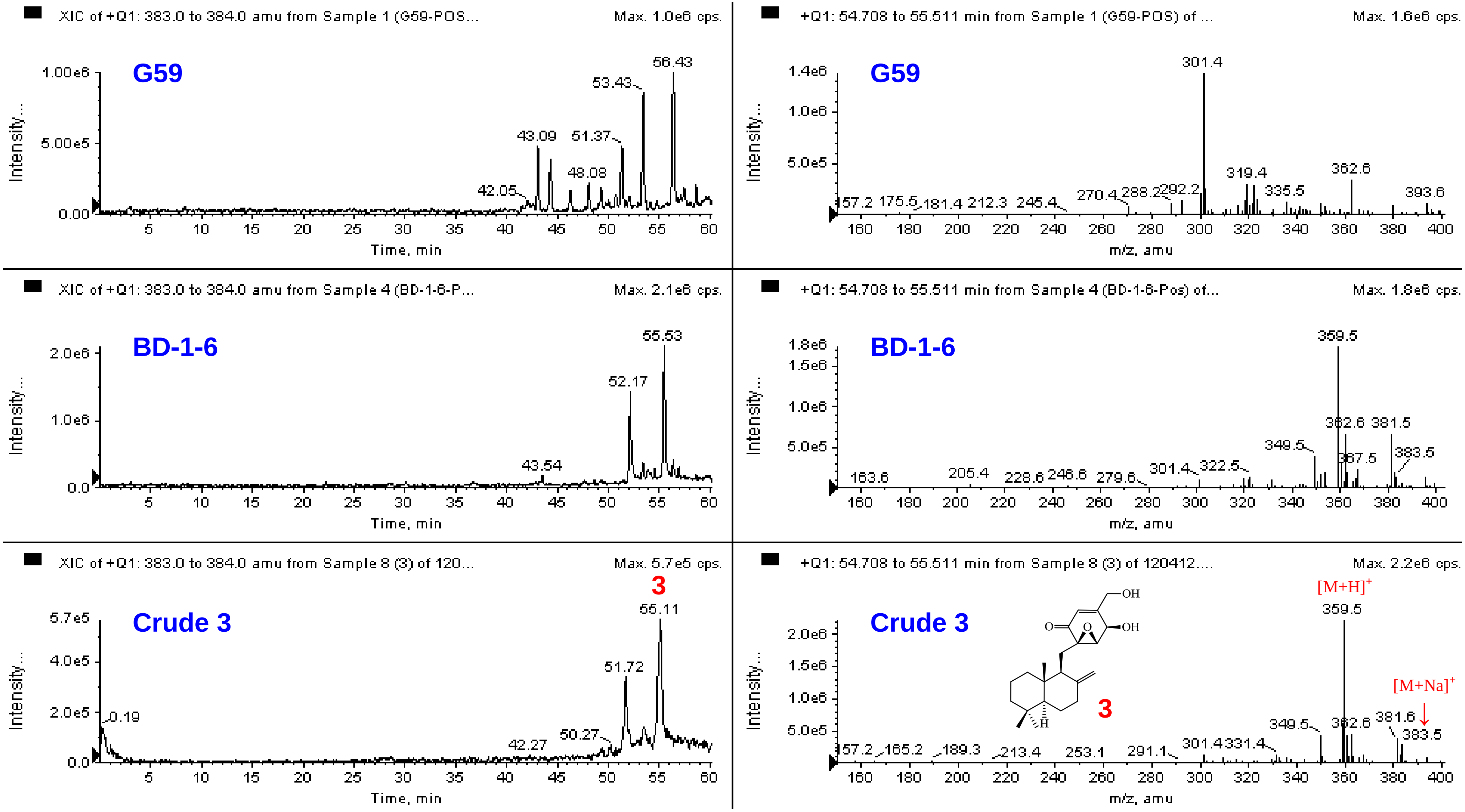

Supplement: Supplementary File 1: — ZIP-Document (ZIP, 8582 KB) [file marinedrugs-10-01266-s001.zip › Supplementary Data S5_LC-MS data for detcting 1-3.pdf]
